# Circadian oscillator proteins across the kingdoms of life: structural aspects

**DOI:** 10.1186/s12915-018-0623-3

**Published:** 2019-02-18

**Authors:** Reena Saini, Mariusz Jaskolski, Seth J. Davis

**Affiliations:** 1 Center for Biocrystallographic Research, Institute of Bioorganic Chemistry, Polish Academy of Sciences, Poznan, Poland; 2 Department of Crystallography, Faculty of Chemistry, A. Mickiewicz University, Poznan, Poland; 3 Max-Planck-Institut für Pflanzenzüchtungsforschung, Cologne, Germany; 4 Department of Biology, University of York, York, UK

**Keywords:** Circadian rhythms, Clock genes, Feedback loops, Transcription factors, Homo- and heteroprotein complexes, Phosphorylation, Crystallography

## Abstract

Circadian oscillators are networks of biochemical feedback loops that generate 24-hour rhythms in organisms from bacteria to animals. These periodic rhythms result from a complex interplay among clock components that are specific to the organism, but share molecular mechanisms across kingdoms. A full understanding of these processes requires detailed knowledge, not only of the biochemical properties of clock proteins and their interactions, but also of the three-dimensional structure of clockwork components. Posttranslational modifications and protein–protein interactions have become a recent focus, in particular the complex interactions mediated by the phosphorylation of clock proteins and the formation of multimeric protein complexes that regulate clock genes at transcriptional and translational levels. This review covers the structural aspects of circadian oscillators, and serves as a primer for this exciting realm of structural biology.

## Overview of various circadian systems

**Fig. 1:**
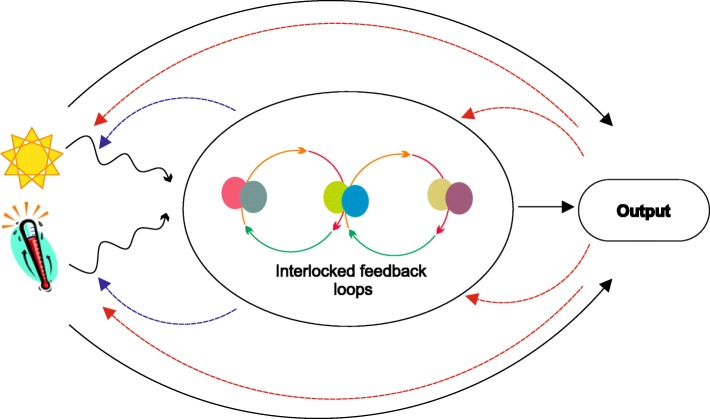
Generic model of the circadian clock. The complex network of coupled multiple feedback oscillators are represented by *solid color lines* and *ovals*. Clock genes forming a functional oscillator regulate the input and output pathways (*blue dashed lines*). Feedback from output pathways can also regulate the oscillator and the input pathways (*red dashed lines*). In addition to external input signal transduction for clock entrainment, input pathways can also directly affect clock output and vice versa (*solid black line*). The model is adapted from a model depicted in [3]

A circadian clock (CC) is an endogenous, self-sustaining, time-keeping system. Circadian clocks exist in most examined biological life forms, ranging from unicellular bacteria to highly complex higher organisms, including humans [1–3]. These clocks predict daily changes in the environment and regulate various physiological and metabolic processes [4, 5]. Clock genes across the kingdoms show limited conservation; nonetheless, the basic regulatory and time-keeping mechanism appears to be similar. CCs have an intrinsic period length of approximately 24 hours under constant conditions. Environmental cues, such as light and temperature, act as *zeitgebers* (time givers) that can reset the clock and also affect the rhythmic amplitude of clock outputs [4, 6, 7]. The process by which the clock is reset in response to day–night environmental changes is called entrainment. This synchronization is necessary because of variation in sunrise and sunset, as well as gradual retardation of Earth’s revolution periodicity, which necessitates responding to both seasonal and evolutionary timescales. Circadian rhythms are also temperature-compensated such that they can occur within a similar period over a wide range of biologically relevant temperatures [8–10]. Clocks in diverse organisms can be cell autonomous. For example, robust circadian rhythms of transcription have been observed in the single cells of *Cyanobacteria* and isolated mammalian fibroblasts, with minimal synchronization between the adjacent cells [11–13]. An oversimplified basic circadian network can be defined as consisting of three elements: input pathways that perceive and transmit signals that synchronize the clock to the environment, a central oscillator, and output pathways that link the oscillator to various biological processes. However, with the addition of new components to the clock network, our models of the circadian system are increasingly complex (Fig. 1). A given circadian oscillator consists of an autoregulatory network of multiple transcriptional and translational feedback loops, where the clock genes are activated or repressed by the rhythmic cycling of the proteins encoded by them. The input pathways themselves can also be rhythmically regulated by the circadian clock outputs [2–4, 14–17]. Together, the linear concept from input to clock outputs is actually an interwoven system of feedbacks.

CCs are well studied in prokaryotes (cyanobacteria) and eukaryotes (fungi, plants, insects, and mammals). In cyanobacteria, transcriptome expression of almost the entire genome is under circadian control [18, 19]. In fungal species, asexual spore formation, metabolism and stress responses, as well as other physiological [14, 20] and developmental processes [21, 22], show circadian rhythms. In humans, many physiological and behavioral processes, such as the sleep–wake cycle, body temperature, blood pressure, hormone production, and the immune system, are regulated in a circadian manner [23–25]. In plants, leaf and stomatal movement, hypocotyl elongation, hormonal signaling, and the expression of a large number of genes show circadian rhythms [26–29]. The circadian regulation of these physiological and developmental processes is ultimately a consequence of oscillating biochemical activities in each cell type. A circadian clock, to put it simply, is formed by a system of oscillating reactions.

Another characteristic feature of the circadian networks across life is the existence of multiple oscillators that coordinate differentially [30, 31]. This has introduced the concept of “pacemaker” and “slave” oscillators, wherein the pacemaker is the central oscillator that entrains to the external environmental cues and regulates the rhythmic output directly and/or by synchronizing slave oscillators, which then regulate given outputs. The slave oscillators are entrained by the central oscillator and may not exhibit all the circadian characteristics of a central oscillator. Multiple oscillators have been observed in cyanobacteria and *Neurospora crassa*. A self-sustained circadian oscillator composed of cyanobacterial core clock components has been reconstituted in vitro. In cyanobacteria, this suggests that a biochemical oscillator acts as a pacemaker and that a transcriptional–translational feedback loop (TTFL) is not important for driving circadian rhythms. However, circadian expression of genes was observed even when the biochemical oscillator was disrupted, suggesting that these two oscillators exist independently. When coupled to the biochemical pacemaker, the TTFL contributed to the robustness of the circadian clock [1, 32]. It has been proposed that these could be widespread in circadian-containing organisms, as a non-transcriptional oscillator is present in all three kingdoms of life [33].

Multicellular organisms have a complex architecture that consists of multiple cellular layers, tissues, and organs. In mammals, a hierarchical system of multiple circadian oscillators exists. The central pacemaker that is directly entrained by the external environmental cues is located in the suprachiasmatic nucleus (SCN) of the hypothalamus and synchronizes the peripheral clocks present throughout the organism. Transplantation of the fetal SCN into SCN-lesioned rats restored rhythmicity in a manner characteristic of the donor [34, 35]. The peripheral oscillators have clock components and properties similar to the pacemaker; however, they affect only the respective tissue or organ. Circadian rhythms of luciferase (LUC) expression were dampened after a few cycles in the non-SCN tissue culture from transgenic rat lines in which LUC was under the control of clock gene *Period 1* (Per1) promoter, but continued to show robust rhythms for many weeks in the cultured SCN tissue [36]. The rhythms of the peripheral oscillators are phase-delayed by 4–12 hours and less rapidly entrained as compared to the pacemaker, indicating that the SCN pacemaker is required to synchronize the self-sustained peripheral oscillators and that the signals for synchronization take some time, as suggested by the phase delay [36, 37]. Unlike mammals, studies suggest that the circadian network in *Drosophila* consists of multiple self-sustained, cell autonomous circadian oscillators with a pacemaker function in most of the cells. Isolated tissues from head, thorax, and abdomen exhibited a functional circadian oscillator that could be entrained by light [38]. Interestingly, rhythms for eclosion [39] and locomotor activity are driven by circadian oscillators placed in the brain. Studies indicate that oscillator neurons in the brain are coupled and communicate via Pigment-dispersing factor to drive the locomotor activity under constant conditions (constant light (LL) and constant darkness (DD)) [40–43]. Thus, the possibility of coupled oscillators driving circadian rhythms is very probable.

**Fig. 2:**
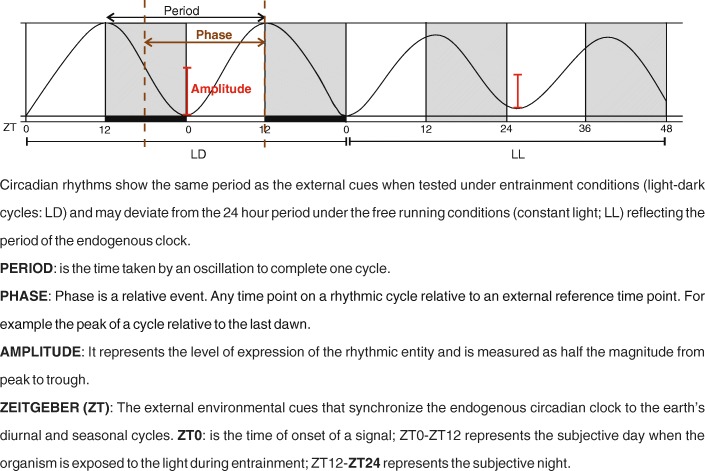
Box 1

Circadian rhythms can be represented as sinusoidal waves and are mathematically described by period, phase, and amplitude (Fig. 2). Entrainment by environmental cues (light and temperature stimuli) results in phase shifts. The phase can be delayed, advanced, or unchanged, depending on the time of the subjective day/night at which the stimulus is applied. If the stimulus appears in the early subjective night, the rhythm is delayed, whereas if given later in the subjective night, the rhythm is advanced. During the middle of subjective day/night, time points with little or no phase shift occur, and these are called "dead zones". Phase response curves demonstrate the transient phase shifts in the oscillation induced by a brief stimulus under constant conditions, as a function of the phase at which they are applied, and they are the best way to study entrainment in an organism by *zeitgebers*. The amplitude and the duration of the advances or delays are species-specific [44, 45].

The clockwork operates by the actions of positive and negative regulatory elements that form a complex network of multiple interlocked transcriptional and translational feedback loops that are self-sustained with robust and tunable molecular oscillators [3, 13, 14, 16, 17, 46, 47]. Recent work emphasizes the importance of post-translation regulation on the stability and functionality of clock components and, hence, circadian timing. Hetero- and homo-oligomerization and nuclear shuttling of the core-clock proteins are common features shared across the kingdoms. Sequential phosphorylation plays an important role in the stability of the oligomeric states, subcellular localization and, hence, the transcriptional activity of the clock proteins during the course of the day [48–52]. It is likely that formation of transient complexes, which form and reform relatively easily, is essential for accurate functioning of the CC. Eukaryotic clocks are therefore a complex system of transcriptional/translational regulators and kinases/phosphatases. A complete understanding of the molecular mechanisms of such clockworks requires a full structural characterization of the clock components and their complexes, which leads to hypothesis-driven understanding of the biochemical basis of cellular clocks. The structural aspects of CC regulation are relatively poorly understood in eukaryotes, but well defined for the cyanobacterial clock [1, 32]. This review summarizes the ongoing efforts to understand the function and physical interactions of the CC components, with special emphasis on structural aspects.

## The cyanobacterial circadian clock

**Fig. 3:**
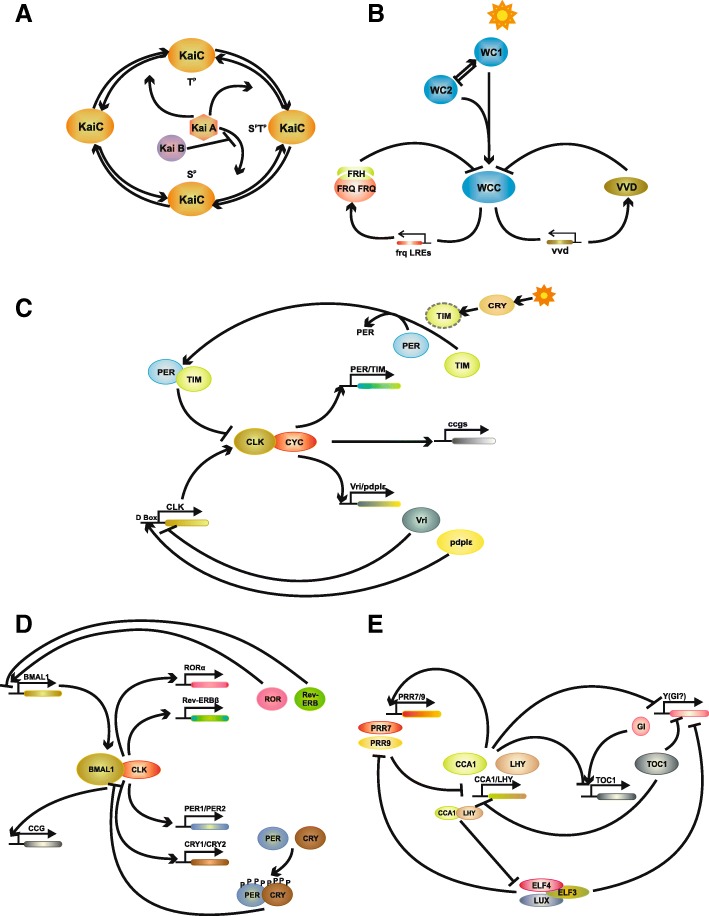
A simple schematic representation of the circadian clock in **a** cyanobacteria, **b** fungi, **c** insects, **d** mammals, and **e** plants

The cyanobacterial CC has been studied extensively using *Synechococcus elongates* (*Se*) as the model organism. Three proteins form the core oscillator: KaiC, KaiA, and KaiB (Fig. 3a). Their circadian rhythms are driven by a transcription- and translation-based autoregulatory loop of *KaiBC* gene expression, wherein KaiA and KaiC act, respectively, as positive and negative regulators of *KaiBC* gene expression [53]. A fully functional, temperature-compensated clock with an approximately 24-hour periodicity could be reconstituted in vitro with KaiA, KaiB, KaiC, and ATP [54]. Also, KaiC phosphorylation was found to be rhythmic in *S. elongatus* in continuous dark conditions in the absence of transcription and translation [55], suggesting that post-translational KaiC phosphorylation is central to Kai protein-based timekeeping. Further research revealed that the transcription/translation-based loop, though not a requisite for maintaining circadian rhythms in prokaryotes, is still important. Circadian gene expression has been observed in the absence of KaiC phosphorylation cycles. However, over shorter periods, *KaiBC* gene expression and accumulation of KaiB and KaiC proteins were observed to be rhythmic and temperature-compensated in the KaiA-overexpressing strain that forces constitutive KaiC phosphorylation. Dampened rhythms over a longer period were observed in KaiC mutant strains that were phospholocked or KaiC mutants that lacked autokinase activity, thus leaving KaiC unphosphorylated. These observations demonstrate that two pathways are important for the regulation of circadian rhythms: KaiC phosphorylation and the transcription/translation-based KaiC abundance cycle. The period and amplitude of the transcription/translation cyclic rhythms were modified in the absence of the KaiC phosphorylation cycle, and rhythms at low temperature were observed only when both oscillatory pathways are intact [56], suggesting that multiple coupled oscillatory systems are important for a robust and precise circadian clock in cyanobacteria. The mechanisms that control these two pathways are still unclear [1, 32].

**Fig. 4:**
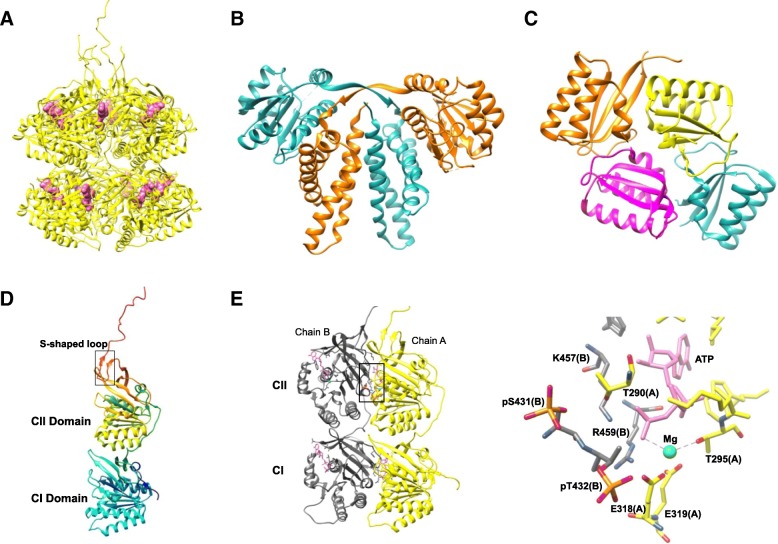
Crystal structures of cyanobacterial clock proteins KaiC, KaiA, and KaiB. **a** Side view of the KaiC hexamer (PDB 2GBL) with 12 ATP (*magenta*) binding sites. **b** KaiA dimer (PDB 1R8J). **c** KaiB tetramer (PDB 1WWJ). **d** KaiC monomer with CI and CII domains and the S-shaped loop. **e** Structure showing two chains (A and B) of the hexameric KaiC depicting the key phosphorylation site—Ser431 and Thr432—and the bound ATP at the subunit–subunit interface. On the right is a detailed view of the interface showing the glutamates close to ATP that help to activate phosphorylation

Structural studies have guided the understanding of the cyanobacterial clock components with an insight into their interactions that promote conformational changes and phosphorylation events vital for a functional clock. The atomic structures for KaiC, KaiA, and KaiB (Fig. 4) and/or their domains have been determined using X-ray crystallography, NMR spectroscopy, and electron microscopy [57–60]. KaiC from *S. elongatus* was shown to be a double-doughnut-shaped hexamer with 12 ATP binding sites between the N-terminal KaiC I and the C-terminal KaiC II rings (Fig. 4a) [58]. The *S. elongatus* KaiA protein forms a 3D domain-swapped dimer (Fig. 4b). It has three domains: an N-terminal domain (residues 1–129), a linker (130–179), and a C-terminal dimerization domain (180–283) [60]. The C-terminal domain forms a four-helix bundle, which has been confirmed by the structures of the C-terminal domain of KaiA from *Anabaena* sp. PCC7120 [59] and from *Thermosynechococcous elongates* (*Te*) BP-1 [61, 62]. The crystal structure of KaiB (Fig. 4c) revealed a protein with a thioredoxin-like fold [59, 63, 64], which forms dimers and tetramers [63].

*KaiC is a kinase/phosphatase/ATPase:* The principal clock component, KaiC, is the only protein with known enzymatic activity in the cyanobacterial clock. It acts as an autokinase, an autophosphatase, and an ATPase, exhibiting these functions both in vitro and in vivo [65–67]. The crystal structure of full-length KaiC revealed a homologous two-domain fold, resulting from gene duplication, in the monomer, with N-terminal CI and C-terminal CII domains (Fig. 4d). The C-terminal tail of CI links the two domains, whereas the C-terminal tail of CII protrudes out of the domain region, following an S-shaped loop on the periphery of the hexamer [58].

*KaiC functions as kinase/phosphatase:* The key phosphorylation sites identified in the KaiC CII domain are Ser431 and Thr432. Phosphorylation at these sites occurs at the subunit–subunit interface (Fig. 4e), where they are close to an ATP molecule bound to an adjacent subunit [68, 69]. The cycle of KaiC phosphorylation during the day, as well as hypophosphorylation at night, over a ~ 24-hour period proceeds in four steps: from Ser431 and Thr432 KaiC (ST unphosphorylated) to Thr432 phosphorylation (SpT), Ser431 phosphorylation (pSpT), Thr432 dephosphorylation (pST) and Ser431 dephosphorylation (ST). The reaction at each step is regulated by the product from the preceding step [70, 71]. Thr432, as the site to be phosphorylated first, is consistent with the crystal structure of KaiC, where all Thr432 residues in the six subunits are phosphorylated, in contrast to only four (out of six) Ser431 phosphorylations. Thr432 phosphorylation, which leads to new contacts across the subunit interface, enhances Ser431 phosphorylation [68, 69]. Complete phosphorylation of both Thr432 and Ser431 converts KaiC from an autokinase to an autophosphatase. Thus, the KaiC phosphorylation cycle determines KaiC enzymatic activity [67]. A third phosphorylation site was found at Thr426 that forms a hydrogen bond with the phosphate group of pSer431. T426A, T426E, and T426N mutants were observed to be arrhythmic. Thr426 was also observed to be phosphorylated in the crystal structures of S431A and T432E/S431A KaiC mutants [68, 72, 73]. In summary, the phosphorylation state of these key residues governs the functionality of the KaiC protein.

*KaiC ATPase activity:* KaiC shows extremely weak but stable ATPase activity (~ 15 ATP per KaiC per phosphorylation cycle) [53]. There are two ATPase activity regions in KaiC: i) slow KaiC CI ATPase activity that plays a role in time delay and the conformational switch needed for KaiC–KaiB interaction [74–76]; and ii) in the CII domain of KaiC catalyzing phosphorylation/dephosphorylation activity [76–78]. Work by Terauchi et al. [76] shows that ATPase activity displays circadian rhythms in the presence of KaiA and KaiB. KaiC variants mimicking the dephosphorylated and doubly phosphorylated state influenced its ATPase activity (non-phosphorylated state, more active; fully phosphorylated state, less active), suggesting both the kinase and the ATPase activity are closely linked. The mutants exhibiting long and short period displayed a linear correlation between the ATP hydrolysis and the circadian frequency. Temperature compensation is intrinsic to the ATPase activity. The ATPase activity showed strong temperature compensations in KaiC-only incubations and was only slightly affected in the presence of KaiA and KaiB in the temperature range 25–35°C. Terauchi et al. [76] proposed the ATPase activity of KaiC to be the most basic molecular mechanism that governs the period of a cyanobacterial circadian clock and is temperature compensated.

Analysis of the crystal structures of wild-type KaiC (4TL8) and its period-modulating variants in the pre- and post-hydrolysis states (PDB entries 4TL9 and 4TLA) revealed two structural bases of slow KaiC CI ATPase activity [79]. First, the hydrogen bonding of the lytic water moiety with the carbonyl oxygen of F199, the nitrogen of the side chain of R226 of KaiC, and another water molecule creates a steric hindrance, positions it farther, thus making it inaccessible to the γ-phosphate of the ATP (refer to the figures in [79]). Second, the slow *cis*-*trans* isomerization of a peptide (D145–S146) accompanying the ATP hydrolysis (PDB entries 4TL9, 4TLC, and 4TLA; refer to the figures in [79]) results in a substantial increase in the energy barrier to overcome, in order to disrupt the γ-phosphate–O bond of the ATP. CI and CII ATPases together form a coupled CI–CII ATPase system that is driven predominantly by the slow CI ATPase [79].

Crystal structures of KaiC (PDB 3DVL) and KaiC mutants (3JZM, 3K0A, 3K09, 3K0E, 3K0F, and 3K0C) [73] reveal that the ATP molecules bound between two subunits are recognized differently in the two subunits. The ATP phosphates are in close proximity to two glutamates in CII and are coordinated with Mg^2+^ (Fig. 4e). The glutamate close to the γ-phosphate (γ-P) group is also observed to be close to Thr432 and may therefore act as a general base for the hydrolysis and proton abstraction from Thr432 and Ser431 that help activate phosphorylation. The resulting γ-P transfer might increase the interaction between the subunits, thus forming a more compact hyperphosphorylated KaiC, as also observed in small-angle X-ray scattering (SAXS) measurements of the KaiC mutants mimicking various phosphorylation states [80]. Thr432/Ser431/Thr426 in CII corresponds to Glu198/Glu197/Asp192 in CI. X-ray crystallography, mass spectrometry, and KaiC T432E/S43E1 mutations showed no phosphorylation in CI, suggesting that ATP hydrolysis in CI generates the energy required for the enzymatic activity in the CII domain, rather than phosphoryl transfer [68, 69, 73, 79].

*Kai protein interactions and the phosphorylation cycle:* Both in vitro and in vivo, KaiA is an enhancer of KaiC phosphorylation, while KaiB antagonizes the action of KaiA [66, 67, 81, 82]. Structural and biophysical studies using various biochemical, spectroscopic, and crystallographic methods have helped to understand the KaiAC and KaiBC complexes and provided insight into the interaction of KaiA and KaiB with KaiC. KaiA binds through its C-terminal domain to the KaiC C-terminal tail at two interfaces: CIIABD peptide and the ATP binding pocket [62, 83]. KaiA contains an amino terminal pseudodomain that is proposed to receive environmental cues transmitted to potentiate entrainment [66, 67, 81, 82, 84]. KaiB interacts with the pSer431:Thr432-KaiC phosphoforms that inactivate KaiA in the KaiABC complex [68, 69]. The balance between the two activities is modulated by an “A-loop” switch (residues 488–497) in the C-terminal tail of the KaiC CII domain. KaiA stabilizes the exposed A-loops and stimulates KaiC autokinase activity, while KaiB prevents KaiA interaction with the loops, thereby stabilizing the internal core structure and, hence, locking the switch in the autophosphatase phase. A dynamic equilibrium between the buried and exposed states of the loops determines the levels of KaiC phosphorylation. It was hypothesized that binding of KaiA might disrupt the loop fold of a single unit that is engaged in the hydrogen bonding network across the subunits at the periphery [58], resulting in a weakened interface between the adjacent CII domains. This would lead to conformational changes within the CII ring that support serine/threonine phosphorylation. Initially, ATP is too distant from the phosphorylation sites to affect a phosphoryl transfer reaction; however, changes within the CII ring might relocate the bound ATP closer to the phosphorylation sites and/or enhance the retention time of ATP by sealing the ATP binding cleft [83, 84]. In contrast, KaiB interacts with the phosphoform of the KaiC hexamer. These structural analyses support the hypothesis that KaiA and KaiB act as regulators of the central KaiC protein.

Structural studies [75, 85] provide a detailed analysis to explain how these protein–protein interactions among KaiC, KaiA, and KaiB and their cooperative assembly alter the dynamics of rhythmic phosphorylation/dephosphorylation, in addition to ATP hydrolytic activity of KaiC, generating output that regulates the metabolic activities of the cell. An earlier spectroscopic study [86] proposed a model for the KaiC autokinase-to-autophosphatase switch, which suggests that rhythmic KaiC phosphorylation/dephosphorylation is an example of dynamics-driven allostery that is controlled mainly by the flexibility of the CII ring of KaiC. Using various KaiC CII domain phosphomimetics that mimic the various KaiC phosphorylation states, the authors observed that in the presence of KaiA and KaiB, different dynamic states of the CII ring followed the pattern ST_flexible_ → SpT_flexible_ → pSpT_rigid_ → pST_very-rigid_ → ST_flexible_. KaiA interaction with exposed A-loops of the flexible KaiC CII ring activates KaiC autokinase activity. KaiC hyperphosphorylation at S431 changes the flexible CII ring to a rigid state that allows a stable complex formation between KaiB and KaiC. The resulting conformational change in KaiB exposes a KaiA binding site that tightens the binding between KaiB and the KaiA linker, thus sequestering KaiA from A-loops in a stable KaiCB(A) complex and activating the autophosphatase activity of KaiC [86]. KaiB binding and dephosphorylation are accompanied by an exchange of KaiC subunits, a mechanism that is crucial for maintaining a stable oscillator [1].

KaiB is the only known clock protein that is a member of a rare category of proteins called the metamorphic proteins [87, 88]. These can switch reversibly between distinct folds under native conditions. The two states in which KaiB exists are: the ground state KaiB (gsKaiB; Fig. 4c) and a rare active state called the fold switch state KaiB (fsKaiB) [88]. Chang et al. [88] showed that it is the fsKaiB that binds the phosphorylated KaiC, thus sequestering KaiA and starting KaiC dephosphorylation. Hence, the previously known crystal structures of KaiB are of gsKaiB.

**Fig. 5:**
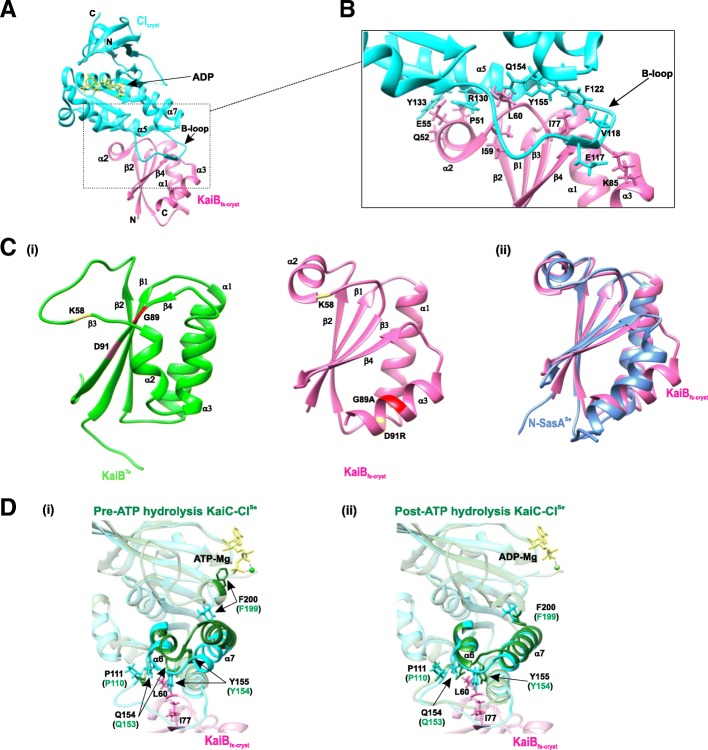
Rare active fold-switched form of KaiB (fsKaiB) binds to the post-hydrolysis state of KaiC CI domain. **a** A 1.8-Å resolution structure of the KaiB_fs-cryst_–CI_cryst_ complex (PDB 5JWO; from *T. elongates*). The ribbon diagram shows KaiB_fs-cryst_ in *pink*, CI_cryst_ in *cyan*, and bound ADP in *yellow*. *Enclosed dotted box* depicts the binding interface between KaiB_fs-cryst_ and CI_cryst_. **b** Enlarged view of the KaiB_fs-cryst_–CI_cryst_ complex binding interface depicting the interacting residues. **c** Structural comparison of KaiB ground state (gs) and fsKaiB: (i) KaiB^Te^ (gsKaiB; PDB 2QKE, subunit A) in *green*, KaiB_fs-cryst_ in *pink*; (ii) superposition of KaiB_fs-cryst_ in *pink* with N-SasA^Se^ (PDB 1T4Y; Se: *S. elongatus*) in *cornflower blue*. Residues K58, G89, and D91 are highlighted in *yellow*, *red*, and *orange*, respectively. **d** Comparison of the ATP binding site of the KaiB_fs-cryst_–CI_cryst_ complex with ATP binding site of KaiC CI structures (from *S. elongates*) in the pre- and post-hydrolysis states: superposition of ADP-bound CI_cryst_ (*cyan*) with the CI^Se^ structure (*green*) in the (i) pre-ATP hydrolysis state (PDB 4TLC, subunit C) and (ii) post-ATP hydrolysis state (PDB 4TLA, subunit E)

A high-resolution (1.8 Å; Fig. 5a, b) structure of KaiB_fs-cryst_ (fsKaiB mutant: KaiB_fs_ [G89A and D91R], partially truncated at the C-terminus) and CI_cryst_ (truncation at the N-terminus of the isolated CI domain of the KaiC monomer) complex (PDB 5JWO) shows an interface that primarily consists of the residues from the fold-switched C-terminal half of KaiB and the B loop of the CI_crys_ [75]. KaiB in its fold switch state adopts a thioredoxin-like fold similar to that in the N-terminus of SasA that binds KaiC (Fig. 5c) [88, 89]. Previous deletion and substitution mutation studies of the KaiC B-loop show an absence of or weakened interaction between KaiB and KaiC and between SasA and KaiC. Binding of fsKaiB inhibits the interaction between SasA and KaiC as both SasA and fsKaiB compete for the same binding site on the KaiC CI domain [88, 90]. fsKaiB interaction with KaiC sequesters KaiA, thus switching a fully phosphorylated KaiC from a kinase to the phosphatase and commencing a phase transition. The same rare active state KaiB (fsKaiB), in complex with KaiC, interacts with CiKA, which then dephosphorylates RpaA (discussed later in the “Light: input to the clock” section), thus regulating the expression of class 1 (repressing) and class 2 (activating) genes.

**Fig. 6:**
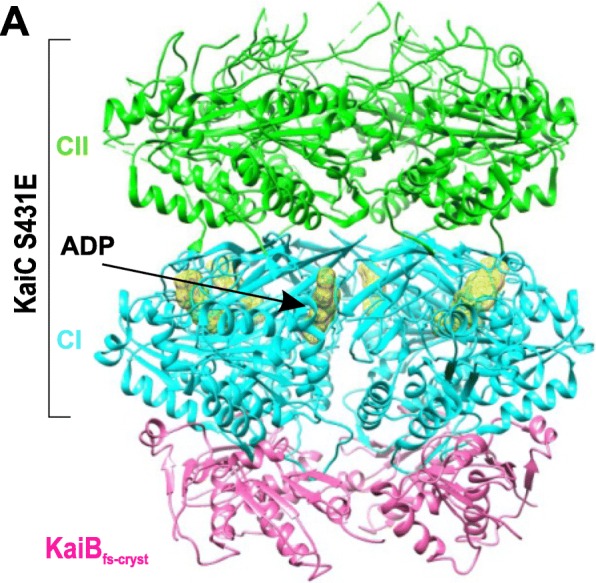
Kai clock protein complex assembly. **a** A 3.87-Å structure of KaiB_fs-cryst*_and KaiC S431E complex hexamer (PDB 5JWQ) with KaiB_fs-cryst*_ in *hot pink*, the KaiC CI domain ring in *cyan*, CII in *green*, and ADP densities in *yellow*

ATP hydrolysis in the KaiC CI ring is a pre-requisite for KaiC interaction with fsKaiB [74, 91]. A comparison (Fig. 5d) of a high-resolution (1.8Å) crystal structure of the KaiB_fs-cryst_–CI_cryst_ complex bound to ADP [75] with the structures of the KaiC CI domain (from *S. elongates*) in pre- and post-hydrolysis states displayed large conformational changes in the KaiC CI domain at the ATP binding site after ATP hydrolysis. Residue F200, near the ATP binding site and the α6 and α7 helices, moves “downward” as a result. Residues Q154 and Y155 of α6 then constitute the KaiB_fs-cryst_–CI_cryst_ interface. Another 3.87Å resolution crystal structure (Fig. 6) of the KaiB_fs-cryst*_ (KaiB_fs-cryst_ variant with I88A substitution)–phosphomimmic KaiC S431E complex hexamer, crystallized in the presence of ATP, showed densities of ADP between each CI subunit [75] as opposed to previous crystals of KaiC and its mutant captured in the pre-hydrolysis state [92]. The structure also shows conformational changes at α6 and α7 helices of KaiC CI that accompany ATP hydrolysis. These analyses reveal that the energy provided by the ATP hydrolysis results in a much-needed conformational switch of the KaiC CI domain that captures fsKaiB [75].

Dynamic structural analysis of Kai CI ring tryptophan mutants using fluorescence spectroscopy demonstrated a link between slow ATP hydrolysis and the KaiC CI binding to KaiB. The structural change triggered by slow ATP hydrolysis results in a structural rearrangement in the CI ring at the inner hexamer radius side (includes α7) and the D145–S146 peptide, without altering the overall hexameric framework of the KaiC CI ring. A slow KaiC CI ring conformational change (from pre- to post-hydrolysis state) coupled with the phosphorylation of KaiC results in a KaiC conformation that is receptive to the incoming active KaiB. This conformational switch in KaiC, coupled with ATPase activity and KaiC phosphorylation state, signals KaiC–active KaiB complex assembly and provides an explanation for the slowness of the cyanobacterial clock [91].

**Fig. 7:**
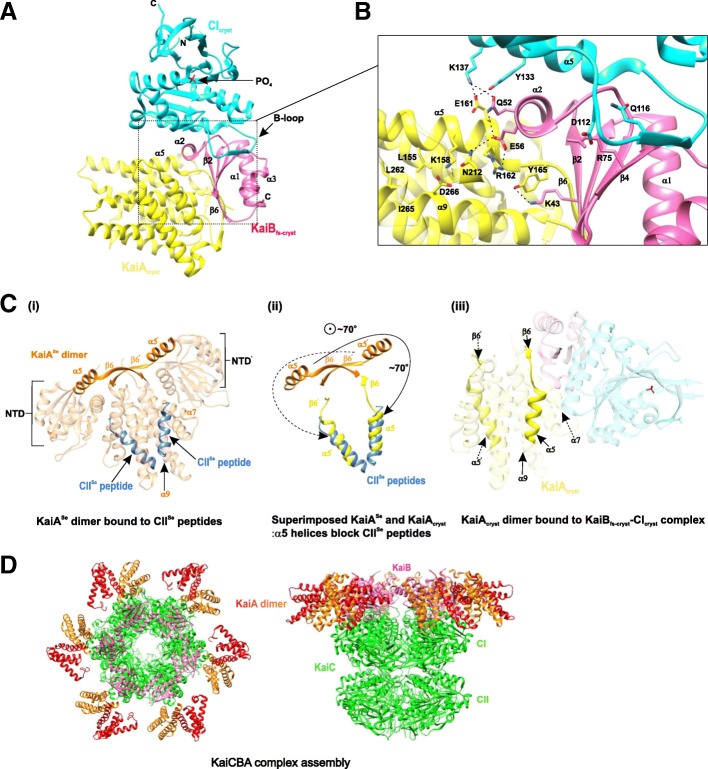
KaiCBA ternary complex depicting the KaiA autoinhibition mechanism. **a** A 2.6-Å ternary complex between KaiA_cryst_ and KaiB_fs-cryst_–CI_cryst_ (PDB 5JWR; KaiA_cryst_ in *yellow*, KaiB_fs-cryst_ in *pink*, and CI_cryst_ in *cyan*). **b** Enlarged view of the *enclosed box* in **a** depicting the binding interface of the ternary complex. *Dashed lines* show the electrostatic interactions. **c** Conformational changes in the KaiA dimer when sequestered into a KaiCBA complex. (i) Structure of KaiA in *orange* bound to CII peptides in *blue* (from *S. elongates*; PDB 5C5E) highlighting the α5 and α5^’^ helices and β6 and β6^’^ strands of the two KaiA monomers. (ii) KaiA^Se^ (*orange*) and KaiA_cryst_ in ternary complex (*yellow*) superimposed showing only the α5 and α5^’^ helices and β6 and β6^’^ strands. (iii) The CI_cryst_–KaiB_fs-cryst_–KaiA_cryst_ ternary complex. Panels (i), (ii), and (iii) highlight only the α5 and α5^’^ helices and β6 and β6^’^ strands of the two KaiA monomers depicting the structural basis of the mechanism of KaiA autoinhibition. **d** Top and side views of higher KaiCBA complex assembly (PDB 5N8Y) depicting the KaiC hexamer in *green*, the hexameric ring of KaiB monomers in *pink*, and KaiA homodimers in *red* and *orange*

A 2.6Å crystal structure (Fig. 7a) of the ternary complex of KaiA_cryst_ (KaiA_ΔN_–C272S: KaiA_ΔN_ is KaiA variant missing the N-terminus; PDB 5JWR) in complex with KaiB_fs-cryst_–CI_cryst_ provides the molecular level understanding of the co-operative assembly of the Kai components and the regulation of output signaling pathways by the Kai oscillator. Ternary complex analysis indicates that the presence of KaiA results in an increase in the affinity of KaiB for KaiC CI domain (Fig. 7b) as indicated by electrostatic interactions that form a triple junction between CI_cryst_, KaiB_fs-cryst_, and KaiA_cryst_ and an increase in the number of hydrogen bonds and the interfacial surface area between KaiB_fs-cryst_–CI_cryst_ [75]. Thus, KaiA drives the cooperative assembly of KaiB–KaiC. KaiA-activated KaiC phosphorylation drives the tightening of the CII ring, stacking CI over CII. Additionally, it is observed that the enhanced interaction between the CI and CII domains, as a result of CII rigidity, in turn suppresses KaiC ATPase activity [86].

Analysis of the ternary complex also reflects on the auto-inhibitory role of KaiA (Fig 7c). Bound KaiA_cryst_ dimer in the ternary complex shows large conformational changes compared to the KaiA structure from *S. elongates*. β6 strands of KaiA_cryst_ monomers rotate by 70° and β6 of one monomer forms an antiparallel β-sheet by docking onto β2 of KaiB_fs-cryst_. This rotates the α5 helices of both KaiA_cryst_ monomers downwards onto α7 and α9 (the KaiC binding site) at the KaiA_cryst_ dimer interface and blocks it. Thus, KaiB binding to KaiA induces changes in KaiA conformation and, as a result, KaiA inhibits itself from binding to KaiC. Structure-guided mutagenesis of the α5 helix and α7 and α9 helices of KaiA weakened ternary complex formation. Mutations in the β2 strand of fsKaiB disrupted the antiparallel β-sheet formation, eliminating the interaction between KaiA_ΔN_ and fsKaiB–KaiC CI complex. The mutation did not affect complex formation between fsKaiB and KaiC CI. The analogous mutations in *kaiB*^*Se*^ disrupted the circadian rhythms in vivo [75].

In a parallel study [85], when Kai proteins were incubated in an excess of MgATP at 30°C, Snijder et al. observed that multiple stoichiometries of the phosphorylation-dependent Kai protein complex assemble simultaneously over a period of 24 hours. Initial formation of KaiCA complexes with autophosphorylation activity drives the cooperative assembly of phosphorylated KaiCB complexes (C_6_B_1_, C_6_B_2_ …. C_6_B_6_) followed by the formation of higher order KaiCBA complexes (C_6_B_6_A_2_ …. C_6_B_6_A_6_) that peaks in 12 hours, followed by the dephosphorylation phase wherein the KaiCBA complex disassembly is not the reverse of complex assembly. Incubation at 4°C favored autophosphorylation with KaiCBA complex levels increasing even after 24 hours. A protocol devised on these observations is used to obtain Kai complex assemblies "frozen" in various states for structural analysis. KaiCBA complex assembly could be obtained with near complete occupancy of the KaiA binding site by prolonged incubation of KaiC, B, and A in 1:3:3 molar ratio. Structural maps of KaiC_6_B_6_A_12_ and KaiC_6_B_6_ complex assemblies obtained at 4.7Å and 7Å resolution using mass spectrometry and single particle cryo-electron microscopy (EM) and fitted with previous crystal structures of the individual Kai proteins reveal that KaiCB assembly consists of three stacked rings of which the bottom two correspond to KaiC, and KaiB forms the top ring (Fig. 7d). The KaiB ring sits on top of KaiC CI [85]. Consistent with the previous study [88], analysis of KaiCBA complex cryo-EM maps indicates that KaiC-bound KaiB in the KaiCBA complex is fsKaiB. Also, it is the KaiBC complex assembly that guides the formation of higher KaiCBA assemblies [85].

Analysis of KaiCBA using the KaiA dimer crystal structure confirms the participation of KaiA as dimer in the formation of Kai complex assemblies. KaiB interacts with KaiA through its β2 strand and the binding is asymmetric, suggesting involvement of only one KaiB monomer in binding. Structure-guided mutagenesis of KaiC Ala106 and KaiB Lys42 and native mass spectrometry indicated their significance in KaiC–KaiB and KaiB–KaiA interactions, respectively [85]. KaiB Lys42 mutation in *S. elongates* and its analogus Lys43 mutation in *T. elongatus* disrupted clock rhythmicity in vivo [75].

Although KaiC’s autokinase and ATPase activities are fairly well characterized, KaiC dephosphorylation is less clear. The KaiC CII domain does not share the typical motif of the serine/threonine phosphatase family [93], but it does have a unique kinase/phosphatase activity at the subunit interface [78]. Egli and coworkers [78] hypothesized that this unique feature of KaiC is consistent with an unusual mechanism of dephosphorylation wherein ATP is regenerated from ADP in the CII half of KaiC, attributing a phosphoryl-transferase, rather than phosphatase, activity to KaiC. Also, Thr426 was observed to be phosphorylated in the T432E/S431E mutant crystal structure (PDB 3S1A) [66], supporting the hypothesis of phosphate transfer from ATP via a mechanism similar to Thr432 and Ser431 phosphorylation. The observation that KaiC does not appreciably consume ATP fits this model [78].

The three-dimensional structures of the clock components KaiA, KaiB, and KaiC are well defined and understood. Earlier studies of the complexes of these proteins using spectroscopic, computational, and hybrid structural approaches all support a likely mechanistic model resembling a switch from autokinase to phosphatase, or a possible autophosphotransferase activity of KaiC, and explain how it is related to KaiC ATPase activity. Further structural studies have made efforts to decipher the precise state underlying the switch; high-resolution crystal structures of KaiC–KaiB and KaiC–KaiB–KaiA complexes emphasize the importance of ATP hydrolysis of KaiC and conformational changes that trigger the assembly and disassembly of KaiC, B, and, A proteins. Kai components exist in a dynamic equilibrium between ground/inactive and the rare active state. The structures provide a molecular basis to the mechanism wherein ATP hydrolysis-induced conformational change in KaiC captures and stabilizes the interacting partner KaiB in the active state and simultaneously induces a switch between the varied enzymatic roles of KaiC that governs the phosphorylation/dephosphorylation cycles and regulates the circadian oscillator. Further studies of the KaiC–KaiA complex and the structures of the complexes that occur during the disassembly of the Kai complex are needed to understand the core circadian oscillator system and its regulation.

## Circadian clocks in eukaryotes

This section briefly summarizes the various models for known eukaryotic circadian clocks and provides insight into structural research in progress.

### The circadian clock in fungi

The *Neurospora crassa* circadian oscillator is arguably the best understood eukaryotic circadian system [31, 94, 95]. It has assisted in the elucidation of the concepts in eukaryotic clock mechanisms, yet many questions remain unanswered. With the limited structural knowledge of fungal clock proteins, the mechanism that underlies the functioning of core-clock components and posttranslational regulation is obscure. In the fungal CC (Fig. 3b), WHITE COLLAR 1 (WC-1), WHITECOLLAR 2 (WC-2), FREQUENCY (FRQ), and FRQ-INTERACTING RNA HELICASE (FRH) form crucial components of the clock. WC-1 and WC-2 are GATA-type zinc-finger DNA binding transcription factors that form the positive elements of the rhythmic loop [2, 47]. Together, they form a heterodimeric WHITE COLLAR COMPLEX (WCC) via their PER-ARNT-SIM (PAS) domains that bind to two light-responsive elements (LREs) of the *frq* promoter and activate the transcription of *frq*. In the late subjective night in constant darkness, heterodimeric WCC complex (D-WCC) binds to the distal LRE region of the *frq* promoter to activate *frq* transcription. *frq* mRNA levels peak in the early subjective morning and subsequently lead to FRQ accumulation that peaks in the late subjective day [2, 15, 96]. FRQ acts as the key negative element and is expressed in two isoforms: a long and a short form [10]. The two isoforms form a dimeric complex that interacts with WCC and inhibits *frq* transcription [15]. WCC-FRQ interaction is mediated by FRH [47, 97]. FRQ is simultaneously and progressively phosphorylated to release the repression on D-WCC and is degraded via a ubiquitin-proteasome-mediated pathway. FRQ also forms a positive loop, interlocked with the primary loop by positively regulating the expression of WC-1 [2, 98].

Among the core-clock components, WC-1 consists of three PAS domains: PAS-A, PAS-B, and PAS-C. Of the three PAS domains, PAS-A belongs to a specialized class of light, oxygen, or voltage (LOV) domain and functions as a blue-light photoreceptor. The function of PAS-B is unclear, and PAS-C is required for the interaction between WC-1 and WC-2 [99, 100]. WC-2 consists of a single PAS domain, important for interaction with WC-1, a coiled-coil domain with unknown function and a putative nuclear localization signal (NLS) [99, 101, 102]. FRQ is a phosphoprotein with a coiled-coil domain close to its N-terminus that mediates homodimerization. An NLS next to the coiled-coil domain of FRQ is essential for clock function [103]. The central and C-terminal part of FRQ is predicted to be largely unstructured and has no sequence similarity to any known protein domain [97, 104]. Apart from its role in the clock feedback loop, WC-1 is also a blue-light photoreceptor important for photomorphogenesis [2, 47, 96]. Light activation of WC-1 possibly results in the formation of a large WCC complex (L-WCC) that binds to the LREs, leading to the activation of transcription of the light-induced genes (*frq* and *vivid* (*vvd*) are two of them) [2, 101, 105–107]. VIVID (VVD) protein is another flavin-binding blue-light receptor in fungi that plays a role in phase regulation, entrainment, transient light responses, and temperature compensation in *Neurospora* circadian rhythms [2, 105, 106]. VVD and WC-1 are two LOV domain-containing photoreceptors that share sequence similarity in the core domain and bind FAD as the photosensory element [2]. The mechanism by which VVD inhibits nuclear WCC is unclear [2, 107]. Thus far, the LOV/PAS domain is the only recurring domain observed in the *Neurospora* clock. VVD is the only LOV domain containing a protein for which the crystal structure has been solved in the light and dark state, by Zoltowski et al. [106] (see below).

### Circadian clocks in insects and mammals

Identification and isolation of the first clock gene, *period* (*per*), in *Drosophila* and subsequent analysis of its expression led to the first molecular model of an animal circadian oscillator [108, 109]. The *Drosophila* and mammalian clock genes share a high level of sequence similarity and have orthologs. The primary feedback loop of the clock (Fig. 3c, d) consists of the positive elements CLOCK (dCLK) and CYCLE (CYC) in *Drosophila* and CLOCK and BMAL1 in mouse. These positive elements in *Drosophila* and mouse are members of the basic helix-loop-helix (bHLH)-PAS (Period-Arnt-Single-minded) transcription factor family, and they heterodimerize to activate the transcription of genes containing E-box *cis*-regulatory elements in their promoter region: *Period* (d*Per*) and *timeless (*d*Tim)* in *Drosophila* and period genes (*Per1*, *Per2*, and *Per3*) and cryptochrome genes (*Cry1* and *Cry2*) in mouse [46, 110–114]. mPER/mCRY (dPER/dTIM in *Drosophila*) proteins translocate to the nucleus and repress their own transcription by acting on CLOCK/BMAL1 (dCLK/dCYC) activity [17, 112, 115–119]. A putative homolog of d*Tim* is retained in mammals (m*Tim*); however, unlike a central role for d*Tim*, the function of m*Tim* in the mammalian circadian clock is not clear. An essential role similar to that of d*Tim* is performed by Cry genes in the mammalian circadian clock [120–122]. Interestingly, studies have shown that Cry genes, both in *Drosophila* and mammals, regulate the circadian clock in a light-dependent (photoreceptors) and a light-independent manner [112, 123–127]. However, their role as photoreceptors in mammals is still debated (discussed in the “Light: input to the clock” section).

The core-clock loop integrates with other regulatory systems that further fine-tune the mammalian clock system, wherein CLOCK/BMAL1 activates transcription of the members of the orphan nuclear receptors family (Rev-ErbA/NR1D (Nuclear receptor family 1 group D). Rev-erbα and β, and Retinoic acid receptor (RAR)-related orphan receptors (RORα, β, and γ)) in mammals via recognition of their E-box elements [128–131]. RORs and Rev-erbs, in turn, regulate the rhythmic expression of BMAL1 by alternatively binding to the retinoic-acid-related orphan receptor response elements (ROREs) on its promoter [132, 133]. RORs act as transcriptional activators of BMAL1 [129, 131, 132], whereas Rev-erbs act as repressors [128, 132]. Also, the genome-wide binding patterns of both Rev-erb α and Bmal1 showed regulatory regions that bind to most of the clock proteins and the proteins involved in various metabolic pathways, emphasizing the importance of Rev-erb/BMAL1 association with the circadian clock and metabolic functions (discussed later in the Rev-erb interactions section). Similarly, in *Drosophila*, dCLK/dCYC activates the transcription of *vrille* (*vri*) and *Par domain protein 1ɛ* (*Pdp1ɛ*) by binding to the VRI/PDP1-box (V/P) of the *clk* promoter to form the second loop [134, 135].

**Fig. 8:**
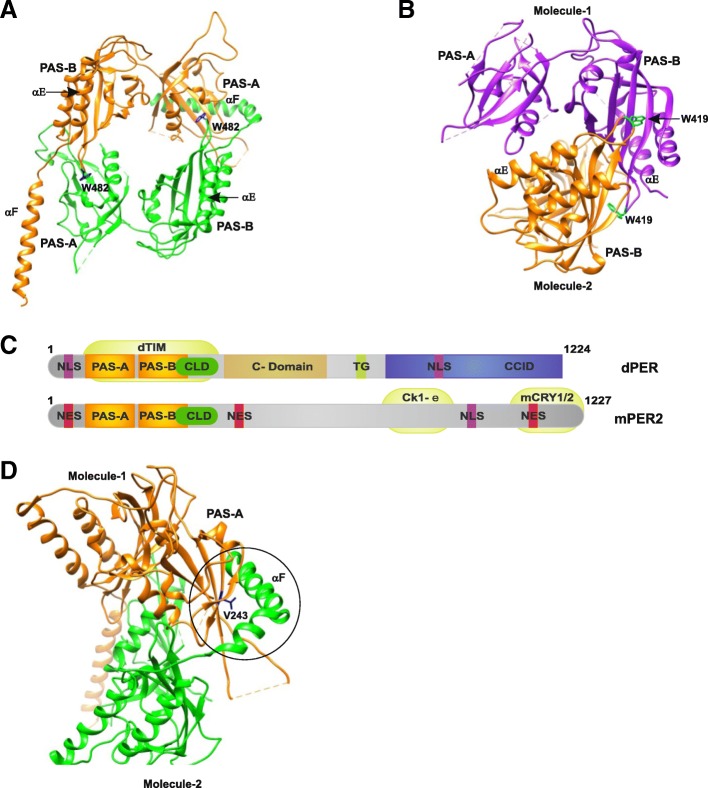
Crystal structures of the period proteins. **a** dPER (PDB 1WA9) and **b** mPER2 (PDB 3GDI) dimers in cartoon representation. The conserved Trp482 (dPER, *dark blue*) and Trp419 (mPER2, *cyan*) residues are shown in stick representation. **c** The domain architecture of dPER and mPER2 proteins. The two PAS domains (PAS-A and PAS-B), the cytoplasmic localization domain (CLD, *green*), the conserved C-domain (*light brown*), nuclear localization signals (NLS, *purple*), NES (*red*), the threonine-glycine (TG) repeat region, and the dCLK:CYC inhibition domain (CCID, *blue*) of dPER and/or mPER2 are shown. CKIe, mCRY1/2, and dTIM are shown at their binding sites. **d** dPER structure representing the PAS-A–αF interaction (*encircled region*) interface and depicting the location of V243 (*blue*)

**Fig. 9:**
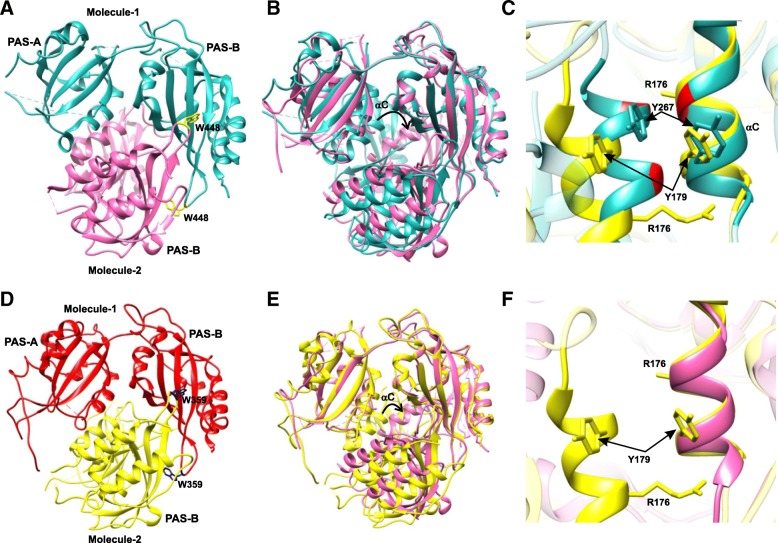
Crystal structures of mPER1 (PDB 4DJ2) and mPER3 (PDB 4DJ3) fragments. **a** Cartoon representation of mPER1 (residues 191–502). The conserved Trp448 (*yellow*) is shown in stick representation. **b** Comparison of the mPER1 (*cyan*) and mPER2 (*pink*) crystal structures. Movement of the PAS-A/αC helix of molecule 2 is indicated by a *black arrow*. **c** Closeup view of the structural comparison of the PAS-A/αC dimer interface of mPER1 (*cyan*) and mPER3 (*yellow*). Gly residues in mPER1 are shown in *red* and Arg residues in mPER3 are labeled. **d** Cartoon representation of mPER3 (108–411). The conserved Trp359 (*blue*) is shown in stick representation. **e** Comparison of the mPER3 (*yellow*) and mPER2 (*pink*) crystal structures. The *black arrow* indicates the location of movement of the PAS-A/αC helix of molecule 2. **f** Closeup view of the structural comparison of the PAS-A/αC dimer interface of mPER2 (*pink*) and mPER3 (*yellow*). PAS-A/αC dimer interaction is present in mPER1 and mPER3, but absent in mPER2, because of the different relative orientation of the monomers in (mPER2)_2_ compared to the mPER1 and mPER3 homologues

*The interactions of PERIOD proteins*: Crystal structures of fragments of *Drosophila* PERIOD (dPER residues 232–599) and mouse PERIOD (mPER1 residues 191–502, mPER2 residues 170–473, and mPER3 residues 108–411) proteins (Figs. 8 and 9) provide insights into the physical mechanism underlying circadian rhythm generation. The fragments include the two PAS domains (A and B), residues N-terminal to PAS-A, named the “N-terminal cap”, and the αE helix C-terminal to PAS-B. Thus, the molecular pattern established by the crystal structure tries to explain how the differential protein–protein interaction of the PAS domains in these proteins defines their distinct functions [49, 52, 136]. The occurrence of PAS domains and their interaction is found in many eukaryotic clock proteins [137]. The crystal structure of dPER (Fig. 8a, c) shows a noncrystallographic dimer where the PAS-A domain of one molecule interacts with the PAS-B domain of another molecule. Each PAS domain consists of a five-stranded antiparallel β-sheet (βA-βE) that is covered on one face with several α-helices (αA-αD). PAS-A and PAS-B in each monomer are connected by a short linker. In addition, each monomer has a highly conserved C-domain [138] that includes two long C-terminal α-helices (αE and αF). The αE helix is packed against PAS-B, parallel to αC’ of PAS-B, and the αF helix is directed away from the PAS-B core domain. Also, the crystal structure showed two different conformations for αF in the two dPER monomers [136]. The crystal structure of mPER2 (Fig. 8b, c) reveals a dimer that includes the two PAS domains, the αE helix, and a short N-terminal extension to the PAS-A domain [49].

The PERIOD proteins are known to form homo- and heterodimers in the circadian clock, likely mediated via their PAS domains [138–143]. A detailed structural and biochemical analysis of the PAS domains of the dPER and mPER2 fragments has shown homodimer formation in solution and in crystal. The two structures reveal the use of different PAS interfaces for dimerization. The dPER fragment forms a dimer via intermolecular interactions of PAS-A with Trp482 in the βD’–βE’ loop of PAS-B (PAS-A-Trp482 interface) and with αF in PAS-B (PAS-A-αF interface), whereas in mPER2, the dimerization is stabilized by interactions of two PAS-B domains in antiparallel fashion. Trp419, which corresponds to Trp482 in dPER, is an important conserved residue involved in this interaction [49]. The PAS domains of dPER mediate interactions with dTIM in the *Drosophila* CC [144, 145]. Homodimerization might be important for dPER stabilization in the absence of dTIM and might have a possible role in dTIM-independent transcriptional repression and translocation of dPER [146–151]. However, dPER also interacts with dTIM, and in the absence of structural studies of the heterodimeric complexes a detailed analysis of such an association is difficult. A low-resolution structure of a HIF α (Hypoxia inducible factor α) PAS-B heterodimer (PDB 2A24) was obtained by docking the high-resolution structures of ARNT and the HIF-2α PAS-B domain using experimentally derived NMR restraints for the association. It demonstrated the use of a common β-sheet interface for hetero- and homodimerization in PAS [152]. Additionally, a crystal structure of a dPER fragment lacking αF, combined with a mutant analysis using analytical gel filtration and analytical ultracentrifugation, showed no dimer formation, suggesting that helix αF contributed to dPER homodimer formation [49].

Structural analysis of dPER has shown the importance of the PAS-A-αF interface in homodimer formation in solution. A dPER^L^ (V243D) mutant, which has a temperature-dependent 29-hour long period phenotype, existed as a monomer in the solution [108]. The analysis of dPER structure (Fig. 8d) has shown that V243 is located in the center of the PAS-A-αF interface; thus, the structure provides a mechanistic explanation for the 29-hour long period phenotype of this encoded mutation variant, reflecting the significance of this interface in clock function [49]. Consistent with this study, a PAS-B triple mutation (E474R/H492S/R494D) in a dPER fragment lacking αF disrupted the dPER-dTIM heterodimer in yeast two-hybrid studies, but not the dPER homodimer in gel filtration conditions. The study suggested that the PAS-B β-sheet surface is a common surface in dPER-dTIM heterodimer formation and (mPER2)_2_ homodimerization [49]. The crystal structures of mPER1 and mPER3 (Fig. 9a–f) were analyzed and compared with the previously reported mPER2 structure. In addition to the PAS-B-Trp419 interactions in mPER2 (Trp448 in mPER1 and Trp359 in mPER3), it was revealed that their homodimers are stabilized by further interactions in the PAS-A domain, which are mediated by two antiparallel PAS-A/αC motifs, not by an mPER2-type PAS-A–PAS-B/αE interaction. In the center of the interface is the Tyr267 residue in mPER1 (Tyr179 in mPER3) (Fig. 9c, f). The corresponding residue in dPER is Ala287, which facilitates the introduction of Trp482 into the PAS-A domain binding pocket in dPER and dimer formation that is different from that of mPERs [49, 52].

Despite the conserved domain composition of the mPER proteins, the different interacting interfaces of the homodimers could play a role in defining their distinct functions. Of the three mammalian period proteins, mPER1 and mPER2 have been shown to be more important for maintaining the circadian rhythmicity. mPER2 regulates the expression of the clock genes (interaction with REV-ERBs), while mPER1 maintains their stability and subcellular localization via protein–protein interactions [153–155]. Knockout mouse studies of mPER3 showed only mild circadian phentoypes [156] but affected sleep homeostasis, suggesting its role to be directed more towards the regulation of the output processes than the core clock [157]. Period proteins contribute to the circadian regulation of metabolic pathways in peripheral tissues (adipose, liver, and muscle tissue) via the nuclear receptor signaling pathways. mPER3 interaction, via its PAS domains, with the nuclear receptor Peroxisome proliferator-activated receptor gamma (PPAR-γ) represses the receptor and inhibits adipogenesis [158]. The interactions occur via the PAS domains in mPER3. mPER1 interacts with the mineralocorticoid receptor to positively regulate the basal and aldosterone-mediated expression of the alpha subunit of the renal epithelial sodium channel (αENaC) in the renal cortical collecting duct cells, by binding of the complex to the E-box in αENaC promoter [159].

Analytical gel filtration analysis of the mPER homodimers in solution revealed a higher affinity for the mPER1 homodimer than for mPER2 and mPER3. Structural analysis of the PAS-A/αC interface (Fig. 9c, f) showed small (Gly) residues in mPER1, resulting in tighter PAS-A/αC dimer interaction compared to mPER3, which has a bulky Arg residue. Additionally, all mPER structures showed a highly conserved nuclear export signal (NES) in the αE helix. Mutation of a Met residue in this region of mPER2 disrupted its nuclear export activity, whereas mutation of the corresponding Leu in mPER1 and mPER3 had no effect. Structural analysis revealed the involvement of that Met in homodimer formation, in contrast to its Leu counterpart, which is exposed on the surface because of different orientations of the monomers in mPER1 and mPER3 compared to the (mPER2)_2_ homodimer [49, 52]. These observations suggest that homo- and heterodimerization events direct NES activity.

The N-terminal cap was observed to be unstructured in mPER2, whereas it formed a long helix followed by a β-strand in mPER1 and a shorter helix in mPER3. Sequence analysis of the mPER proteins predicts the presence of a HLH motif N-terminal to the PAS-A domain. In the absence of a basic region of the bHLH transcription factors, the mPERs HLH region might be engaged in heterodimeric interactions with other HLH proteins. Analytical gel filtration and mutation studies showed that mPER3 utilizes the HLH motif as a second interface to further stabilize homodimer formation instead of the PAS-A/αC interface in mPER1 and forms a more stable homodimer than mPER2. Also, a LX**L**L coactivator motif was observed in the PAS-A βE strand of mPER2 [49, 52], which was shown to play a role in the interaction of mPER2 with Rev-erbs [153]. The corresponding motif in mPER1 (PXX**L**L) and in mPER3 (PXX**L**T) is buried deep in the hydrophobic pocket formed by a Trp (in PAS-A) and a Leu residue in the N-terminal cap in mPER1 and mPER3, but not in mPER2. In addition, the coactivator motif in mPER2 is preceded by a less ordered βD-βE loop in the motif, suggesting that the motif in mPER2 is more easily available for interaction with nuclear receptors based on the higher flexibility of the adjoining regions [52]. Analyzing the interacting interfaces, the subsequent orientation of the monomers in mPER homodimers suggests the availability of distinct surfaces for interaction with other clock proteins and nuclear receptors. A recent study developed three new mouse cellular clock models in fibroblasts, adipocytes, and hepatocytes to study cell type-specific functions of clock gene function in peripheral tissues. Such studies showed that, although core-clock gene knockdowns displayed similar phenotypes, the *period* and *Rev-erbs* knockdowns showed cell-specific phenotypes [160].

Structural analysis of the PERIOD protein fragments is a step towards understanding PAS domains and the interactions of the PERIOD proteins. Future mutation studies of the key surfaces found from the structural studies and the interacting partners will provide a detailed understanding of their functions and the mechanism involved, which are not yet clear. Also, the newly developed cell-autnonomous clock model approach can be applied to other cell types which can be utilized to study mutants based on structural analysis to understand the tissue-specific functional differences of various clock genes.

*The CLOCK–BMAL1 complex:* The CLOCK–BMAL1 complex is central to the core oscillator in the mammalian clock. In the primary loop, these positive elements activate the transcription of Per and Cry genes. The PER–CRY complex in return represses their transcription by acting on CLOCK and BMAL1 expression. Another regulatory loop is formed by CLOCK–BMAL1 and Rev-erbs and RORs, wherein the complex activates their transcription. Rev-erbs and RORs subsequently regulate the rhythmic expression of BMAL1 [17, 128, 131]

**Fig. 10:**
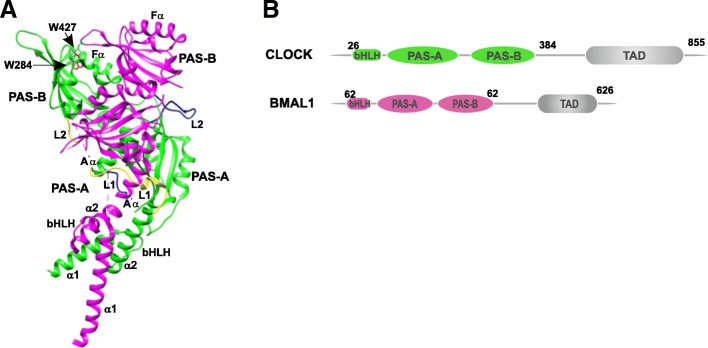
Crystal structure of the mouse CLOCK–BMAL1 complex (PDB 4F3L). **a** The ribbon diagram of the complex shows the CLOCK subunit in *green* and BMAL1 in *pink*. *Yellow* and *blue* highlight the respective linker regions between the domains. **b** Domain architecture of CLOCK and BMAL1 depicting the basic helix-loop-helix domain and the two PAS domains

An important step towards understanding the mammalian circadian clock has been the crystal structure of the mouse transcriptional activator CLOCK–BMAL1 heterodimeric complex that is central to the oscillator [161]. The 2.3-Å resolution structure (Fig. 10) of the complex between CLOCK residues 26–384 and BMAL1 residues 162–447 revealed a tightly intertwined heterodimer formed by the interaction between their corresponding bHLH, PAS-A, and PAS-B domains. The crystal structure showed a striking difference in the spatial arrangement of the corresponding domains in the two proteins. The bHLH domain consists of two helices, α1 and α2, of which α2 is connected to the N-terminal A'α helix of the PAS-A domain via a linker, L1. The CLOCK α2 helix is arranged in such a way that it is in direct contact with the CLOCK PAS-A domain, whereas no such feature is observed in the bHLH and PAS-A domains of BMAL1. Part of helix α1 and helix α2 are involved in the dimerization of the bHLH domains of the two proteins, forming a typical bHLH four-helix bundle similar to that observed in the bHLH-leucine-zipper (LZ)-containing heterodimer MYC-MAX [162]. However, the additional PAS or LZ domain guides their selective and differential partner preference among members of the bHLH superfamily [163]. A proper bHLH four-helix bundle conformation is important for the stability of the CLOCK–BMAL1 complex and its DNA binding activity, as deduced from mutations in the bHLH domain, which resulted in reduced formation of a stable complex and elimination of its transactivation property.

The CLOCK and BMAL1 PAS-A domain consists of a typical PAS fold that is made of five β-strands and several helices. External to the PAS fold is the N-terminal A'α helix that is packed between the β-sheet surfaces of the two PAS-A domains and contributes to the heterodimeric interactions between the two domains. The interactions between CLOCK A'α and the BMAL1 β-sheet, and vice versa, are highly hydrophobic, forming a parallel heterodimer. Simultaneous mutation of the interface residues in both CLOCK and BMAL1 greatly reduced both the heterodimer formation and its transactivation potential compared to single mutations involving the individual proteins. The PAS-B domains of the two proteins are connected to the PAS-A domains by the 15-residue linker L2, which is ordered and buried within the CLOCK–BMAL1 interface in CLOCK, whereas in BMAL1, the linker is exposed to the surface and flexible. The crystal structure showed a translation of 26 Å in the PAS-B domains of CLOCK and BMAL1. The two PAS-B domains interact via surface-exposed hydrophobic residues in CLOCK and BMAL1. Trp427 of BMAL1 stacks with the CLOCK Trp284 located in the hydrophobic cleft between the Fα helix and the AB loop of the CLOCK PAS-B domain (Fig. 10). The tandem mutation of W427A in BMAL1 and W284A in CLOCK resulted in reduced complex formation and reduced the activity of the complex [161].

Lack of similarity among the clock proteins indicates that while the mechanisms are conserved across the kingdoms and are fundamental to clock machinery, the proteins are not structurally related, and further research is required to understand the structural differences. The crystal structures of the PAS domain homodimers of dPER and mPERs provide an interesting view of the interactions and their nonredundant functions. The PAS domains of *Drosophila* dPER share a significant similarity with mammalian PER proteins and bHLH-PAS transcription factors (CYC, BMAL, CLK, and NPAS2) [138]. WC-1, the functional analogue of CLOCK–BMAL1 from fungi, shows some similarity to BMAL1 within the PAS domain, as well as outside of the immediate PAS domain [98], suggesting a common ancestor and providing a link between fungi and animals. A bHLH-PAS domain has also been identified in phytochrome-interacting factor-3 (PIF3), which shows high similarity in the bHLH region to other members of the bHLH protein superfamily. Outside of the bHLH domain, PIF3 shows limited similarity to the PAS domains in phytochromes, but not to animal PAS domains [164]. The secondary dimer interface observed in mPER1 and mPER3 homodimers was absent in (mPER2)_2_ and is a conserved feature of mPER1 and mPER3, but not of other PERs or the bHLH-PAS-containing transcription factors [52]. Thus, the structural studies on dPER and mPER emphasized the need for detailed structural and biochemical analyses of the PERs’ and bHLH-PAS’ transcription factors to determine if similar or different modes of interaction exist among these clock components.

The crystal structure of the heterodimeric complex between mouse CLOCK and BMAL1 revealed an unusual 3D arrangement of the two PAS domains in the two proteins. The conformation and the spatial arrangement of the PAS domains of BMAL1 were similar to that observed in the crystal structure of the PAS domains of dPER and mPER. Trp362 in CLOCK is involved in an interaction with CRY. The corresponding Trp427 in BMAL1 interacts with CLOCK. In PERIOD proteins, Trp at a similar position is involved in homodimer formation [49], suggesting high structural and functional conservation of the BMAL1 and PER PAS domains. Also, the dimerization mode in the PER homodimer crystal structure and in the solution NMR structure of the HIF-2α–ARNT heterodimer was antiparallel, whereas it was parallel in the CLOCK–BMAL1 heterodimer, which, despite the similarity in the structure of the domains, suggests that their protein–protein interactions and/or function are highly influenced by the spatial arrangement [161]. Homo- and hetero-dimerization has also been observed in the components of the plant clock CCA1/LHY that contains the Myb-like domains instead of the bHLH-PAS domain. The interaction occurs in the region at the N-terminus, probably near the Myb domain. Two Myb domains are necessary for DNA binding, and dimerization was observed in the case of single Myb-domain-containing proteins. CCA1 was also observed to form homodimers [165]. However, the functional significance of its dimerization is yet to be determined.

**Fig. 11:**
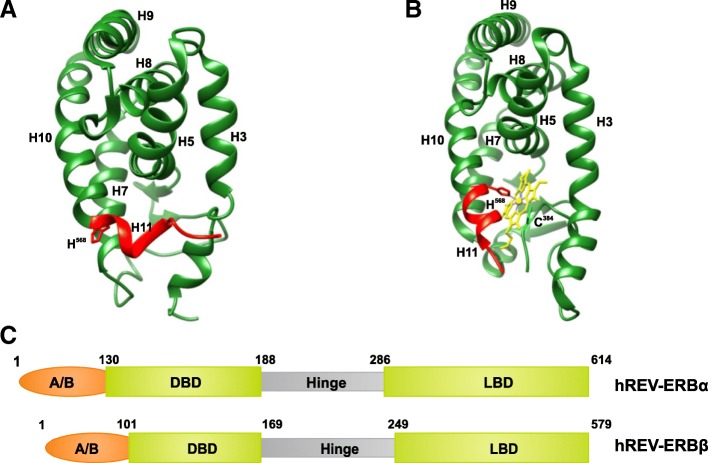
Structure of Rev-erb β LBD monomer. **a** The apo form (*green*), without heme (PDB 2V0V), and **b** as a heme-containing (*yellow*) complex (PDB 3CQV), with the prosthetic group bound in the ligand-binding pocket. The conserved Cys384 (*cyan*) and His568 (*red*) residues involved in heme-binding are shown in stick representation. Helices H11 (*red*) and H3 undergo conformational changes to accommodate the heme prosthetic group. **c** The domain architecture of the Rev-erbs depicting the variable N-terminal A/B region (*orange*), DNA-binding domain (DBD) and the ligand-binding domain (LBD)

*Structural insight into Rev-erb interactions:* The crystal structure of human Rev-erbβ was reported in a dimeric arrangement (Fig. 11a; monomer) [166]. Rev-erbs belong to the family of nuclear receptors that consist of ligand-sensitive transcription factors. These nuclear receptors contain two domains important for their activation: the ligand-insensitive activation domain, called activation function-1 (AF-1), at the N-terminus and the ligand-dependent activation domain, known as AF-2, present within the ligand-binding domain (LBD) at the C-terminus. Rev-erbs are unique within the family in that they lack the AF-2 domain [167]. In addition to being crucial components of the mammalian circadian clock, Rev-erbs are also suggested to play an important role in coordinating the metabolic process [168]. In the crystal structure, each monomer has an α-helical fold that consists of nine α-helices (H3–H11) and short β-strands (s1–s2). The putative LBD is filled with bulky hydrophobic residues, resulting in a small cavity unable to accommodate any potential ligand. Also, in the absence of helix H12 (AF2-helix), helix H11 adopts a unique kinked conformation that establishes contacts with H3, thereby stabilizing the hydrophobic core. H11 provides a structural platform for binding of a co-repressor and is important for constitutive repression activity.

The molecular model of Rev-erbα LBD, constructed using Rev-erbβ LBD as a template, showed a similar configuration in the putative LBD [166], in contrast to the molecular model of LBD for E75, a *Drosophila* orthologue of human Rev-erbα [169], in which the putative LBD was large enough to accommodate a heme ligand. Previously, Rev-erbs were reported to be true orphan nuclear receptors showing no ligand-binding activity and acting as constitutive repressors by their binding to the nuclear co-repressor (N-coR). Similar to heme protein E75 [169], studies showed that heme is required to maintain the stability of Rev-erbs. The heme binding was found to be reversible, and the transcriptional regulation of Rev-erbs is altered with changes in concentration of the heme in the intracellular environment. Heme binding is required to stabilize N-coR interaction with the Rev-erbs [170, 171]. The crystal structure of the heme-bound Rev-erbβ LBD (Fig. 11b) [172] coupled with spectroscopic analysis provides the structural basis to show that heme and gas molecule (NO or CO) binding and the redox state are important for the regulation of Rev-erb activity. Conserved Cys and His residues were observed to be essential for heme binding, where Cys384 coordinates oxidized Fe(III), but not reduced Fe(II). These redox-dependent structural changes, resulting in functional changes, are common in heme proteins, such as E75 and NPAS2, but it is not known if the same changes happen in Rev-erbs [169, 173]. The reduced form was also able to bind gas molecules. Compared to the apo LBD structure, in the Rev-erbβ LBD complexed with oxidized Fe(III), helix H3 becomes straight, and H11 undergoes a conformational change in its C-terminal half to allow accommodation of the two heme-binding residues. The hydrophobic residues filling the LBD stabilize heme binding via van der Waals interactions, suggesting a significant contribution to binding strength and specificity. Heme has been shown to influence circadian cycles and to be a component not only of Rev-erbs but also of other CC proteins, such as mPER and NASP2 [172].

**Fig. 12:**
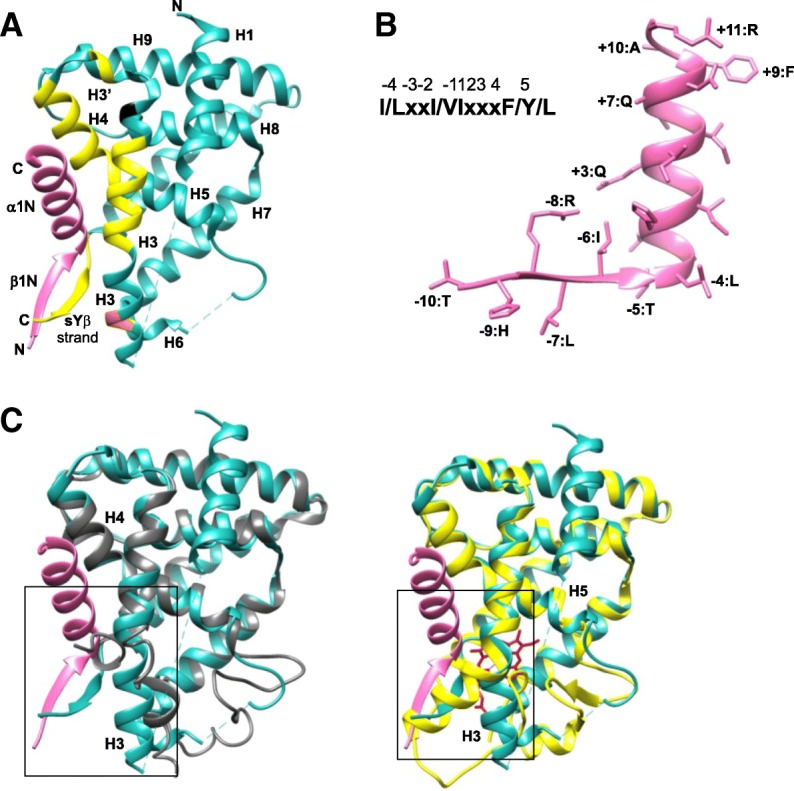
Structure of N-CoR ID1 peptide and interactions. **a** N-CoR ID1CoRNR peptide (*pink*) bound to Rev-erb αΔ 323-423 LBD (*sea green*; PDB 3N00) depicting the N-CoR ID1 peptide β-strand (β1N) and α-helix (α1N) and the new C-terminal β-strand sY of Rev-erb α LBD. The backbone of the contact residues in H3, H4, H5, and the new Yβ-strand are shown in *yellow* and the supporting H3 residues in *orange*. **b** Representation of the amino acid residue positions in the N-CoR ID1 peptide defining the new extended motif for NRCoR. **c** Comparison of the N-CoR ID1 CoRNR peptide (*pink*) bound to Rev-erb αΔ 323-423 LBD (*sea green*) with apo-Rev-erb β (*gray*) and heme (*red*)-bound Rev-erb β (*yellow*). The region within the *black box* represents the changes in H3 as a result of conformational changes in H11 when Rev-erb binds to N-CoR ID1/heme.

In the absence of the AF2 domain, the Rev-erbs regulate the activity of various genes via association with the nuclear receptor-co-repressor (N-coR) [168, 174, 175]. N-coR consists of two regions, called interaction domains (ID) 1 and 2, through which it binds to the nuclear receptor LBD. Rev-erbs regulate gene activity by specifically binding to the ID1 CoRNR motif [176–178]. Structures of apo-Rev-erbβ and heme-bound Rev-erbβ, however, are unable to help in understanding the Rev-erb–N-coR association, which is important for its repressive function. Phelan et al. [179] studied a co-crystal structure of interaction domain 1 (ID1) peptide bound to the hRev-erbα LBD (Fig. 12). The structure revealed formation of β-structures at the C-terminal region of the LBD that have not been observed in other nuclear receptors or in apo- or heme-bound Rev-erbβ. The N-coR ID1 peptide association with the C-terminal region of the Rev-erbα LBD results in an antiparallel β-sheet formation. The N-terminal β-strand (β1N) of the N-coR ID1 peptide is followed by a well-defined α-helix (α1N) that extends into the coactivator groove of the LBD. Structure-based alignment of the N-coR ID1 peptide-bound Rev-erbα with N-coR2/SMRT1 ID2-bound PPARα defines a new and extended CoRNR motif (I/LxxI/VIxxxF/Y/L) (Fig. 12b) that best describes the binding requirements for ID1 and ID2. Mutations at the +1, +4, and +5 positions that form the core of the CoRNR motif showed significant reduction in binding affinity towards Rev-erbα. Similar results were observed in a mammalian two-hybrid assay. Mutation at the +9 position resulted in nine-fold reduction of the interaction. These observations suggest that the core CoRNR motif (ICQII) and the right-extended flanking region are required for the interaction with Rev-erbα. Comparison of the N-coR ID1-bound Rev-erbα LBD with apo-Rev-erbβ and the heme-bound Rev-erbβ (Fig. 12c) showed that heme binding brings about changes in the conformation of H11 that result in large changes in H3, which then occupies the space for ID1 N-coR-binding. Based on homology, if the heme-bound Rev-erbα adopts similar changes, they will affect the binding of Rev-erbα with N-coR ID1 [179]. Also, the binding of heme with Rev-erbα destabilized interaction with the N-coR peptide [170], suggesting that N-coR associates differentially with the Rev-erbs in the absence of heme to perform the repression function and that the interaction between full-length Rev-erb and N-coR in the presence of heme might require additional contacts between the two proteins [174, 179].

One study has shown that both Rev-erbα and β [180] are central to the circadian clock, playing an important role in the regulation of the core-clock components and the clock output genes, rather than forming an accessory loop contributing to clock function. Analysis of the genome-wide *cis*-acting targets of the two isoforms and comparison with the BMAL1-binding sites [181] showed an extensive overlap highly enriched in the circadian clock genes and lipid metabolism genes. The integral role of Rev-erbs closely associates metabolic regulation to the core-clock machinery, and any alterations in the core-clock genes would create disturbance in energy homeostasis and metabolic activities that could eventually lead to metabolic diseases. The double-knockout mutant of Rev-erbα and β showed phenotypes with severely disrupted circadian expression of the core-clock components and deregulated lipid homeostatic genes. The circadian phenotypes were similar to those observed in other core-clock mutants (*Bmal1*^*-/-*^, *Per1*^*-/-*^*Per2*^*-/-*^*, Cry1*^*-/-*^*Cry2*^*-/-*^), suggesting that, together, the two Rev-erbs work with BMAL1 and other core-clock components to regulate circadian rhythms and metabolism. [180]. Additionally, the knowledge that a small-molecule ligand, like heme, is essential in the regulation of Rev-erbs’ activity has driven scientists to develop synthetic Rev-erb agonists as a new therapeutic approach for the treatment of metabolic diseases and resetting of altered circadian rhythms [182].

### The plant circadian clock

The plant CC has been comprehensively studied using *Arabidopsis thaliana* as a model. The present clock paradigm consists of at least three interlocking transcriptional–translational feedback loops (Fig. 3e) [183, 184]. The core loop includes two related MyB-like transcription factors, CIRCADIAN CLOCK ASSOCIATED 1 (CCA1) and LATE ELONGATED HYPOCOTYL (LHY), whose expression peaks in the morning, and TIMING OF CAB EXPRESSION 1 (TOC1), which is expressed in the evening. TOC1 is a member of the pseudo-response regulator (PRR) gene family (PRR3, PRR5, PRR7, PRR9, and TOC1), whose members are clock-regulated but peak at different times of the day [185–189]. The nuclear-localized TOC1 protein, earlier suggested to activate CCA1/LHY expression [190], is the transcriptional repressor of CCA1 and LHY [191], and CCA1 and LHY repress TOC1 activity [192–194]. In the morning loop, CCA1/LHY promotes PRR9 and PRR7 expression, which, in turn, have negative feedback on CCA1/LHY [195–197]. In the evening loop, TOC1 represses an unknown mathematically defined factor ‘Y’ that, in turn, activates TOC1 expression. GIGANTEA (GI) [198] is thought to be a part of the Y factor. GI itself is negatively regulated by CCA1/LHY and TOC1 [199].

Another evening-expressed MyB domain-containing SHAQYF-type GARP transcription factor, LUX ARRHYTHMO (LUX), functions in a feedback role similar to that of TOC1 [200, 201] and is a possible component of a proposed Y activity [200]. Other components important for the clock, such as EARLY FLOWERING 3 and 4 (ELF3 and ELF4), are necessary for the gating of light signal inputs into the clock via an unclear mechanism. ELF3 and ELF4 are highly conserved plant-specific nuclear proteins with unknown function that normally accumulate in the evening [202–206]. Loss-of-function mutations in these three clock components result in arrhythmia under conditions of constant light and in darkness [200, 201, 205, 206]. Recent studies have shown them to be integral components of the evening repressor complex of the core molecular oscillator important for proper functioning of the circadian clock, and they have been implicated in the regulation of the transcript levels of *PRR9* [206–211]. Repression by the evening genes was inferred from the genetic studies of ELF4 and ELF3 [212, 213]. Taken together, the plant CC appears to be comprised of a series of transcript regulators specific to plants.

The plant clock components and their interactions have primarily been studied using reporter assays, the yeast two-hybrid assay, and co-immunoprecipitation. However, lack of structural knowledge is largely limiting our understanding of the clock components. In silico approaches have been applied to predict the structural features and thereby gain insight into the underlying functional aspects of some components. However, in the absence of experimental validation, a cautious approach is required. Using such an approach, TOC1 was predicted to be a multidomain protein, having an N-terminal signaling domain as well as a C-terminal domain that might be involved in metal binding and transcriptional regulation. A middle linker predicted to lack structure connects two domains [214]. The N-terminal domain fold is predicted to be similar to the canonical fold of the bacterial RR protein structures [215, 216], hence the name PRR. The RR class of proteins is involved in phosphor-relay signaling in bacteria and plants [217, 218]. Gendron et al. [191] have recently defined the biochemical function of TOC1 in transcriptional repression that resides within its PRR domain. The extreme end of the C-domain is predicted to have two α-helices and represent a CCT (for CONSTANS, CONSTANS-like and TOC1) subdomain similar to the CCT domain of CONSTANS (CO). Since CO interacts with the HEME ACTIVATOR PROTEIN (HAP) transcription factor, Wenkel et al. [219] suggested that the CCT subdomain of TOC1 could have a similar interaction with this class of DNA-binding proteins, thus implicating TOC1 as a co-regulator of transcription [214]. Work by Gendron et al. [191] confirmed this structural hypothesis [214] by showing that TOC1 belongs to the family of DNA-binding transcriptional regulators. They showed that TOC1 could bind to DNA through its CCT domain and that a functional CCT domain is a prerequisite for the repressor activity of the PRR domain [191].

**Fig. 13:**
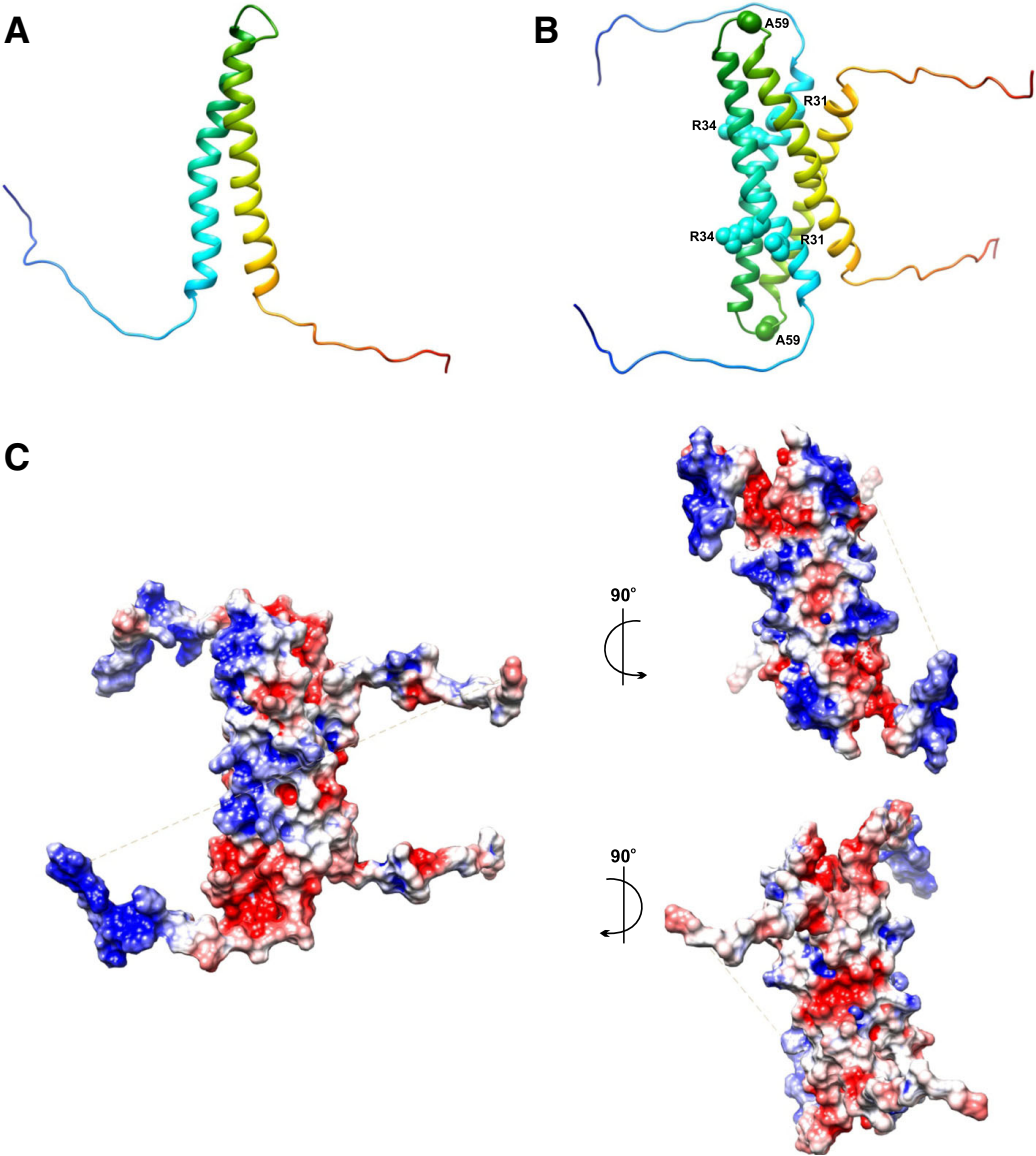
Predicted structural models of ELF4. The **a** ELF4 monomer, **b** ELF4 dimer, and **c** electrostatic potential surface calculated for the ELF4 dimer. Surface areas colored *red* and *blue* represent negative and positive electrostatic potential, respectively

Another study utilizing bioinformatics approaches [212] has predicted that ELF4 is a protein with a single domain of unknown function and that it belongs to a functionally conserved family of ELF4 and ELF4-like proteins. The conserved region is predicted (Fig. 13a) to be α-helical with a coiled-coil structure and disordered N- and C-termini. The secondary structure analysis using CD spectroscopy showed signals for disordered regions and an α helix, but not for β-sheet conformation. The protein migrated as a dimer on a native gel. Using docking programs, ELF4 was predicted to form a homodimer with an asymmetrical electrostatic-potential surface (Fig. 13b, c). Additionally, expression analysis of *elf4* hypomorphic alleles showed phenotypes at both morning and evening genes, suggesting a dual role for ELF4 linked with both morning and evening loops [212]. ELF4 influenced the clock period by regulating the expression of LUX under LL, in addition to TOC1, PRR9, and PRR7 expression under DD. The effect of ELF4 on morning and evening loops did not alter CCA1 or LHY expression [212].

Identification of the evening complex, comprised of ELF4, ELF3, and LUX, which are all crucial for the transcriptional repression of the morning genes, addresses the importance of protein–protein interactions in a functional rhythmic oscillator [207]. ELF4, previously predicted to activate a transcriptional repressor [212], was shown to interact genetically and physically, both in vivo and in vitro, with a middle domain in ELF3. The interaction between the two proteins increased the nuclear levels of ELF3, suggesting that ELF4 acts as an anchor that helps in nuclear accumulation of ELF3. Both the nuclear-localization region in the C-terminal domain and the ELF4-binding middle domain of ELF3 were observed to be important for functional activity of ELF3 [211]. Although the biochemical activity of ELF3 is unclear, it has been proposed to be a co-repressor of *PRR9* transcription [209].

## Light: input to the clock

Light is one of the major environmental cues influencing the CC. Organisms have evolved sophisticated light-signaling networks that synchronize the clock to day/night cycles in order to regulate their metabolic and physiological processes.

### Cyanobacteria

Cyanobacterial rhythms are shown to be synchronized indirectly by light via the redox state of metabolism in the cell. The type of input that the clock perceives was previously unclear. Further work revealed Circadian input kinase A (CikA), a histidine kinase bacteriochrome [220], and light-dependent period A (LdpA), an iron-sulfur protein [221], to be important candidates for input signaling to the core oscillator. These proteins transmit the input signals by sensing the redox states of the plastoquinone (PQ) pool. The PQ redox state in photosynthetic organisms varies with the intensity of light: PQ is oxidized under low light intensities and reduced at high light intensities [222]. A CiKA mutant showed a shorter free running period and was unable to reset after a dark pulse [220]. Like CikA mutants, LdpA mutants also showed a short circadian period; however, they were able to reset after the dark pulse [221]. CikA protein levels vary inversely to the light intensity in the wild type, but were observed to be light insensitive in the absence of LdpA [221, 223, 224]. *S. elongatus* CiKA (SyCiKA) consists of a cGMP phosphodiesterase/adenylate cyclase/FhlA-like domain (GAF) similar to that in other bacteriophytochromes, followed by a characteristic histidine protein kinase (HPK) domain. However, the GAF domain lacks the conserved Cys and His needed for the binding of the chromophore in other bacteriophytochromes. Also, binding with a chromophore was not observed in vivo*.* C-terminal to the kinase motif is the receiver domain homologous to the receiver domain of the response regulators of the bacterial two-component signaling systems. It lacks a conserved Asp present in the receiver domains of the bacterial RRs that is phosphorylated by the HPK domain, hence the name pseudoreceiver domain (PsR) [220, 225]. A family of PsRs is also observed in the plant circadian clock (PRRs) [185].

**Fig. 14:**
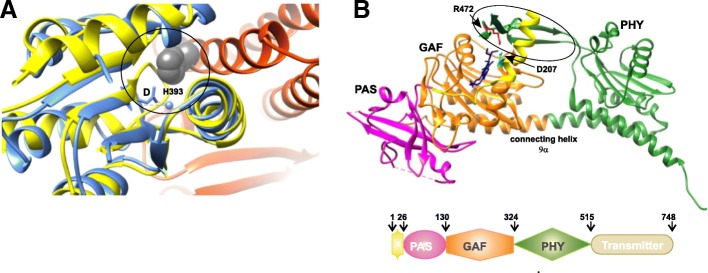
Structure of the PsR domain of CiKA. **a** CiKA-PsR (*yellow*, PDB 2J48) superimposed on the Spo0F–Spo0B complex (*blue* and *orange*, PDB 1F51) depicting the structural difference in the HPK-PsR domain interaction interface in CiKA and bacterial Spo0F–Spo0B. **b** The complete phytochrome sensory module of *Synechocystis* 6803 Cph1 (PDB 2VEA). The tongue region is encircled. The N-terminal region is shown in *yellow*, the PAS domain in *pink*, the GAF domain in *orange*, and the PHY domain in *green*. The phycocyanobilin (PCB) chromophore is shown in *blue* stick representation

The solution structure of the PsR of CiKA (PDB 2J48) [226] consists of a doubly wound five-stranded β-sheet with five α-helices (α1 and α5 on one face and α2–4 on the other). CiKA mutants lacking the PsR domain showed significant increase in autokinase activity [225]. The interaction between the PsR domain and the HPK domain of CiKA was analyzed by superimposing a predicted model of CiKA-HPK (using PDB 2C2A as template [227]) and the solution structure of CiKA-PsR over the Spo0F–Spo0B complex (PDB 1F51 [228]) crystal structure. The PsR domain physically blocked the H393 of the HPK domain, making it unavailable for phosphoryl transfer (Fig. 14a), which explains the role of PsR in the attenuation of CiKA-HPK autophosphorylation activity [226]. Phopshorylation of the receiver domain in the bacterial RRs results in a conformation change, an effect that is probably mediated by the protein–protein interaction in CiKA. Like CiKA, KaiA also consists of a pseudo-response receiver domain at the N-terminus. In KaiA homodimers, the interaction between the two protomers occurs via the α4-β5-α5 surface of the PsR domain of one subunit with the swapped C-terminal domain of the other [44, 60]. It was expected that CiKA might use the same PsR surface to mediate protein–protein interactions.

The phosphatase activity of CikA is enhanced significantly in the presence of KaiC and KaiB. In vivo, *CikA*^*−*^ strains showed high levels of phosphorylated RpaA, indicating CikA promotes dephosphorylation of RpaA [229]. Also, relative to the gsKaiB, fsKaiB variants showed a threefold increase in phosphatase activity of CikA and suppressed RpaA phosphorylation, suggesting that the rare active state KaiB interaction with KaiC activates signaling through CikA. Shortened periods of oscillation were observed in vivo and in vitro in the presence of excess of the pseudo-receiver domain of CikA (PsR-CikA). CikA was proposed to interact physically through its pseudo-reciever domain. Also, interactions were observed for KaiB variants (that adopt the fsKaiB state) and PsR-CikA domain but not for PsR-CikA domain and gsKaiB [88]. To understand the molecular basis of this interaction, a study was undertaken using Methyl-TROSY NMR spectroscopy and this revealed that an interaction between PsR-CikA and the KaiC CI domain–fsKaiB complex. Nuclear magnetic resonance spectroscopy (NMR spectra) were similar for PsR-CikA bound to fsKaiB–KaiC CI or wild-type KaiB–KaiC CI complexes. Co-operative assembly is also essential for the formation of the CikA–KaiB–KaiC complex, similar to what is observed during the formation of the KaiA–KaiB–KaiC complex, as observed by weak interaction between PsR-CikA and fsKaiB in the absence of the KaiC CI domain [75].

**Fig. 15:**
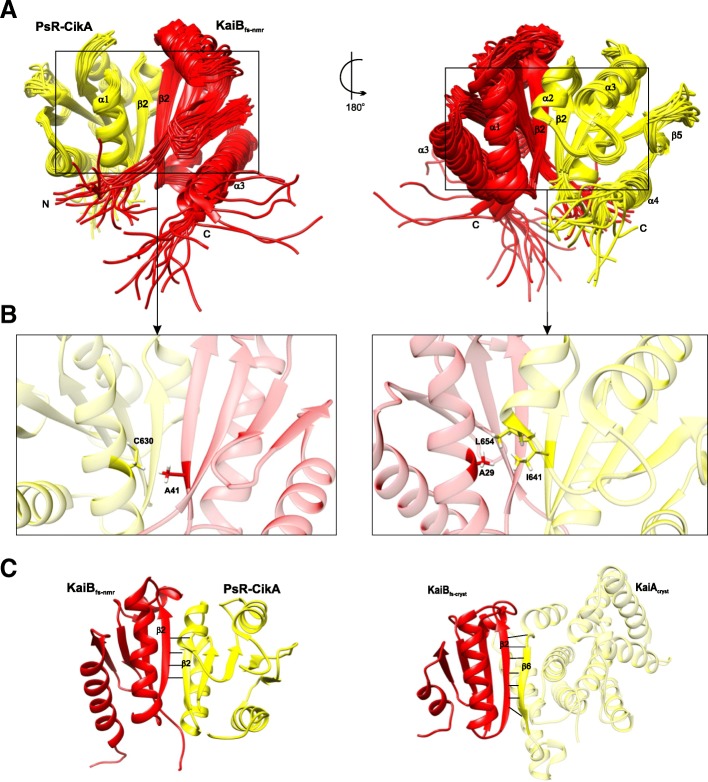
Structural analysis of the PsR–CikA–KaiB_fs-nmr_ complex and the interacting interface. **a** NMR structure of the PsR–CikA–KaiB_fs-nmr_ complex. *Yellow*, PsR-CikA; *red*, KaiB_fs-nmr_. **b** An expanded, close-up view of the boxed region depicting the complex interface is shown**. c** Comparison of the PsR–CikA–KaiB_fs-nmr_ and KaiA_cryst_–KaiB_fs-cryst_ complex interfaces. PsR–CikA and KaiA_cryst_ compete for the same β2 strand of rare active fsKaiB

The solution structure of the complex between a fsKaiB variant with N29A substitution (KaiB_fs-nmr_ ; binds to PsR-CikA in the absence of KaiC CI) and PsR-CikA (Fig. 15a) shows a binding interface of parallel nine-stranded β-sheets that includes β2 of PsR-CikA and β2 of KaiB_fs-nmr_. Structural analysis shows hydrophobic interactions between A29 of KaiB_fs-nmr_ and I641 and L654 of PsR-CikA. The residue I641 of PsR-CikA is located in the center of the β2–β2 heterodimeric-binding interface. The interface center also shows interaction between C630_PsR-CikA_ and A41 of KaiB_fs-nmr_. C630R substitution eliminated complex formation. Comparison of the binding interface of the PsR-CikA and fsKaiB N29A variant complex with that of the KaiA and fsKaiB complex (Fig. 15b) shows fsKaiB uses the same β2 strand to interact with KaiA and CikA. Also, mutations in the β2 strand of KaiB weakened its binding to both KaiA and CikA [75]. CikA and KaiA compete for the same overlapping binding site of the active state KaiB; thus, the rare active fold switched state is important for CikA interaction with the Kai oscillator to regulate input signals, as it is for the inactivation of SasA and the regulation of output pathways.

CiKA and KaiA co-purify with LdpA [224]. LdpA, an iron-sulfur center-containing protein, has been reported to be involved in redox sensing [221, 224]. Treatment of cells expressing LdpA with 2,5-dibromo-3-methyl-6-isopropyl-*p*-benzoquinone (DBMIB), which inhibits electron transfer from PQ to cytochrome *bf*, thus reducing the PQ pool, significantly affected the stability of LdpA, CikA, and KaiA. Additionally, lack of LdpA in DBMIB-treated cells further reduced CiKA stability, suggesting that LdpA can affect CiKA sensitivity to the cellular redox state [224]. Interestingly CiKA and KaiA bind directly to quinone analogues [223, 230], suggesting they can input light signals by sensing the redox state of metabolism in a manner independent of LdpA. Thus, CiKA and LdpA might be a part of an interactive network of input pathways that entrains the core oscillator by sensing the redox state of the cell as a function of light.

### Fungi

Known light-induced responses in *Neurospora* are mediated by the blue light photoreceptors WC-1 and VVD [231, 232]. Light activation and photoadaptation mechanisms are crucial for robust circadian rhythms in *Neurospora* and are driven by the two LOV domains containing WCC complex and VVD [233, 234]. VVD is smaller than WC-1 and works in an antagonistic way to tune the *Neurospora* clock in response to blue light [2]. Light irradiation of the WCC complex results in the formation of a slowly migrating, large WCC homodimer that binds rapidly to the LREs (light responsive elements) and drives the expression of many downstream light-dependent genes (e.g., *frq* and *vvd*) [2, 101, 105, 107]. Light-induced gene expression is a transient process as hypophosprylated WCC, when activated, is simultaneously phosphorylated and rapidly degraded. Phosphorylation of WCC results in the dissociation of the complex, making it unavailable for photoactivation. The gene transcripts and proteins reach a maximum level in the initial 15 and 30 minutes, respectively, and then decrease to a steady state level in an hour on prolonged light exposure, a process called photoadaptation. A second pulse of high intensity can again activate the adapted state gene expression, elevating the levels to a second steady state [2, 232, 233]. As shown in phototropin-LOV2 domains, illumination of the LOV domain results in the formation of a covalent cysteinyl-flavin-adduct formation between LOV domain and FAD/FMN. The conversion of this light-induced adduct back to the dark state is a slow process in fungi, in contrast to the phototropins where conversion occurs within seconds [97, 235, 236].

The expression of *vvd* is under the control of photoactive WCC, and it accumulates rapidly upon irradiation. VVD indirectly regulates the light input to the *Neurospora* clock by repressing the activity of the WCC. Studies show that VVD plays a role in modulating the photoadaption state by sensing changes in light intensity [232]. Recent studies suggest that the competitive interaction of the two antagonistic photoreceptors (WCC and VVD) is the underlying molecular mechanism that leads to photoadaptation. VVD binds to the activated WCC, thus competing with the formation of the large WCC homodimer and, in turn, resulting in the accumulation of inactive WCC and attenuation of the transcriptional activity of the light-activated WCC [237]. Direct interaction of VVD with WCC prevents its degradation and stabilizes it through the slow cycle of conversion back to dark-state WCC [237, 238]. Therefore, the level of VVD helps to maintain a pool of photoactive and dark-state-inactive WCC in equilibrium. Perturbation by a light pulse of high intensity can again result in the photoactivation of the dark-state WCC, disturbing the equilibrium, until the transiently transcriptionally active WCC again drives the accumulation of more VVD to reach a second steady state. Thus, VVD plays a dual role of desensitizing the clock to moderate fluctuations in the light intensity while promoting light resetting to increasing changes in the light intensity. VVD levels gradually decline during the night as a result of degradation, but enough protein is still present to suppress the activation of highly light-sensitive WCC by light of lower intensity (moonlight). Hence, the accumulated levels of VVD provide a memory of the previous daylight to prevent light resetting by ambiguous light exposures [2, 233, 234].

The LOV domain forms a subclass of the PAS domain superfamily; it mediates blue light-induced responses in bacteria, plants, and fungi [2]. In *Neurospora*, VVD and WC-1 are the two LOV domain-containing photoreceptors, and in *Arabidopsis*, the LOV-containing families include phototropins (phot 1 and phot 2) and the ZEITLUPE family (ZTL, LOV kelch Protein 2 (LKP2), and Flavin-binding Kelch F-box1 (FKF1)). They bind the flavin mononucleotide (FMN) chromophore [239].

The crystal structure of VVD-36 (36-residue N-terminal truncation for increased solubility and stability) that retains wild-type behavior studied in both dark- and light-adapted states explains the light-induced conformational changes that are important for VVD activity (refer to [106] for structure figures). The protein exists in the crystal as a symmetrical dimer formed via hydrophobic interactions at the N-terminal cap surface. The structure revealed a typical PAS domain [106] as seen in other PAS domain-containing proteins [240, 241]. Specific to the VVD-like LOV domain is an 11-residue loop between Eα and Fα that accommodates the FAD adenosine moiety exposed to the solvent, and an N-terminal cap (residues 37–70) comprised of helix aα and strand bβ [106]. On photoexcitation, the crystal structure of the light-adapted VVD-36 reveals the formation of a stable covalent cysteinyl-flavin adduct that leads to conformational changes at Gln182 and protonation of the flavin ring. Gln182 flips to overcome unfavorable interactions and, at the same time, maintain hydrogen bonding with the protonated N5 atom of the flavin ring. A rotation of Cys71 breaks its S-H…O hydrogen bond with the carbonyl of Asp68. This brings Cys71 to a more exposed position where it interacts with the peptide N atom of Asp68. The changes in Cys71 conformation shift bβ towards the PAS core and disrupt the interactions that stabilize the packing of the N-terminal cap against the PAS β-sheet. A Q182L mutant showed similar spectral properties on photoexcitation, but it was unable to switch from a compact to a fully expanded form. Conformational changes, which involved the N-terminal cap, were also absent in a C71S mutant, but the photochemical changes at the active center were unaffected. The crystal structure of a C71S mutant (PDB 2PD8) [242] revealed a stronger hydrogen bond formation between Ser71 and Asp68 than in the wild type, which might stiffen the fold, preventing movement.

The crystal structures reveal that light-induced changes in the flavin protonation state lead to conformational changes of N-cap, creating a new interface for dimerization in the light-state VVD. Observations from size-exclusion chromatography together with static and dynamic light scattering (SLS and DLS, respectively) studies show that the light-adapted VVD forms a rapidly exchanging dimer relative to the dark-state monomer, with an expanding hydrodynamic radius. Dimer formation was observed to be concentration-dependent. The increase in the hydrodynamic radius was observed to be highly dependent on the length of the N-terminus. Studies of the various light-state N-cap variants indicate that residues 39–42 are important for dimerization and contain a Pro-Gly-Gly signature that is highly conserved among the dimer-forming variants. The aa39–42 segment adopts two distinct conformations in the crystallographic C71V VVD dimer (PDB 3D72), with a 180° rotation about the Pro residue between the two subunits [242], highlighting the importance of the proline in projecting the N-terminus towards the other subunit. Thus, conformational changes at the hinge to the PAS core and the N-cap Pro-Gly-Gly sequence are critical for light-induced dimerization.

### Plants

Light input in plants is mediated by multiple photoreceptors: phytochromes (red/far-red light photoreceptors), cryptochromes (UV-A/blue light photoreceptors), UVR8 (UV receptor), and ZEITLUPE (ZTL), FLAVINBINDING, KELCH REPEAT, F-BOX 1 (FKF1), and LOV KELCH PROTEIN 2 (LKP2) (blue light) are a suite of photoreceptors involved in the photoentrainment of plants [243]. *Arabidopsis* contains five phytochromes (PHYA–E) [244, 245] and two classic cryptochromes (CRY1 and CRY2 that localize in the nucleus) [246, 247]. A third cryptochrome identified in *Arabidopsis* is called the CRY3/*Arabidopsis* CRY-DASH and has sequence similarity to CRY-DASH from *Synechocystis*. It localizes in chloroplast and mitochondria. CRY3 shows sequence-independent DNA binding, but its role in biological signaling remains unknown [248, 249]. ZTL, FKF1, and LKP2 are single LOV domain blue light receptors [250].

Recent studies suggested several light-signaling pathways involved in clock entrainment, but they are not yet well understood. One such pathway is proposed by the PIF hypothesis [244]. PIFs negatively control light-mediated gene expression to regulate plant development. PIF3 interaction with the light-activated form (Pfr form) of phyB in the nucleus results in its phosphorylation and subsequent degradation, thus relieving the repressive function of PIF3. Also, PIF3 binds to the G-box promoter region of *CCA1* and *LHY* [251] in vitro, suggesting that phyB can also interact with the bound PIF3. In the second signaling pathway, ZTL, FKF1, and LOV-KELCH proteins possibly interact with phyB/CRY1, thus affecting the phyB/CRY1 response to the clock [252]. Also, F-box and Kelch repeat domain of ZTL/FKF1/LKP2 proteins play a role in the regulation of protein stability and mediate ubiquitin/proteasome-dependent degradation as a function of light [253]. Some of their targets include GI, TOC1, and PPR5 [189, 254, 255]. Another possible path consists of the PRR family of *Arabidopsis*, which shows light-dependent effects on clock period [199]. Moreover, proteins that do not possess a chromophore, including ELF4, ELF3, and TIME FOR COFFEE (TIC), are involved in gating light inputs to the clock. ELF3 negatively regulates the light input to the clock. Interaction between ELF3 and phyB results in ELF3's inhibitory function in the subjective night [199, 202, 206, 256].

Phytochromes are red/far-red light-sensing photochromic biliprotein photoreceptors that are involved in the regulation of various developmental processes. Of the five phytochromes found in plants, phyA and phyB are the best characterized [257]. PHYs have been implicated in the regulation of circadian rhythms in plants [257, 258], but their role in clock entrainment in other organisms has not been clearly defined [259]. Phytochromes share a common domain organization. The N-terminal photosensory core module consists of PAS, GAF (cGMP phosphodiesterase/adenylate cyclase/FhlA), and PHY (phytochrome-specific) domains. The PAS and GAF domains are connected by a figure-eight knot. The C-terminal transmitter module consists of an HLH dimerization/phosphor-acceptor domain and an ATPase catalytic domain, and transmits signals perceived by the N-terminal region to the signal transduction pathways. In addition, plant phytochromes contain a "Quail module" between the photosensory and transmitter modules. Fungal phytochromes have an N-terminal variable extension preceding the PAS domain and are not homologous to plant phytochromes. In cyanobacteriochromes, unlike other cyanobacterial phytochromes, the knot and the preceding PAS domain are absent altogether. The GAF domain is self-sufficient for photoperception [256, 260, 261].

The crystal structures of the PAS-GAF two-domain construct of the bacteriophytochrome DrBphP from *Deinococcus radiodurans* [262] and RpBhP3 from *Rhodopseudomonas palustris* [263] lack the PHY domain that is important for the light sensory function as changes in it prevent the conversion from the Pr (phytochrome photochromic state absorbing maximally in the red region) to the Pfr state (maximal absorption in the far-red wavelength range). The crystal structures for the complete sensory module were solved for *Synechocystis* 6803 Cph1 (PDB 2VEA) for the Pr ground state [264] and for bacteriochrome PaBphp (PDB 3C2W) for the unusual Pfr state [265]. Together, these structures show the sensory module to be an asymmetrical dumbbell of PAS-GAF and a smaller PHY fragment. The structure of 2VEA (Fig. 14b) reveals that the PHY domain is a member of the GAF family. It is connected to the PAS-GAF lobe by a long α9 helix. The PHY domain has an unusual tongue-like hairpin loop that contacts the PAS-GAF domain and seals the chromophore pocket. Unlike 2VEA, where the tongue makes intimate contact with the N-terminal helix α1, 3C2W exhibits a different structure for the N-terminal part and the tongue, where the biliverdin chromophore sits and remains exposed. In addition, two salt bridges are important for phytochrome function. One is formed between Arg472 of the tongue and Asp207 in the chromophore pocket. Arg254 forms the second bridge with ring B of the chromophore. In addition to this salt bridge, the main-chain O atom of Asp207 forms a hydrogen bond with the protonated N atom of ring A, B, and C of the chromophore [261, 265]. This interaction seems to be important for the functioning of Pfr, as mutations disturbing the salt bridge affect Pfr function.

The structures of the Pr (2VEA) and Pfr (3C2W) states show that, on excitation, transition from Pr→Pfr leads to Z–E isomerization of the chromophore D ring, consistent with Pr–Pfr photochemistry. Differences were also seen in the position of several tyrosine residues around the D ring in 3C2W. These Tyr residues were shown to be important for photoconversion [261].

Based on the structures of the bacterial phytochromes and the *Arabidopsis* phytochrome mutants studied previously, Nagtani [266] detailed the structure–function relationship of plant phytochromes. The core light-signaling domain corresponds to the N-terminal moiety (N-terminal extension, PAS/GAF domain) as the N-terminal region lacking the PHY domain in phyB continued to exhibit the light signal transduction, instead of the C-terminal region, which consists of a histidine-kinase-related domain (HKRD), as was previously proposed. Also, loss-of-function mutations (in the chromophore pocket and the PAS domain) and gain-of-function mutations (in the GAF domain) in phyA and phyB affected chromophore incorporation and phytochrome stability, respectively. The mutations affecting phyB–PIF interaction were largely found in the light-sensing knot and were identical to the signaling mutants, suggesting the involvement of the light-sensing knot region in the phyB–PIF interaction that initiates the downstream light signaling pathway. Additionally, mutations in the PHY domain that positively or negatively affected Pfr stability were mainly confined to the tongue region, defining the importance of this region in modulating phytochrome activity. Lastly, mutational analysis of the C-terminal region that comprises HKRD suggested it plays a role in protein–protein interaction and nuclear localization [266].

The LOV domain-containing ZTL/FKF1/LKP2 family is involved in the regulation of photoperiodic-dependent flowering and the entrainment of the circadian clock [239]. The structure of the FKF1-LOV polypeptide, a distant relative of VVD, was studied using size-exclusion chromatography and SAXS. FKF1-LOV was observed to be a homodimer with an overall structure similar to that of phot1-LOV (phototropin-LOV domain). Although only small conformational changes were seen in the FKF1-LOV core on dark-to-light activation, interactions with other segments, such as F-Box and/or Kelch repeats, may amplify these changes to initiate a photoperiodic response [267].

The LOV domain in the ZTL/FKF1/LKP2 family undergoes photochemical cycles similar to phot-LOV domains in vitro [253, 268–270]. Upon blue light absorption by phot-LOV, the FMN chromophore in the LOV domain converts from the ground state to a singlet-excited state and further to a triplet-excited state that results in stable photo-adduct formation between FMN and a conserved Cys of the LOV domain. Reversion to the ground state is also rapid [271]. The slower adduct formation and dark recovery rates of the FKF1-LOV polypeptides [272, 273] were attributed to the additional nine-residue loop insertion between Eα near a conserved Cys and the Fα helix found in the ZEITLUPE family. A FKF1-LOV polypeptide lacking the loop insertion showed a faster recovery rate in the dark compared to the FKF1-LOV with the loop intact, where no conformational change was detected [272]. This could reflect the importance of the loop in conformational changes upon light excitation and light signal transduction. In phototropins, one of the two LOV domains (LOV1) is required for dimerization [274, 275], while LOV2 is solely involved in photoreceptor activity. The single LOV domain in FKF1-LOV forms stable dimers [267], suggesting that the LOV domains in the ZTL/FKF1/LKP2 family function both as photoreceptors for blue light signal transduction and mediators for protein–protein interactions [253]. Detailed crystallographic and spectroscopic studies of the light-activated full-length proteins and their complexes are necessary to understand these interactions and the functional mechanism of the LOV domains.

Cryptochromes (CRYs) are flavoproteins that show overall structural similarity to DNA repair enzymes known as DNA photolyases [276]. They were first identified in *Arabidopsis* where a CRY mutant showed abnormal growth and development in response to blue light [277]. In response to light, photolyases and cryptochromes use the same FAD cofactor to perform dissimilar functions; specifically, photolyases catalyze DNA repair, while CRYs tune the circadian clock in animals and control developmental processes in plants like photomorphogenesis and photoperiodic flowering [125, 278–281]. Cryptochromes can be classified in three subfamilies that include the two classic cryptochromes from plants and animals and a third cryptochrome subfamily called DASH (DASH for *Drosophila*, *Arabidopsis*, *Synechocystis*, *Homo sapiens*) [249] whose members are more closely related to photolyases then the classic cryptochromes. They bind DNA and their role in biological signaling remains unclear [247, 249].

Cryptochromes have 1) an N-terminal photolyase homology region (PHR) and 2) a variable C-terminal domain that contains the nuclear localization signal (absent in photolyase and CRY-DASH proteins and with no obvious sequence similarity to known protein domains). The PHR region can bind two different chromophores: FAD and pterin [125, 276, 281].

**Fig. 16:**
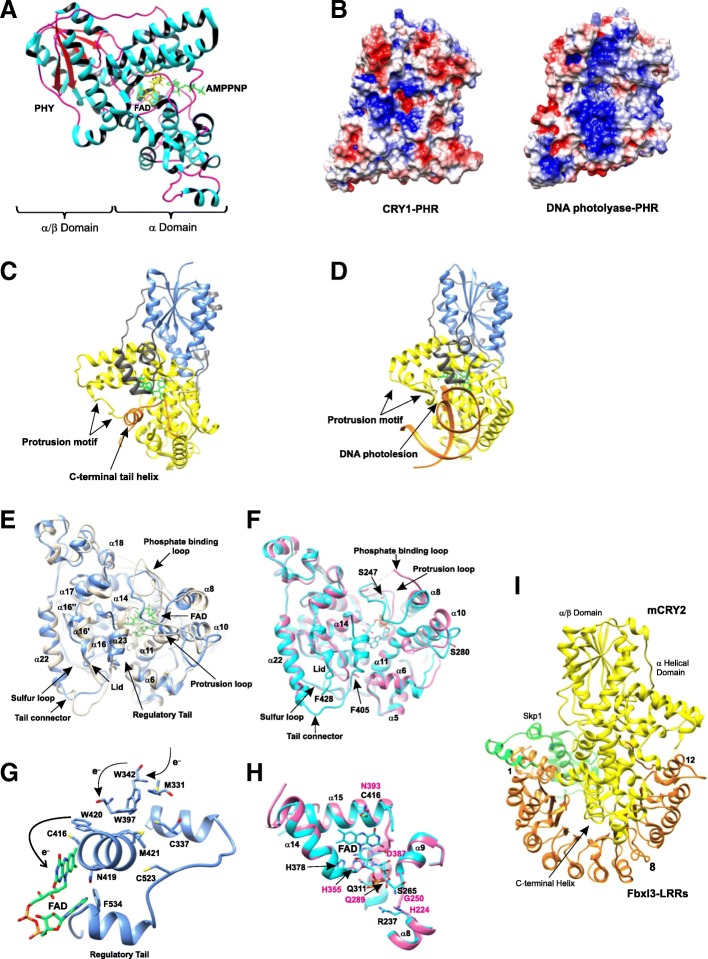
**a** CRY1-PHR structure (PDB 1U3D) with helices in *cyan*, β-strands in *red*, FAD cofactor in *yellow*, and AMPPNP (ATP analogue) in *green*. **b** electrostatic potential in CRY1-PHR and *E. coli* DNA photolyase (PDB 1DNP). Surface areas colored *red* and *blue* represent negative and positive electrostatic potential, respectively. **c** dCRY (PDB 4JZY) and **d** 6-4 dPL (PDB 3CVU). The C-terminal tail of dCRY (*orange*) replaces the DNA substrate in the DNA-binding cleft of dPL. The N-terminal α/β domain (*blue*) is connected to the C-terminal helical domain (*yellow*) through a linker (*gray*). FAD cofactor is in *green*. **e** Structural comparison of dCRY (*blue*; PDB 4JZY) with dCRY (*beige*; PDB 3TVS, initial structure; 4GU5, updated) [308, 309]. Significant changes are in the regulatory tail and adjacent loops. **f** Structural comparison of mCRY1 (*pink*; PDB 4K0R) with the dCRY (*cyan*; PDB 4JZY) regulatory tail and adjacent loops depicting the changes. Conserved Phe (Phe428_dCRY_ and Phe405_mCRY1_) depicted that facilitates C-terminal lid movement. **g** dCRY photoactivation mechanism: Trp342, Trp397, and Trp290 form the classic Trp eˉ transfer cascade. Structural analysis suggest the involvement of the eˉ rich sulfur loop (Met331 and Cys337), the tail connector loop (Cys523), and Cys416, which are in close proximity to the Trp cascade in the gating of eˉs via the cascade. **h** Comparison of the FAD binding pocket of dCRY (*cyan*) and mCRY1 (*pink*). Asp387_mCRY1_ occupies the binding pocket. The mCRY1 residues (His355 and Gln289), corresponding to His 378 and Gln311 in dCRY, at the pocket entrance are rotated to "clash" with the FAD moiety. Gly250_mCRY1_ and His224_mCRY1_ superimpose Ser265_dCRY_ and Arg237_dCRY_, respectively. **i** Crystal structure of the complex (PDB 4I6J) between mCRY2 (*yellow*), Fbxl3 (*orange*), and Skp1 (*green*). The numbers 1, 8, and 12 display the position of the respective leucine rich repeats (LRR) present in Fbxl3

In the absence of any high-resolution structure for a CRY protein, the functional analysis of this blue-light receptor was not clear earlier. Although the structure of CRY-DASH is known from *Synechocystis* [249], it does not clearly explain its role as a photoreceptor [282]. The crystal structure (Fig. 16a) of the PHR region of CRY1 (CRY1-PHR) from *Arabidopsis* [282], solved using the DNA photolyase PHR (PDB 1DNP) from a bacterial species as a molecular replacement probe [283–285], led to elucidation of the differences between the structure of photolyases and CRY1 and the clarification of the structural basis for the function of these two proteins. CRY1-PHR consists of an N-terminal α/β domain and a C-terminal α domain. The α/β domain consists of five parallel β-strands surrounded by four α-helices and a 3_10_-helix. The α domain is the FAD binding region and consists of fourteen α-helices and two 3_10_-helices. The two domains are linked by a helical connector comprised of 77 residues. FAD binds to CRY1-PHR in a U-shaped conformation and is buried deep in a cavity formed by the α domain [282]. In contrast to photolyases, which have a positively charged groove near the FAD cavity for docking of the dsDNA substrate [283], the CRY1-PHR structure reveals a negatively charged surface with a small positive charge near the FAD cavity (Fig. 16b), strongly suggesting the absence of DNA-binding activity. Trp277 and Trp324 in bacterial photolyases are important for thymine-dimer binding and DNA binding [283–285]. In CRY1-PHR, they are replaced by Leu296 and Tyr402. These differences, combined with a larger FAD cavity and unique chemical environment in CRY1-PHR created by different amino acid residues and charge distribution [282], explain the different functions of the two proteins. Still, the mechanism of the blue-light signaling by CRYs is not completely clear. The CRY1-PHR structure lacks the C-terminal domain of the full-length CRY1 that is crucial in the interaction with proteins downstream in the blue-light signaling pathway [286, 287]. CRY1 and CRY2 regulate COP1, an E3 ubiquitin ligase, through direct interaction via the C-terminus. Also, β-glucuronidase (GUS) fused CCT1/CCT2 expression in *Arabidopsis* mediates a constitutive light response [286, 287]. However, a recent study has shown N-terminal domain (CNT1) constructs of *Arabidopsis* CRY1 to be functional and to mediate blue light-dependent inhibition of hypocotyl elongation even in the absence of CCT1 [288]. Another study has identified potential CNT1 interacting proteins: CIB1 (cryptochrome interacting basic helix-loop-helix1) and its homolog, HBI1 (HOMOLOG OF BEE2 INTERACTING WITH IBH 1) [289]. The two proteins promote hypocotyl elongation in *Arabidopsis* [290–292]*.* The study showed HBI1 acts downstream of CRYs and CRY1 interacts directly with HBI1 through its N-terminus in a blue-light dependent manner to regulate its transcriptional activity and hence the hypocotyl elongation [289]. Previous studies have shown that the CRY2 N-terminus interaction with CIB1 regulates the transcriptional activity CIB1 and floral initiation in *Arabidopsis* in a blue light-dependent manner [293]. These studies suggest new/alternative mechanisms of blue-light-mediated signaling pathways for CRY1/2 independent of CCTs.

### Insects and mammals

Identification of the cryptochromes in plants subsequently led to their identification in *Drosophila* and mammals. Interestingly, studies have shown that *cry* genes, both in *Drosophila* and mammals, regulate the circadian clock in a light-dependent [123–125] and light-independent manner [126, 127]. An isolated *cry*^*b*^ mutant [294] in *Drosophila* did not respond to brief light impulses under constant darkness, whereas overexpressing wild-type *cry* caused hypersensitivity to light-induced phase shifts [124]. Light signal transduction in *Drosophila* is mediated through light-dependent degradation of TIM. Light-activated CRY undergoes a conformational change that allows it to migrate to the nucleus where it binds to the dPER–dTIM complex, thus inhibiting its repressive action [295]. dCRY blocking leads to phosphorylation of the complex and subsequent degradation by the ubiquitin-proteasome pathway [296]. However, flies lacking CRY could still be synchronized, suggesting the presence of other photoreceptors. Light input to the *Drosophila* clock can also occur via compound eyes, as external photoreceptors and Hofbauer-Buchner eyelets behind the compound eyes, where rhodopsin is present as the main photoreceptor [297–300]. CRY-mediated input signals occur through lateral neurons and dorsal neurons in the brain, which function as internal photoreceptors [301]. In the case of external photoreceptors, the downstream signaling pathway that leads to TIM degradation is not clear. However, lack of both external and internal photoreceptors completely abolished photoentrainment in *Drosophila* [302]*.*

The C-terminal extensions that are characteristic of CRYs in the Cryptochrome/Photolyase family gained considerable attention owing to their crucial role in various cryptochrome functions (reviewed in [125, 247, 281]). Despite the high similarity of the PHR regions among the CRYs in a given kingdom, the C-terminal extensions are variable in sequence, as well as in size. In plants, the C-terminal extension has three conserved motifs that are collectively referred to as DAS motifs and are comprised of DQXVP in the N-terminal end of the C-terminal extension, a region made up of acidic residues (E or D) and a STAES region followed by GGXVP at the C-terminal end of the extension [246]. A nuclear-localization domain is present in the C-terminal domain of plants and is required for function. In animals, the cryptochromes have been categorized into two types: one that acts as circadian photoreceptors (in insects) and another that acts as light-independent transcriptional repressors that function as integral components of the circadian clock (in vertebrates). Their functional diversity is attributed to the C-terminal extension. Various genetic and biochemical studies have reflected the importance of the C-terminal extension in subcellular localization, protein–protein interaction, and cryptochrome degradation via a proteasome-dependent pathway. The C-terminal extension is sufficient for nucleocytoplasmic trafficking of CRYs. Reports on *Arabidopsis* and *Drosophila* cryptochromes showed that the presence of both the PHR domain and C-terminal extension is essential to cryptochrome-mediated functions. However, like a functional N-terminal domain of *Arabidopsis* CRYs independent of the CCTs, studies on N-terminal domain constructs lacking the C-terminal domain of *Drosophila* CRY demonstrate it to be functional. A *Drosophila cry* mutant allele (*cry*^m^) expressing only the N-terminal CRY domain was observed to be capable of light detection and photoransduction independent of the C-terminus [303]. Also, transgenic *Drosophila* lines overexpressing CRYΔ lacking the C-terminus resulted in a constituively active form that did not degrade [304]. CRYs undergo a blue light-dependent conformational change, making the C-terminal extension available for protein–protein interaction with downstream signaling partners, subsequently leading to CRY/CRY-mediated degradation.

Studies report direct interaction between CRY and COP1/phyB/ZTL/LKP1/ADO1 in plants, and mPER in animals, mediated via the C-terminus. Studies of chimeric proteins made by fusion of *Arabidopsis* (6-4) photolyase-PHR-CRY1-CCT domains showed that the features of both domains are obligatory for the repressive action of the CRY protein. The C-terminus is not sufficient to mediate the transcriptional repressor function [125, 247, 281]. In *Drosophila*, the C-terminal extension has been shown to be critical to the role of dCRY as a magnetoreceptor [305, 306]. Many organisms have a magnetosensing ability, utilizing the Earth’s magnetic field for navigation and orientation [247]. Lack of the dCRY C-terminus disrupts the electromagnetic field-sensing abilty of CRY, thus affecting the negative geotaxis ability of *Drosophila* [305, 306]. The *Drosophila* clock showed increasingly slow rhythms in response to an applied magnetic field in the presence of blue light. The magnetosensitivity was also affected by the field strength. *cry* mutants with an impaired FAD or mutants lacking cry were observed to be unresponsive to the applied magnetic field. *Drosophila* clock neurons overexpressing CRYs showed robust sensitivity to an applied field [306, 307].

Structural studies on the animal cryptochromes contributed immensely to the understanding of their function. Structures have been solved for both full length and truncated CRYs (*Drosophila* and mammalian) and show overall similarities. There are, however, significant differences and these are implicated in defining their diverse functions [308–311]. A full-length dCRY structure (3TVS) by Zoltowski et al. [308] includes the variable C-terminal tail (CTT) attached to the photolyase homology region. The dCRY structure, excluding the intact C-terminal domain, resembles (6-4) photolyases, with significant differences in the loop structures, antenna cofactor-binding site, FAD center, and C-terminal extension connecting to the CTT. The CTT tail mimics the DNA substrates of photolyases [308]. This structure of dCRY was subsequently improved (PDB 4GU5) [309] and another structure (PDB 4JY) was reported by Czarna et al. [310] (Fig. 16c, d), which together showed that the regulatory CTT and the adjacant loops are functionally important regions (Fig. 16e). As a result, it now appears that the conserved Phe534 is the residue that extends into the CRY catalytic center, mimicking the 6-4 DNA photolesions. Together it was shown that CTT is surrounded by the protrusion loop, the phosphate binding loop, the loop between α5 and α6, the C-terminal lid, and the electron-rich sulfur loop [310].

The structure of animal CRY did not reveal any cofactor other than FAD. In CRYs, flavin can exist in two forms: the oxidized FAD^ox^ form or as anionic semiquinone FAD^°-^. During photoactivation, dCRY changes to the FAD^°-^ form, while photolyases can form neutral semiquinone (FADH^°^). Unlike photolyases, where an Asn residue can only interact with the protonated N5 atom, the corresponding Cys416 residue of dCRY readily forms a hydrogen bond with unprotonated N5 and O4 of FAD, thus stabilizing the negative charge and preventing further activation to FADH.-, which is the form required for DNA repair in photolyases [308]. Structural analysis and the mutational studies of dCRY have defined the tail regions as important for FAD photoreaction and phototransduction to the tail (Fig. 11g). The residues in the electron-rich sulfur loop (Met331 and Cys337) and Cys523 in the tail connector loop, owing to their close proximity to the classic tryptophan electron transport cascade (formed by Trp420, Trp397and Trp342), influence the FAD photoreaction and play an important role in determining the lifetime of FAD^°-^ formation and decay and regulating the dynamics of the light-induced tail opening and closing. Additionally Phe534, Glu530 (tail helix), and Ser526 (connector loop) stabilize the tail interaction with the PHR in the dark-adapted state [310]. These are important structural features that determine why these CRYs now lack photolyase activity.

The structure of the apo-form of mCRY1 by Czarna et al. [310] shows an overall fold similar to dCRY and (6-4) photolyase. Differences are observed in the extended loop between the α6 and α8 helices, which was found to be partially disordered and structurally different when compared to that in dCRY. Conformational differences (Fig. 11f) are also observed in the protrusion loops (seven residues shorter in mCRY1 and consists of Ser280: the AMPK phosphorylation site), the phosphate-binding loop (structurally different than in dCRY and partly disordered), and the C-terminal lid, which was unstructured. The lid forms the wall between the FAD binding pocket, the predicted coiled-coil helix α22, and the sulfur loop [310]. Helix α11 (Tyr287/Gly288), following the protrusion loop, has been shown before to be important for the repressive action of mCRY1 [312, 313]. Comparison of dCRY and apo mCRY1 did not show drastic changes in the FAD binding pocket (Fig. 16h). Highly conserved Asp and Arg residues form a salt bridge contributing to the stability of FAD binding in all the known CRYs/photolyases, and are moved inside the FAD binding pocket in mCRY1 [310].

Structural analysis [310] of the previously reported mutational studies [314] depict the C-terminal α22 helix and C-terminal mCRY1 tail to be essential for the transcriptional repression function of mCRY1 and the interaction with other core clock proteins. Analysis of various mutants of mCRY1 with mutations in the C-terminal lid (F504A), the predicted coiled-coil α22 helix (K485D/E, G336D), charged surfaces, the FAD-binding pocket (H355E, H224E), and the phosphate binding loop (S247D) suggested that even though common regions are utilized in the interaction with mPER2, FBXL3, and mCLOCK/BMAL1, the mode of interaction differs. The mCRY1 α22 helix and the acidic region play an essential role in the transcriptional repression function as well as protein–protein interactions. In addition, the C-terminal lid and the basic and acidic surface regions near the FAD binding pocket regulate mPER2, FBXL3, and mCLOCK/BMAL1 binding [310].

The crystal structure of the apo form of mCRY2-PHR [311] showed a photolyase-like fold. The crystal structure of the FAD-bound mCRY2-PHR showed that it retains the FAD binding activity (K_d_ ~ 40 μm). FAD adopted a similar U-shaped conformation as observed in other CRYs and photolyases. However, it was reported that the FAD moiety is only partially buried in the binding pocket [311]. In (6-4) photolyases FAD was found to be deep inside the pocket hidden under a well-ordered phosphate binding loop and a nearby protrusion motif. The central lysine of the phosphate binding loop forms a hydrogen bond with the N7 atom of the adenine on the cofactor [282, 312]. In contrast, the phosphate binding loop is completely disordered and the protrusion motif is moved away from FAD in FAD bound mCRY2-PHR [311].

mCRY2 with an intact CCT was found to form a stable complex with Fbxl3–Skpl [311]. A crystal structure determined for the heterotrimerc complex mCRY2–Fbxl3–Skpl (Fig. 16i) shows a globular mCRY2 fitted on a cup-shaped Fbxl3–Skpl complex via its α-helical domain. Fbxl3 consists of a three-helix F-box motif, a C-terminal leucine rich repeat (LRR) domain that contains 12 LRRs, followed by a 12 amino acid residue-long C-terminal tail (conserved in vertebrates) that ends with a Trp. The LRR domain forms a semicircular solenoid structure with parallel β-strands on its concave surface and α-helical coils on its convex surface. Based on the structural irregularities of LRR7 and 8 (longer β-strands compared to the others) the LRR domain could be divided into two halves: LRR-N (1–6) and LRR-C (7–12). The mCRY2-Fbxl3 interaction interface analysis showed a closer contact between Fbxl3 LRR-C and the mCRY2 α-helical domain. The Fbxl3 C-terminal tail caps the solenoid structure and enters into the α-helical domain of mCRY2. The terminal Trp occupies the core of the FAD-binding pocket similar to the (6-4) DNA lesion in the d(6-4)photolyase–DNA complex structure. The interface was observed to be highly hydrophobic and revealed a large surface adjacent to the cofactor binding pocket on mCRY2. This surface is formed by three structural motifs: the interface loop, the C-terminal helix, and the 11 amino acid-long conserved segment (CSS) preceding the C-terminal tail. Binding activity analysis of various Fbxl3 and mCRY2 mutants showed that complex formation is significantly affected by mutations in the Fbxl3 tail and the mCRY2 cofactor pocket [311].

The phosphorylation sites at Ser71 and Ser280 alter mCRY stability [315] and thus its binding affinity to its protein partners by restructuring the local environment. The addition of free FAD disrupted the complex between Fbxl3-mCRY2 suggesting an antagonistic role in regulating Fbxl3–mCRY2 interaction [311]. The C-terminal helix of mCRY2 is essential for PER binding [247], which is masked by the LRR domain in the mCRY2–Fbxl3–Skp1 complex [311]. All these suggest that PER abundance and the metabolic state inside the cell regulate CRY stability and ultimately the clock rhythmicity. Such knowledge can guide the design of compounds that influence CRY stability and hence was proposed as a strategy for treating metabolic anomalies [316–318].

Light input in mammals occurs via eyes and reaches the retina, from which signals for clock entrainment are sent to the pacemaker SCN. Circadian rhythms can be entrained in mice lacking classic visual photoreceptors (rods and cones), but not in enucleated mice, suggesting that nonvisual photoreceptors could play a role in photoentrainment of the mammalian circadian clock [319, 320]. Studies showed that a subset of intrinsically photosensitive retinal ganglion cells (ipRGCs) located in the inner nuclear layer of the retina are responsible for circadian light resetting. The ipRGCs form a retinohypothalamic tract (RHT) that projects into the pacemaker SCN. Lesion of the RHT resulted in the inability of circadian responses to light [319, 320].

Melanopsin (Opn4), a new opsin molecule that has emerged over the past decade as a potential photoreceptor for photoentrainment, is enriched in the ipRGCs [321, 322]. Mice lacking melanospin (*Opn4*^*-/-*^) showed less sensitivity to brief light perturbations under DD [323]. However, the phase and period responses in the *Opn4*^*-/-*^ mice were not completely absent, indicating the involvement of other photoreceptors in the entrainment process. mCRY1 and mCRY2 are found in the inner layer of the retina [313]. Also, hCRY1 expressed in living Sf21 insect cells showed photoconversion similar to that observed in plant and *Drosophila* cryptochromes upon light irradiation, suggesting a possible role as photoreceptors in mammals [324, 325]. However, the role of mammalian cryptochromes in photoreception is complicated by the fact that they are a crucial part of the core oscillator machinery. Gene knockout results in an arrhythmic clock, thus making it difficult to assay its role as a photoreceptor [126, 127]. Work by Dkhissi-Benyahya et al. [326] demonstrated that with changing light intensity, mammals recruit multiple photoreceptor systems to entrain the clock in a wavelength-dependent manner. They discovered the role of medium wavelength opsin (MW-opsin, located in the outer retina) in photoentrainment, in addition to melanopsin [326]. Thus, light entrainment in mammals is like other organisms, such as insects and plants, where existence of multiple photoreceptors helps the organism to adapt to the diurnal changes in light intensity and wavelength to synchronize the circadian rhythms. Several downstream light signaling pathways have been described for transmitting light to the circadian clock [321, 322]. RHT consists of glutamate and the pituitary adenylate cyclase-activating polypeptide (PACAP), the key putative neurotransmitters of RHT that are responsible for signal transduction to the SCN that ultimately drives the induction of the Per genes [319, 320]. In addition to RHT, other neuronal inputs to the SCN have been identified. However, that is beyond the scope of this review.

## Summary

An exciting chapter of circadian clock research, which is focused on structural aspects, has brought with it new challenges. Whereas the structural aspects of the circadian clockwork in prokaryotes are relatively well studied, the picture regarding eukaryotic CCs is fragmentary, trivial, and far from complete. Much is to be done. A targeted protein complex, which is a structural feature common to all the clocks, has recently gained center-stage in bench science. Multimeric protein complex formations have been shown to be important for the regulation of several core oscillators. We know that the proteins contain identical conserved domains with their typical folds. However, structural analysis of the CLOCK–BMAL1 complex and the PERIOD homodimers suggests that the dynamics of the assembly and disassembly of hetero-multimeric protein complexes is dependent on the differential spatial arrangement of the domains. Additionally, the CLOCK/BMAL1 proteins show potential for a differential electrostatic surface that endowes the complex with asymmetry, indicating that differential surface potential might be responsible for the disparity in their interaction with PER/CRY and, hence, for distinct functions.

Sequential phosphorylation is another feature that influences protein–protein interactions in circadian clocks. The dynamics of the cyanobacterial KaiC phosphorylation cycle have been observed to be driven by regulated cycles of interaction with KaiA and KaiB that trigger the enzymatic switch in KaiC. However, both the precise time point for the switch and an understanding of how the information relayed between the phosphorylation/dephosphorylation event and the physical protein–protein interaction triggers the switch are issues that remain to be elucidated. Sequential phosphorylation has also been observed in the eukaryotic clock. Protein–protein and/or protein–DNA interactions coupled with progressive phosphorylation and dephosphorylation events have been shown to be important for stability, subcellular distribution, and the function of the core-clock components [4, 48, 51, 150, 165]. PER-mediated inhibition of dCLK/dCYC activity involves association with DOUBLETIME (DBT), a kinase. DBT phosphorylates CLK, resulting in its inhibition and degradation [327]. Similarly, in *Neurospora*, FRQ interaction with FRH and kinases results in WCC phosphorylation, thus repressing its activity [97, 104]. CCA1 and TOC1 function and stability are also subject to phosphorylation regulation [165, 328]. However, it is not clear which event, phosphorylation or oligomerization, occurs first such that nuclear accumulation and activity result. Phosphorylation of the *Drosophila* CLK protein is not only sequential, but is also compartment-specific. Although phosphorylation of FRQ is crucial for its transcriptional repression activity, Cha et al. [51] showed that it is not important for the regulation of the cellular distribution of FRQ. Future structural studies of these proteins individually and in complex assemblies will provide the mechanistic details with which to understand the dynamics of these events.

The dynamics of phosphorylation and dephosphorylation are also important for the transmission of external environmental cues and for resetting the clock. A light-dependent conformational change of the photoreceptors directs a downstream cascade of phosphorylation and protein–protein interactions that defines the period length and the phase shifts. Another interesting mechanism of clock resetting has been observed in the cyanobacterial clock, where the metabolic state of the cell entrains the clock in a light-dependent manner. Circadian metabolic rhythms are also observed in higher organisms [329]. Feeding can entrain the circadian clock in rat liver independent of synchronization with the SCN or light cycle [330]. The nutritional status of the organism drives adenosine monophosphate-activated protein kinase-mediated phosphorylation of cryptochromes and entrains the peripheral clocks [331]. However, the mechanism of entrainment is not clear. Structural analysis of the CRY proteins depicts how phosphorylation and the metabolic state of the cell direct its interaction with different protein partners that regulate CRY stability and function. The extended overlapping binding interface for PER and Fbxl3 prevents them from interacting simultaneously. Interaction of Fbxl3 with CRY requires the binding of the Fbxl3 tail to the FAD binding pocket in CRY. One small molecule (Kloo1; a carbazole derivative) can modulate circadian period by interacting directly with CRY at its FAD binding pocket and protecting CRY from SCF^Fbxl3^-mediated ubiquitination. The crystal structure of the mCRY2 PHR–Kloo1 complex shows that Kloo1 is buried deep in the pocket and mimics the cofactor [332].

The cyanobacterial CC is an enzymatic clock wherein KaiC, central to the clock, exhibits all the enzymatic activities. The eukaryotic circadian system is, instead, a complex network of transcription factors, regulatory proteins, kinases, and phosphatases. The common elements in the CC systems in different kingdoms of life are fairly well known. However, notwithstanding the coarse models we have, enough differences have been brought about by the different evolutionary paths and different environmental adaptations to justify detailed studies of CCs in different organisms. From this perspective, the efforts invested by us and others, especially with regard to the structural dissection of the circadian systems, are timely and well placed.

## References

[1] Johnson CH, Egli M, Stewart PL (2008). Structural insights into a circadian oscillator. Science..

[2] Heintzen C, Liu Y (2007). The Neurospora crassa circadian clock. Adv Genet..

[3] Harmer SL (2009). The circadian system in higher plants. Annu Rev Plant Biol..

[4] Dunlap JC (1999). Molecular bases for circadian clocks. Cell..

[5] Wijnen H, Young MW (2006). Interplay of circadian clocks and metabolic rhythms. Annu Rev Genet..

[6] Young MW, Kay SA (2001). Time zones: a comparative genetics of circadian clocks. Nat Rev Genet..

[7] Johnson CH (2001). Endogenous timekeepers in photosynthetic organisms. Annu Rev Physiol..

[8] Sawyer LA, Hennessy JM, Peixoto AA, Rosato E, Parkinson H, Costa R, Kyriacou CP (1997). Natural variation in a Drosophila clock gene and temperature compensation. Science..

[9] Gould PD, Locke JC, Larue C, Southern MM, Davis SJ, Hanano S, Moyle R, Milich R, Putterill J, Millar AJ, Hall A (2006). The molecular basis of temperature compensation in the Arabidopsis circadian clock. Plant Cell..

[10] Diernfellner A, Colot HV, Dintsis O, Loros JJ, Dunlap JC, Brunner M (2007). Long and short isoforms of Neurospora clock protein FRQ support temperature-compensated circadian rhythms. FEBS Lett..

[11] Mihalcescu I, Hsing W, Leibler S (2004). Resilient circadian oscillator revealed in individual cyanobacteria. Nature..

[12] Nagoshi E, Saini C, Bauer C, Laroche T, Naef F, Schibler U (2004). Circadian gene expression in individual fibroblasts: cell-autonomous and self-sustained oscillators pass time to daughter cells. Cell..

[13] Welsh DK, Yoo SH, Liu AC, Takahashi JS, Kay SA (2004). Bioluminescence imaging of individual fibroblasts reveals persistent, independently phased circadian rhythms of clock gene expression. Curr Biol..

[14] Loros JJ, Dunlap JC, Larrondo LF, Shi M, Belden WJ, Gooch VD, Chen C-H, Baker CL, Mehra A, Colot HV, Schwerdtfeger C, Lambreghts R, Collopy PD, Gamsby JJ, Hong CI (2007). Circadian output, input, and intracellular oscillators: insights into the circadian systems of single cells. Cold Spring Harb Symp Quant Biol..

[15] Cheng P, Yang Y, Liu Y (2001). Interlocked feedback loops contribute to the robustness of the Neurospora circadian clock. Proc Natl Acad Sci U S A..

[16] Brunner M, Káldi K (2008). Interlocked feedback loops of the circadian clock of Neurospora crassa. Mol Microbiol..

[17] Shearman LP, Sriram S, Weaver DR, Maywood ES, Chaves I, Zheng B, Kume K, Lee CC, van der Horst GT, Hastings MH, Reppert SM (2000). Interacting molecular loops in the mammalian circadian clock. Science..

[18] Liu Y, Tsinoremas NF, Johnson CH, Lebedeva NV, Golden SS, Ishiura M, Kondo T (1995). Circadian orchestration of gene expression in cyanobacteria. Genes Dev..

[19] Nakahira Y, Katayama M, Miyashita H, Kutsuna S, Iwasaki H, Oyama T, Kondo T (2004). Global gene repression by KaiC as a master process of prokaryotic circadian system. Proc Natl Acad Sci U S A..

[20] Vitalini MW, de Paula RM, Park WD, Bell-Pedersen D (2006). The rhythms of life: circadian output pathways in Neurospora. J Biol Rhythms..

[21] Greene AV, Keller N, Haas H, Bell-Pedersen D (2003). A circadian oscillator in Aspergillus spp. regulates daily development and gene expression. Eukaryot Cell..

[22] Galagan JE, Calvo SE, Cuomo C, Ma LJ, Wortman JR, Batzoglou S, Lee SI, Basturkmen M, Spevak CC, Clutterbuck J, Kapitonov V, Jurka J, Scazzocchio C, Farman M, Butler J, Purcell S, Harris S, Braus GH, Draht O, Busch S, D'Enfert C, Bouchier C, Goldman GH, Bell-Pedersen D, Griffiths-Jones S, Doonan JH, Yu J, Vienken K, Pain A, Freitag M, Selker EU, Archer DB, Penalva MA, Oakley BR, Momany M, Tanaka T, Kumagai T, Asai K, Machida M, Nierman WC, Denning DW, Caddick M, Hynes M, Paoletti M, Fischer R, Miller B, Dyer P, Sachs MS, Osmani SA, Birren BW (2005). Sequencing of Aspergillus nidulans and comparative analysis with A. fumigatus and A. oryzae. Nature..

[23] Young MW (2000). The tick-tock of the biological clock. Sci Am..

[24] Panda S, Hogenesch JB, Kay SA (2002). Circadian rhythms from flies to human. Nature..

[25] Reppert SM, Weaver DR (2002). Coordination of circadian timing in mammals. Nature..

[26] Harmer SL, Hogenesch JB, StraumeM CHS, Han B, Zhu T, Wang X, Kreps JA, Kay SA (2000). Orchestrated transcriptionof key pathways in Arabidopsis by the circadian clock. Science.

[27] Yakir E, Hilman D, Harir Y, Green RM (2007). Regulation of output from the plant circadian clock. FEBS J..

[28] Michael TP, Mockler TC, Breton G, McEntee C, Byer A, Trout JD, Hazen SP, Shen R, Priest HD, Sullivan CM, Givan SA, Yanovsky M, Hong F, Kay SA, Chory J (2008). Network discovery pipeline elucidates conserved time-of-day-specific cis-regulatory modules. PLoS Genet..

[29] Robertson FC, Skeffington AW, Gardner MJ, Webb AA (2009). Interactions between circadian and hormonal signalling in plants. Plant Mol Biol..

[30] Lakin-Thomas PL, Brody S (2004). Circadian rhythms in microorganisms: new complexities. Annu Rev Microbiol..

[31] Bell-Pedersen D, Cassone VM, Earnest DJ, Golden SS, Hardin PE, Thomas TL, Zoran MJ (2005). Circadian rhythms from multiple oscillators: lessons from diverse organisms. Nat Rev Genet..

[32] Egli M, Stewart PL (2009). Structural aspects of the cyanobacterial KaiABC circadian clock. Bacterial Circadian Programs.

[33] Edgar RS, Green EW, Zhao Y, Van Ooijen G, Olmedo M, Qin X, Xu Y, Pan M, Valekunja UK, Feeney KA, Maywood ES, Hastings MH, Baliga NS, Merrow M, Millar AJ, Johnson CH, Kyriacou CP, O'Neill JS, Reddy AB (2012). Peroxiredoxins are conserved markers of circadian rhythms. Nature..

[34] Drucker-Colin R, Aguilar-Roblero R, Garcia-Hernandez F, Fernandez-Cancino F, Rattoni FB (1984). Fetal suprachiasmatic nucleus transplants: diurnal rhythm recovery of lesioned rats. Brain Res..

[35] Ralph MR, Foster RG, Davis FC, Menaker M (1990). Transplanted suprachiasmatic nucleus determines circadian period. Science..

[36] Yamazaki S, Numano R, Abe M, Hida A, Takahashi R, Ueda M, Block GD, Sakaki Y, Menaker M, Tei H (2000). Resetting central and peripheral circadian oscillators in transgenic rats. Science..

[37] Zylka MJ, Shearman LP, Weaver DR, Reppert SM (1998). Three period homologs in mammals: differential light responses in the suprachiasmatic circadian clock and oscillating transcripts outside of brain. Neuron..

[38] Plautz JD, Kaneko M, Hall JC, Kay SA (1997). Independent photoreceptive circadian clocks throughout Drosophila. Science..

[39] Myers EM, Yu J, Sehgal A (2003). Circadian control of eclosion: interaction between a central and peripheral clock in Drosophila melanogaster. Curr Biol.

[40] Peng Y, Stoleru D, Levine JD, Hall JC, Rosbash M (2003). Drosophila free-running rhythms require intercellular communication. PLoS Biol..

[41] Stoleru D, Peng Y, Agosto J, Rosbash M (2004). Coupled oscillators control morning and evening locomotor behaviour of Drosophila. Nature..

[42] Grima B, Chelot E, Xia R, Rouyer F (2004). Morning and evening peaks of activity rely on different clock neurons of the Drosophila brain. Nature..

[43] Rieger D, Shafer OT, Tomioka K, Helfrich-Forster C (2006). Functional analysis of circadian pacemaker neurons in Drosophila melanogaster. J Neurosci..

[44] Aschoff J (1960). Exogenous and endogenous components in circadian rhythms. Cold Spring Harb Symp Quant Biol..

[45] Johnson CH. Phase response curves: What can they tell us about circadian clocks? In: Hiroshige T, Honma K, editors. Circadian clocks from cell to human. Sapporo; Hokkaido University Press: 1992. p. 209–249.

[46] Hardin PE (2005). The circadian timekeeping system of Drosophila. Curr Biol..

[47] Guo J, Liu Y (2010). Molecular mechanism of the Neurospora circadian oscillator. Protein Cell..

[48] Lu SX, Knowles SM, Andronis C, Ong MS, Tobin EM (2009). CIRCADIAN CLOCK ASSOCIATED1 and LATE ELONGATED HYPOCOTYL function synergistically in the circadian clock of Arabidopsis. Plant Physiol..

[49] Hennig S, Strauss HM, Vanselow K, Yildiz Ö, Schulze S, Arens J, Kramer A, Wolf E (2009). Structural and functional analyses of PAS domain interactions of the clock proteins Drosophila PERIOD and mouse PERIOD2. PLoS Biol..

[50] Hung HC, Maurer C, Zorn D, Chang WL, Weber F (2009). Sequential and compartment-specific phosphorylation controls the life cycle of the circadian CLOCK protein. J Biol Chem..

[51] Cha J, Yuan H, Liu Y (2011). Regulation of the activity and cellular localization of the circadian clock protein FRQ. J Biol Chem..

[52] Kucera N, Schmalen I, Hennig S, Öllinger R, Strauss HM, Grudziecki A, Wieczorek C, Kramer A, Wolf E (2012). Unwinding the differences of the mammalian PERIOD clock proteins from crystal structure to cellular function. Proc Natl Acad Sci U S A..

[53] Ishiura M, Kutsuna S, Aoki S, Iwasaki H, Andersson CR, Tanabe A, Golden SS, Johnson CH, Kondo T (1998). Expression of a gene cluster kaiABC as a circadian feedback process in cyanobacteria. Science..

[54] Nakajima M, Imai K, Ito H, Nishiwaki T, Murayama Y, Iwasaki H, Oyama T, Kondo T (2005). Reconstitution of circadian oscillation of cyanobacterial KaiC phosphorylation in vitro. Science..

[55] Tomita J, Nakajima M, Kondo T, Iwasaki H (2005). Circadian rhythm of KaiC phosphorylation without transcription-translation feedback. Science..

[56] Kitayama Y, Nishiwaki T, Terauchi K, Kondo T (2008). Dual KaiC-based oscillations constitute the circadian system of Cyanobacteria. Genes Dev..

[57] Hayashi F, Suzuki H, Iwase R, Uzumaki T, Miyake A, Shen J-R, Imada K, Furukawa Y, Yonekura K, Namba K, Ishiura M (2003). ATP-induced hexameric ring structure of the cyanobacterial circadian clock protein KaiC. Genes Cells..

[58] Pattanayek R, Wang J, Mori T, Xu Y, Johnson CH, Egli M (2004). Visualizing a circadian clock protein: crystal structure of KaiC and functional insights. Mol Cell..

[59] Garces RG, Wu N, Gillon W, Pai EF (2004). Anabaena circadian clock proteins KaiA and KaiB reveal potential common binding site to their partner KaiC. EMBO J..

[60] Ye S, Vakonakis I, Ioerger TR, LiWang AC, Sacchettini JC (2004). Crystal structure of circadian clock protein KaiA from Synechococcus elongatus. J Biol Chem..

[61] Uzumaki T, Fujita M, Nakatsu T, Hayashi F, Shibata H, Itoh N, Kato H, Ishiura M (2004). Crystal structure of the C-terminal clock-oscillator domain of the cyanobacterial KaiA protein. Nat Struct Mol Biol..

[62] Vakonakis I, LiWang AC (2004). Structure of the C-terminal domain of the clock protein KaiA in complex with a KaiC-derived peptide: implications for KaiC regulation. Proc Natl Acad Sci U S A..

[63] Hitomi K, Oyama T, Han S, Arvai AS, Getzoff ED (2005). Tetrameric architecture of the circadian clock protein KaiB: a novel interface for intermolecular interactions and its impact on the circadian rhythm. J Biol Chem..

[64] Iwase R, Imada K, Hayashi F, Uzumaki T, Morishita M, Onai K, Furukawa Y, Namba K, Ishiura M (2005). Functionally important substructures of circadian clock protein KaiB in a unique tetramer complex. J Biol Chem..

[65] Nishiwaki T, Iwasaki H, Ishiura M, Kondo T (2000). Nucleotide binding and autophosphorylation of the clock protein KaiC as a circadian timing process of cyanobacteria. Proc Natl Acad Sci U S A..

[66] Iwasaki H, Nishiwaki T, Kitayama Y, Nakajima M, Kondo T (2002). KaiA-stimulated KaiC phosphorylation in circadian timing loops in cyanobacteria. Proc Natl Acad Sci U S A..

[67] Xu Y, Mori T, Johnson CH (2003). Cyanobacterial circadian clockwork: roles of KaiA, KaiB, and the kaiBC promoter in regulating KaiC. EMBO J..

[68] Xu Y, Mori T, Pattanayek R, Pattanayek S, Egli M, Johnson CH (2004). Identification of key phosphorylation sites in the circadian clock protein KaiC by crystallographic and mutagenetic analyses. Proc Natl Acad Sci U S A..

[69] Nishiwaki T, Satomi Y, Nakajima M, Lee C, Kiyohara R, Kageyama H, Kitayama Y, Temamoto M, Yamaguchi A, Hijikata A, Go M, Iwasaki H, Takao T, Kondo T (2004). Role of KaiC phosphorylation in the circadian clock system of Synechococcus elongatus PCC 7942. Proc Natl Acad Sci U S A..

[70] Nishiwaki T, Satomi Y, Kitayama Y, Terauchi K, Kiyohara R, Takao T, Kondo T (2007). A sequential program of dual phosphorylation of KaiC as a basis for circadian rhythm in cyanobacteria. EMBO J..

[71] Rust MJ, Markson JS, Lane WS, Fisher DS, O'Shea EK (2007). Ordered phosphorylation governs oscillation of a three-protein circadian clock. Science..

[72] Xu Y, Mori T, Qin X, Yan H, Egli M, Johnson CH (2009). Intramolecular regulation of phosphorylation status of the circadian clock protein KaiC. PLoS One..

[73] Pattanayek R, Mori T, Xu Y, Pattanayek S, Johnson CH, Egli M (2009). Structures of KaiC circadian clock mutant proteins: a new phosphorylation site at T426 and mechanisms of kinase, ATPase and phosphatase. PLoS One..

[74] Phong C, Markson JS, Wilhoite CM, Rust MJ (2013). Robust and tunable circadian rhythms from differentially sensitive catalytic domains. Proc Natl Acad Sci U S A..

[75] Tseng R, Goularte NF, Chavan A, Luu J, Cohen SE, Chang YG, Heisler J, Li S, Michael AK, Tripathi S, Golden SS, LiWang A, Partch CL (2017). Structural basis of the day-night transition in a bacterial circadian clock. Science..

[76] Terauchi K, Kitayama Y, Nishiwaki T, Miwa K, Murayama Y, Oyama T, Kondo T (2007). ATPase activity of KaiC determines the basic timing for circadian clock of cyanobacteria. Proc Natl Acad Sci U S A..

[77] Nishiwaki-Ohkawa T, Kitayama Y, Ochiai E, Kondo T (2014). Exchange of ADP with ATP in the CII ATPase domain promotes autophosphorylation of cyanobacterial clock protein KaiC. Proc Natl Acad Sci U S A..

[78] Egli M, Mori T, Pattanayek R, Xu Y, Qin X, Johnson CH (2012). Dephosphorylation of the core clock protein KaiC in the cyanobacterial KaiABC circadian oscillator proceeds via an ATP synthase mechanism. Biochemistry..

[79] Abe J, Hiyama TB, Mukaiyama A, Son S, Mori T, Saito S, Osako M, Wolanin J, Yamashita E, Kondo T, Akiyama S (2015). Atomic-scale origins of slowness in the cyanobacterial circadian clock. Science..

[80] Murayama Y, Mukaiyama A, Imai K, Onoue Y, Tsunoda A, Nohara A, Ishida T, Maéda Y, Terauchi K, Kondo T, Akiyama S (2011). Tracking and visualizing the circadian ticking of the cyanobacterial clock protein KaiC in solution. EMBO J..

[81] Williams SB, Vakonakis I, Golden SS, LiWang AC (2002). Structure and function from the circadian clock protein KaiA of Synechococcus elongatus: a potential clock input mechanism. Proc Natl Acad Sci U S A..

[82] Kitayama Y, Iwasaki H, Nishiwaki T, Kondo T (2003). KaiB functions as an attenuator of KaiC phosphorylation in the cyanobacterial circadian clock system. EMBO J..

[83] Pattanayek R, Williams DR, Pattanayek S, Xu Y, Mori T, Johnson CH, Stewart PL, Egli M (2006). Analysis of KaiA-KaiC protein interactions in the cyanobacterial circadian clock using hybrid structural methods. EMBO J..

[84] Kim YI, Dong G, Carruthers CW, Golden SS, LiWang A (2008). The day/night switch in KaiC, a central oscillator component of the circadian clock of Cyanobacteria. Proc Natl Acad Sci U S A..

[85] Snijder J, Schuller JM, Wiegard A, Lössl P, Schmelling N, Axmann IM, Plitzko JM, Förster F, Heck AJ (2017). Structures of the cyanobacterial circadian oscillator frozen in a fully assembled state. Science..

[86] Chang YG, Kuo NW, Tseng R, LiWang A (2011). Flexibility of the C-terminal, or CII, ring of KaiC governs the rhythm of the circadian clock of cyanobacteria. Proc Natl Acad Sci U S A..

[87] Murzin AG (2008). Metamorphic proteins. Science..

[88] Chang Y-G, Cohen SE, Phong C, Myers WK, Kim Y-I, Tseng R, Lin J, Zhang L, Boyd JS, Lee Y, Kang S, Lee D, Li S, Britt RD, Rust MJ, Golden SS, LiWang A (2015). A protein fold switch joins the circadian oscillator to clock output in cyanobacteria. Science..

[89] Vakonakis I, Klewer DA, Williams SB, Golden SS, LiWang AC (2004). Structure of the N-terminal domain of the circadian clock-associated histidine kinase SasA. J Mol Biol..

[90] Tseng R, Chang YG, Bravo I, Latham R, Chaudhary A, Kuo NW, Liwang A (2014). Cooperative KaiA-KaiB-KaiC interactions affect KaiB/SasA competition in the circadian clock of cyanobacteria. J Mol Biol..

[91] Mukaiyama A, Furuike Y, Abe J, Yamashita E, Kondo T, Akiyama S (2018). Conformational rearrangements of the C1 ring in KaiC measure the timing of the assembly with KaiB. Sci Rep..

[92] Johnson CJ, Stewart PL, Egli M (2011). The Cyanobacterial Circadian System: From Biophysics to Bioevolution. Annu Rev of Biophysics..

[93] Shi Y (2009). Serine/threonine phosphatase: Mechanism through structure. Cell..

[94] Loros JJ, Dunlap JC (2001). Genetic and molecular analysis of circadian rhythms in Neurospora. Annu Rev Physiol..

[95] Dunlap JC, Loros JJ (2004). The Neurospora circadian system. J Biol Rhythms..

[96] Froehlich AC, Liu Y, Loros JJ, Dunlap JC (2002). White Collar-1, a circadian blue light photoreceptor, binding to the frequency promoter. Science..

[97] Cheng P, He Q, Wang L, Liu Y (2005). Regulation of the Neurospora circadian clock by an RNA helicase. Genes Dev..

[98] Lee K, Loros JJ, Dunlap JC (2000). Interconnected feedback loops in the Neurospora circadian system. Science..

[99] Cheng P, Yang Y, Gardner KH, Liu Y (2002). PAS domain-mediated WC-1/WC−2 interaction is essential for maintaining the steady-state level of WC-1 and the function of both proteins in circadian clock and light responses of Neurospora. Mol Cell Biol..

[100] Cheng P, Yang Y, Wang L, He Q, Liu Y (2003). WHITE COLLAR-1, a multifunctional Neurospora protein involved in the circadian feedback loops, light sensing, and transcription repression of wc-2. J Biol Chem..

[101] Crosthwaite SK, Dunlap JC, Loros JJ (1997). Neurospora wc-1 and wc-2: transcription, photoresponses, and the origins of circadian rhythmicity. Science..

[102] Linden H, Macino G (1997). White collar 2, a partner in blue-light signal transduction, controlling expression of light-regulated genes in Neurospora crassa. EMBO J..

[103] Schafmeier T, Haase A, Káldi K, Scholz J, Fuchs M, Brunner M (2005). Transcriptional feedback of Neurospora circadian clock gene by phosphorylation-dependent inactivation of its transcription factor. Cell..

[104] He Q, Cha J, He Q, Lee H, Yanf Y, Liu Y (2006). CKI and CKII mediate the frequency-dependent phosphorylation of the white collar complex to close the Neurospora circadian negative feedback loop. Genes Dev..

[105] Pando MP, Sassone-Corsi P (2001). Molecular clocks. A vivid loop of light. Nature..

[106] Zoltowski BD, Schwerdtfeger C, Widom J, Loros JJ, Bilwes AM, Dunlap JC, Crane BR (2007). Conformational switching in the fungal light sensor Vivid. Science..

[107] Schwerdtfeger C, Linden H (2003). VIVID is a flavoprotein and serves as a fungal blue light receptor for photoadaptation. EMBO J..

[108] Konopka RJ, Benzer S. Clock mutants of Drosophila melanogaster. Proc Natl Acad Sci U S A. 1971;68:2112–6. 10.1073/pnas.68.9.2112 PMC389363 5002428

[109] Hall JC (2003). Genetics and molecular biology of rhythms in Drosophila and other insects. Adv Genet..

[110] King DP, Zhao Y, Sangoram AM, Wilsbacher LD, Tanaka M, Antoch MP, Steeves TD, Vitaterna MH, Kornhauser JM, Lowrey PL, Turek FW, Takahashi JS (1997). Positional cloning of the mouse circadian clock gene. Cell..

[111] Gekakis N, Staknis D, Nguyen HB, Davis FC, Wilsbacher LD, King DP, Takahashi JS, Weitz CJ (1998). Role of the CLOCK protein in the mammalian circadian mechanism. Science..

[112] Kume K, Zylka MJ, Sriram S, Shearman LP, Weaver DR, Jin X, Maywood ES, Hastings MH, Reppert SM (1999). mCRY1 and mCRY2 are essential components of the negative limb of the circadian clock feedback loop. Cell..

[113] Bunger MK, Wilsbacher LD, Moran SM, Clendenin C, Radcliffe LA, Hogenesch JB, Simon MC, Takahashi JS, Bradfield CA (2000). Mop3 is an essential component of the master circadian pacemaker in mammals. Cell..

[114] Zheng B, Albrecht U, Kaasik K, Sage M, Lu W, Vaishnav S, Li Q, Sun ZS, Eichele G, Bradley A, Lee CC (2001). Nonredundant roles of the mPer1 and mPer2 genes in the mammalian circadian clock. Cell..

[115] Hardin PE (2004). Transcription regulation within the circadian clock: the E-box and beyond. J Biol Rhythms..

[116] Stanewsky R (2003). Genetic analysis of the circadian system in Drosophila melanogaster and mammals. J Neurobiol..

[117] Okamura H, Miyake S, Sumi Y, Yamaguchi S, Yasui A, Muijtjens M, Hoeijmakers JH, van der Horst GT (1999). Photic induction of mPer1 and mPer2 in Cry-deficient mice lacking a biological clock. Science..

[118] Lee C, Etchegaray JP, Cagampang FR, Loudon AS, Reppert SM (2001). Posttranslational mechanisms regulate the mammalian circadian clock. Cell..

[119] Sato TK, Yamada RG, Ukai H, Baggs JE, Miraglia LJ, Kobayashi TJ, Welsh DK, Kay SA, Ueda HR, Hogenesch JB (2006). Feedback repression is required for mammalian circadian clock function. Nat Genet..

[120] Gotter AL (2006). A Timeless debate: resolving TIM's noncircadian roles with possible clock function. Neuroreport..

[121] Barnes JW, Tischkau SA, Barnes JA, Mitchell JW, Burgoon PW, Hickok JR, Gillette MU (2003). Requirement of mammalian Timeless for circadian rhythmicity. Science..

[122] Gotter AL, Manganaro T, Weaver DR, Kolakowski LF, Possidente B, Sriram S, MacLaughlin DT, Reppert SM (2000). A time-less function for mouse timeless. Nat Neurosci..

[123] Thresher RJ, Vitaterna MH, Miyamoto Y, Kazantsev A, Hsu DS, Petit C, Selby CP, Dawut L, Smithies O, Takahashi JS, Sancar A (1998). Role of mouse cryptochrome blue-light photoreceptor in circadian photoresponses. Science..

[124] Stanewsky R, Kaneko M, Emery P, Beretta B, Wager-Smith K, Kay SA, Rosbash M, Hall JC (1998). The cry^b^ mutation identifies cryptochrome as a circadian photoreceptor in Drosophila. Cell..

[125] Cashmore AR (2003). Cryptochromes: Enabling plants and animals to determine circadian time. Cell..

[126] Van der Horst GT, Muijtjens M, Kobayashi K, Takano R, Kanno S, Takao M, de Wit J, Verkerk A, Eker AP, van Leenen D, Buijs R, Bootsma D, Hoeijmakers JH, Yasui A (1999). Mammalian Cry1 and Cry2 are essential for maintenance of circadian rhythms. Nature..

[127] Griffin EAJ, Staknis D, Weitz CJ (1999). Light-independent role of CRY1 and CRY2 in the mammalian circadian clock. Science..

[128] Preitner N, Damiola F, Lopez-Molina L, Zakany J, Duboule D, Albrecht U, Schibler U. The orphan nuclear receptor REV-ERBα controls circadian transcription within the positive limb of the mammalian circadian oscillator. Cell. 2002;110:251–60. 10.1016/s0092-8674(02)00825-5 12150932

[129] Sato TK, Panda S, Miraglia LJ, Reyes TM, Rudic RD, McNamara P, Naik KA, FitzGerald GA, Kay SA, Hogenesch JB (2004). A functional genomics strategy reveals RORa as a component of the mammalian circadian clock. Neuron..

[130] Triqueneaux G, Thenot S, Kakizawa T, Antoch MP, Safi R, Takahashi JS, Delaunay F, Laudet V (2004). The orphan receptor Rev-erba gene is a target of the circadian clock pacemaker. J Mol Endocrinol..

[131] Akashi M, Takumi T (2005). The orphan nuclear receptor RORa regulates circadian transcription of the mammalian core-clock Bmal1. Nat StructMol Biol..

[132] Guillaumond F, Dardente H, Giguere V, Cermakian N (2005). Differential control of Bmal1 circadian transcription by REV-ERB and ROR nuclear receptors. J Biol Rhythms..

[133] Liu AC, Tran HG, Zhang EE, Priest AA, Welsh DK, Kay SA (2008). Redundant function of REV-ERBa and b and non-essential role for Bmal1 cycling in transcriptional regulation of intracellular circadian rhythms. PLoS Genet..

[134] Blau J, Young MW (1999). Cycling vrille expression is required for a functional Drosophila clock. Cell..

[135] Cyran SA, Buchsbaum AM, Reddy KL, Lin MC, Glossop NR, Hardin PE, Young MW, Storti RV, Blau J (2003). vrille, Pdp1, and dClock form a second feedback loop in the Drosophila circadian clock. Cell..

[136] Yildiz O, Doi M, Yujnovsky I, Cardone L, Berndt A, Hennig S, Schulze S, Urbanke C, Sassone-Corsi P, Wolf E (2005). Crystal structure and interactions of the PAS repeat region of the Drosophila clock protein PERIOD. Mol Cell..

[137] Taylor BL, Zhulin IB (1999). PAS domains: internal sensors of oxygen, redox potential, and light. Microbiol Mol Biol Rev..

[138] Huang ZJ, Edery I, Rosbash M (1993). PAS is a dimerization domain common to Drosophila period and several transcription factors. Nature..

[139] Huang ZJ, Curtin KD, Rosbash M (1995). PER protein interactions and temperature compensation of a circadian clock in Drosophila. Science..

[140] Zylka MJ, Shearman LP, Levine JD, Jin X, Weaver DR, Reppert SM (1998). Molecular analysis of mammalian timeless. Neuron..

[141] Field MD, Maywood ES, O’Brien JA, Weaver DR, Reppert SM, Hastings MH (2000). Analysis of clock proteins in mouse SCN demonstrates phylogenetic divergence of the circadian clockwork and resetting mechanisms. Neuron..

[142] Yagita K, Yamaguchi S, Tamanini F, Der Horst GT, Hoeijmakers JH, Yasui A, Loros JJ, Dunlap JC, Okamura H (2000). Dimerization and nuclear entry of mPER proteins in mammalian cells. Genes Dev..

[143] Loop S, Pieler T (2005). Nuclear import of mPER3 in Xenopus oocytes and HeLa cells requires complex formation with mPER1. FEBS J..

[144] Saez L, Young MW (1996). Regulation of nuclear entry of the Drosophila clock proteins period and timeless. Neuron..

[145] Gekakis N, Saez L, Delahaye-Brown AM, Myers MP, Sehgal A, Young MW, Weitz CJ (1995). Isolation of timeless by PER protein interaction: defective interaction between timeless protein and long-period mutant PERL. Science..

[146] Nawathean P, Rosbash M (2004). The doubletime and CKII kinases collaborate to potentiate Drosophila PER transcriptional repressor activity. Mol Cell..

[147] Rothenfluh A, Young MW, Saez L (2000). A TIMELESS-independent function for PERIOD proteins in the Drosophila clock. Neuron..

[148] Shafer OT, Rosbash M, Truman JW (2002). Sequential nuclear accumulation of the clock proteins period and timeless in the pacemaker neurons of Drosophila melanogaster. J Neurosci..

[149] Chang DC, Reppert SM (2003). A novel C-terminal domain of Drosophila PERIOD inhibits dCLOCK: CYCLE-mediated transcription. Curr Biol..

[150] Meyer P, Saez L, Young MW (2006). PER-TIM interactions in living Drosophila cells: an interval timer for the circadian clock. Science..

[151] Landskron J, Chen KF, Wolf E, Stanewsky R (2009). A role for the PERIOD:PERIOD homodimer in the Drosophila circadian clock. PLoS Biol..

[152] Card PB, Erbel PJ, Gardner KH (2005). Structural basis of ARNT PAS-B dimerization: use of a common beta-sheet interface for hetero- and homodimerization. J Mol Biol..

[153] Schmutz I, Ripperger JA, Baeriswyl-Aebischer S, Albrecht U (2010). The mammalian clock component PERIOD2 coordinates circadian output by interaction with nuclear receptors. Genes Dev..

[154] Bae K, Jin X, Maywood ES, Hastings MH, Reppert SM, Weaver DR (2001). Differential functions of mPer1, mPer2, and mPer3 in the SCN circadian clock. Neuron..

[155] Zheng BH, Larkin DW, Albrecht U, Sun ZS, Sage M, Eichele G, Lee CC, Bradley A (1999). The mPer2 gene encodes a functional component of the mammalian circadian clock. Nature..

[156] Shearman LP, Jin X, Lee C, Reppert SM, Weaver DR (2000). Targeted disruption of the mPer3 gene: Subtle effects on circadian clock function. Mol Cell Biol..

[157] Hasan S, van der Veen DR, Winsky-Sommerer R, Dijk DJ, Archer SN (2011). Altered sleep and behavioral activity phenotypes in PER3-deficient mice. Am J Physiol Regul Integr Comp Physiol..

[158] Costa MJ, So AY, Kaasik K, Krueger KC, Pillsbury ML, Fu YH, Ptacek LJ, Yamamoto KR, Feldman BJ (2011). Circadian rhythm gene Period 3 is an inhibitor of the adipocyte cell fate. J Biol Chem..

[159] Richards J, Jeffers LA, All SC, Cheng K, Gumz ML (2013). Role of Per1 and the mineralocorticoid receptor in the coordinate regulation of αENaC in renal cortical collecting duct cells. Front Physiol..

[160] Ramanathan C, Xu H, Khan SK, Shen Y, Gitis PJ, Welsh DK, Hogenesch JB, Liu AC (2014). Cell type-specific functions of period genes revealed by novel adipocyte and hepatocyte circadian clock models. PLoS Genet..

[161] Huang N, Chelliah Y, Shan Y, Taylor CA, Yoo SH, Partch C, Green CB, Zhang H, Takahashi JS (2012). Crystal structure of the heterodimeric CLOCK:BMAL1 transcriptional activator complex. Science..

[162] Nair SK, Burley SK (2003). X-ray structures of Myc-Max and Mad-Max recognizing DNA. Molecular bases of regulation by proto-oncogenic transcription factors. Cell..

[163] Chapman-Smith A, Whitelaw ML (2006). Novel DNA binding by a basic helix-loop-helix protein. The role of the dioxin receptor PAS domain. J Biol Chem..

[164] Ni M, Tepperman JM, Quail PH (1998). PIF3, a phytochrome-interacting factor necessary for normal photoinduced signal transduction, is a novel basic helix-loop-helix protein. Cell..

[165] Yakir E, Hilman D, Kron I, Hassidim M, Melamed-Book N, Green RM (2009). Posttranslational Regulation of CIRCADIAN CLOCK ASSOCIATED1 in the circadian oscillator of Arabidopsis. Plant Physiol..

[166] Woo E, Jeong DG, Lim M, Kim SJ, Kim K, Yoon S, Park B, Ryu SE (2007). Structural insight into the constitutive repression function of the nuclear receptor Rev-erbβ. J Mol Biol..

[167] Duez H, Staels B (2008). The nuclear receptors Rev-erbs and RORs integrate circadian rhythms and metabolism. Diabetes Vasc Dis Res..

[168] Harding HP, Lazar MA (1995). The monomer-binding orphan receptor Rev-Erb represses transcription as a dimer on a novel direct repeat. Mol Cell Biol..

[169] Reinking J, Lam MM, Pardee K, Sampson HM, Liu S, Yang P, Williams S, White W, Lajoie G, Edwards A, Krause HM (2005). The Drosophila nuclear receptor e75 contains heme and is gas responsive. Cell..

[170] Raghuram S (2007). Identification of heme as the ligand for the orphan nuclear receptors REV-ERB[agr] and REV-ERB. Nat Struct Mol Biol..

[171] Yin L, Wu N, Curtin JC, Qatanani M, Szwergold NR, Reid RA, Waitt GM, Parks DJ, Pearce KH, Wisely GB, Lazar MA (2007). Rev-erbα, a heme sensor that coordinates metabolic and circadian pathways. Science..

[172] Pardee KI, Xu X, Reinking J, Schuetz A, Dong A, Liu S, Zhang R, Tiefenbach J, Lajoie G, Plotnikov AN, Botchkarev A, Krause mail HM, Edwards A (2009). The structural basis of gas-responsive transcription by the human nuclear hormone receptor REV-ERBβ. PLoS Biol..

[173] Dioum EM, Rutter J, Tuckerman JR, Gonzalez G, Gilles-Gonzalez MA, McKnight SL (2002). NPAS2: a gas-responsive transcription factor. Science..

[174] Yin L, Lazar MA (2005). The orphan nuclear receptor Rev-erbα recruits the N-CoR/ histone deacetylase 3 corepressor to regulate the circadian Bmal1 gene. Mol Endocrinol..

[175] Zamir I, Dawson J, Lavinsky RM, Glass CK, Rosenfeld MG, Lazar MA (1997). Cloning and characterization of a corepressor and potential component of the nuclear hormone receptor repression complex. Proc Natl Acad Sci U S A..

[176] Hu X, Lazar MA (1999). The CoRNR motif contols the recruitment of corepressors to nuclear hormone receptors. Nature..

[177] Nagy L, Kao HY, Love JD, Li C, Banayo E, Gooch JT, Krishna V, Chatterjee K, Evans RM, Schwabe JW (1999). Mechanism of corepressor binding and release from nuclear hormone receptors. Genes Dev..

[178] Perissi V, Staszewski LM, McInerney EM, Kurokawa R, Krones A, Rose DW, Lambert MH, Milburn MV, Glass CK, Rosenfeld MG (1999). Molecular determinants of nuclear receptor-corepressor interaction. Genes Dev..

[179] Phelan CA, Gampe RT, Lambert MH, Parks DJ, Montana V, Bynum J, Broderick TM, Hu X, Williams SP, Nolte RT, Lazar MA (2010). Structure of REV-ERBα bound to N-CoR reveals a unique mechanism of nuclear receptor-co-repressor interaction. Nat Struct Mol Biol..

[180] Cho H, Zhao X, Hatori M, Yu RT, Barish GD, Lam MT, Chong LW, DiTacchio L, Atkins AR, Glass CK, Liddle C, Auwerx J, Downes M, Panda S, Evans RM (2012). Regulation of circadian behaviour and metabolism by REV-ERB-α and REV-ERB-β. Nature..

[181] Rey G, Cesbron F, Rougemont J, Reinke H, Brunner M, Naef F (2011). Genome-wide and phase-specific DNA-binding rhythms of BMAL1 control circadian output functions in mouse liver. PLoS Biol..

[182] Solt LA, Wang Y, Banerjee S, Hughes T, Kojetin DJ, Lundasen T, Shin Y, Liu J, Cameron MD, Noel R, Yoo SH, Takahashi JS, Butler AA, Kamenecka TM, Burris TP (2012). Regulation of circadian behaviour and metabolism by synthetic REV-ERB agonists. Nature..

[183] Locke JCW, Kozma-Bognar L, Gould PD, Feher B, Kevei E, Nagy F, Turner MS, Hall A, Millar AJ (2006). Experimental validation of a predicted feedback loop in the multi-oscillator clock of Arabidopsis thaliana. Mol Syst Biol..

[184] Zeilinger MN, Farre EM, Taylor SR, Kay SA, Doyle FJ (2006). A novel computational model of the circadian clock in Arabidopsis that incorporates PRR7and PRR9. Mol Syst Biol..

[185] Matsushika A, Makino S, Kojima M, Mizuno T (2000). Circadian waves of expression of the APRR1/TOC1 family of pseudo-response regulators in Arabidopsis thaliana: Insight into the plant circadian clock. Plant Cell Physiol..

[186] Farre EM, Kay SA (2007). PRR7 protein levels are regulated by light and the circadian clock in Arabidopsis. Plant J..

[187] Ito S, Nakamichi N, Kiba T, Yamashino T, Mizuno T (2007). Rhythmic and light-inducible appearance of clock-associated pseudo-response regulator protein PRR9 through programmed degradation in the dark in Arabidopsis thaliana. Plant Cell Physiol..

[188] Kiba T, Henriques R, Sakakibara H, Chua NH (2007). Targeted degradation of pseudo-response regulator 5 by an SCFZTL complex regulates clock function and photomorphogenesis in Arabidopsis thaliana. Plant Cell..

[189] Fujiwara S, Wang L, Han L, Suh SS, Salome PA, McClung CR, Somers DE (2008). Post-translational regulation of the Arabidopsis circadian clock through selective proteolysis and phosphorylation of pseudo-response regulator proteins. J Biol Chem..

[190] Strayer C, Oyama T, Schultz TF, Raman R, Somers DE, Mas P, Panda S, Kreps JA, Kay SA (2000). Cloning of the Arabidopsis clock gene TOC1, an autoregulatory response regulator homolog. Science..

[191] Gendron JM, Pruneda-Paz JL, Doherty CJ, Gross AM, Kang SE, Kay SA (2012). Arabidopsis circadian clock protein, TOC1, is a DNA-binding transcription factor. Proc Natl Acad Sci U S A..

[192] Alabadi D, Oyama T, Yanovsky MJ, Harmon FG, Mas P, Kay SA (2001). Reciprocal regulation between TOC1 and LHY/CCA1 within the Arabidopsis circadian clock. Science..

[193] Schaffer R, Ramsay N, Samach A, Corden S, Putterill J, Carre IA, Coupland G (1998). The late elongated hypocotyl mutation of Arabidopsis disrupts circadian rhythms and the photoperiodic control of flowering. Cell..

[194] Wang ZY, Tobin EM (1998). Constitutive expression of the CIRCADIAN CLOCK ASSOCIATED 1 (CCA1) gene disrupts circadian rhythms and suppresses its own expression. Cell..

[195] Farre EM, Harmer SL, Harmon FG, Yanovsky MJ, Kay SA (2005). Overlapping and distinct roles of PRR7 and PRR9 in the Arabidopsis circadian clock. Curr Biol..

[196] Nakamichi N, Kita M, Ito S, Sato E, Yamashino T, Mizuno T (2005). The Arabidopsis pseudo-response regulators, PRR5 and PRR7, co-ordinately play essential roles for circadian clock function. Plant Cell Physiol..

[197] Nakamichi N, Kita M, Ito S, Yamashino T, Mizuno T (2005). PSEUDO-RESPONSE REGULATORS, PRR9, PRR7 and PRR5, together play essential roles close to the circadian clock of Arabidopsis thaliana. Plant Cell Physiol..

[198] Fowler S, Lee K, Onouchi H, Samach A, Richardson K, Morris B, Coupland G, Putterill J (1999). GIGANTEA: a circadian clock-controlled gene that regulates photoperiodic flowering in Arabidopsis and encodes a protein with several possible membrane spanning domains. EMBO J..

[199] Makino S, Matsushika A, Kojima M, Yamashino T, Mizuno T (2002). The APRR1/TOC1 quintet implicated in circadian rhythms of Arabidopsis thaliana: I. Characterization with APRR1-overexpressing plants. Plant Cell Physiol..

[200] Hazen SP, Schultz TF, Pruneda-Paz JL, Borevitz JO, Ecker JR, Kay SA (2005). LUX ARRHYTHMO encodes a Myb domain protein essential for circadian rhythms. Proc Natl Acad Sci U S A..

[201] Onai K, Ishiura M (2005). PHYTOCLOCK 1 encoding a novel GARP protein essential for the Arabidopsis circadian clock. Genes Cells..

[202] McWatters HG, Bastow RM, Hall A, Millar AJ (2000). The ELF3 zeitnehmer regulates light signaling to the circadian clock. Nature..

[203] McWatters HG, Kolmos E, Hall A, Doyle MR, Amasino RM, Gyula P, Nagy F, Miller AJ, Davis SJ (2007). ELF4 is required for oscillatory properties of the circadian clock. Plant Physiol..

[204] Covington MF, Panda S, Liu XL, Strayer CA, Wagner DR, Kay SA (2001). ELF3 modulates resetting of the circadian clock in Arabidopsis. Plant Cell..

[205] Hicks KA, Albertson TM, Wagner DR (2001). EARLY FLOWERING3 encodes a novel protein that regulates circadian clock function and flowering in Arabidopsis. Plant Cell..

[206] Doyle MR, Davis SJ, Bastow RM, McWatters HG, Kozma-Bognar L, Nagy F, Miller AJ, Amasino RM (2002). The ELF4 gene controls circadian rhythms and flowering time in Arabidopsis thaliana. Nature..

[207] Nusinow DA, Helfer A, Hamilton EE, King JJ, Imaizumi T, Schultz TF, Farré EA, Kay SA (2011). The ELF4–ELF3–LUX complex links the circadian clock to diurnal control of hypocotyl growth. Nature..

[208] Thines B, Harmon FG (2010). Ambient temperature response establishes ELF3 as a required component of the core Arabidopsis circadian clock. Proc Natl Acad Sci U S A..

[209] Dixon LE, Knox K, Kozma-Bognar L, Southern MM, Pokhilko A, Millar AJ (2011). Temporal repression of core circadian genes is mediated through EARLY FLOWERING 3 in Arabidopsis. Curr Biol..

[210] Helfer A, Nusinow DA, Chow BY, Gehrke AR, Bulyk ML, Kay SA (2011). LUX ARRHYTHMO encodes a nighttime repressor of circadian gene expression in the Arabidopsis core clock. Curr Biol..

[211] Herrero E, Kolmos E, Bujdoso N, Yuan Y, Wang M, Berns MC, Coupland G, Saini R, Jaskolski M, Webb A, Gonçalves J, Davis SJ (2012). ELF4 recruitment of ELF3 in the nucleus sustains the plant circadian clock. Plant Cell..

[212] Kolmos E, Nowak M, Werner M, Fischer K, Schwarz G, Mathews S, Schoof H, Nagy F, Bujnicki JM, Davis SJ (2009). Integrating ELF4 into the circadian system through combined structural and functional studies. HFSP J..

[213] Kolmos E, Herrero E, Bujdoso N, Millar AJ, Tóth R, Gyula P, Nagy F, Davis SJ (2011). A reduced-function allele reveals that EARLY FLOWERING3 repressive action on the circadian clock is modulated by phytochrome signals in Arabidopsis. Plant Cell..

[214] Kolmos E, Schoof H, Plumer M, Davis SJ (2008). Structural insights into the function of the core-circadian factor TIMING OF CAB2 EXPRESSION 1 (TOC1). J Circadian Rhythms..

[215] Saier MHJ (1994). Bacterial sensor kinase/response regulator systems: an introduction. ResMicrobiol..

[216] West AH, Stock AM (2001). Histidine kinases and response regulator proteins in two-component signaling systems. Trends Biochem Sci..

[217] Hwang I, Chen HC, Sheen J (2002). Two-component signal transduction pathways in Arabidopsis. Plant Physiol..

[218] Varughese KI (2002). Molecular recognition of bacterial phosphorelay proteins. Curr Opin Microbiol..

[219] Wenkel S, Turck F, Singer K, Gissot L, Le Gourrierec J, Samach A, Coupland G (2006). CONSTANS and the CCAAT box binding complex share a functionally important domain and interact to regulate flowering of Arabidopsis. Plant Cell..

[220] Schmitz O, Katayama M, Williams SB, Kondo T, Golden SS (2000). CikA, a bacteriophytochrome that resets the cyanobacterial circadian clock. Science..

[221] Katayama M, Kondo T, Xiong J, Golden SS (2003). ldpA encodes an iron-sulfur protein involved in light-dependent modulation of the circadian period in the cyanobacterium Synechococcus elongatus PCC 7942. J Bacteriol..

[222] Li H, Sherman LA (2000). A redox-responsive regulator of photosynthesis gene expression in the cyanobacterium Synechocystis sp. Strain PCC 6803. J Bacteriol..

[223] Ivleva NB, Gao T, LiWang A, Golden SS (2006). Quinone sensing by the circadian input kinase of the cyanobacterial circadian clock. Proc Natl Acad Sci U S A..

[224] Ivleva NB, Bramlett MR, Lindahl PA, Golden SS (2005). LdpA: a component of the circadian clock senses redox state of the cell. EMBO J..

[225] Mutsuda M, Michel KP, Zhang X, Montgomery BL, Golden SS (2003). Biochemical properties of CikA, an unusual phytochrome-like histidine protein kinase that resets the circadian clock in Synechococcus elongatus PCC 7942. J Biol Chem..

[226] Gao T, Zhang X, Ivleva NB, Golden SS, LiWang A (2007). NMR structure of the pseudo-receiver domain of CikA. Protein Sci..

[227] Marina A, Waldburger CD, Hendrickson WA (2005). Structure of the entire cytoplasmic portion of a sensor histidine-kinase protein. EMBO J..

[228] Varughese KI, Tsigelny I, Zhao HJ (2006). The crystal structure of beryllofluoride Spo0F in complex with the phosphotransferase Spo0B represents a phosphotransfer pretransition state. Bacteriol..

[229] Gutu A, O’Shea EK (2013). Two antagonistic clock-regulated histidine kinases time the activation of circadian gene expression. Mol Cell..

[230] Wood TL, Bridwell-Rabb J, Kim Y, Gao T, Chang Y, LiWang A, Barondeau DP, Golden SS (2010). The KaiA protein of the cyanobacterial circadian oscillator is modulated by a redox-active cofactor. Proc Natl Acad Sci U S A..

[231] He Q, Cheng P, Yang Y, Wang L, Gardner KH, Liu Y (2002). White collar-1, a DNA binding transcription factor and a light sensor. Science..

[232] Heintzen C, Loros JJ, Dunlap JC (2001). The PAS protein VIVID defines a clock-associated feedback loop that represses light input, modulates gating, and regulates clock resetting. Cell..

[233] He Q, Liu Y (2005). Molecular mechanism of light responses in Neurospora: from light-induced transcription to photoadaptation. Genes Dev..

[234] Schafmeier T, Diernfellner AC (2011). Light input and processing in the circadian clock of Neurospora. FEBS Lett..

[235] Kennis JT, Crosson S, Gauden M, van Stokkum IH, Moffat K, van Grondelle R (2003). Primary reactions of the LOV2 domain of phototropin, a plant blue-light photoreceptor. Biochemistry..

[236] Zoltowski BD, Vaccaro B, Crane BR (2009). Mechanism-based tuning of a LOV domain photoreceptor. Nat Chem Biol..

[237] Malzahn E, Ciprianidis S, Kaldi K, Schafmeier T, Brunner M (2010). Photoadaptation in Neurospora by competitive interaction of activating and inhibitory LOV domains. Cell..

[238] Chen CH, DeMay BS, Gladfelter AS, Dunlap JC, Loros JJ (2010). Physical interaction between VIVID and white collar complex regulates photoadaptation in Neurospora. Proc Natl Acad Sci U S A..

[239] Demarsy E, Fankhauser C (2009). Higher plants use LOV to perceive blue light. Curr Opin Plant Biol..

[240] Crosson S, Moffat K (2001). Structure of a flavin-binding plant photoreceptor domain: insights into light-mediated signal transduction. Proc Natl Acad Sci U S A..

[241] Fedorov R, Schlichting I, Hartmann E, Domratcheva T, Fuhrmann M, Hegemann P (2003). Crystal structures and molecular mechanism of a light-induced signaling switch: the Phot-LOV1 domain from Chlamydomonas reinhardtii. Biophys J..

[242] Zoltowski BD, Crane BR (2008). Light activation of the LOV protein Vivid generates a rapidly exchanging dimer. Biochemistry..

[243] Gardner MJ, Hubbard KE, Hotta CT, Dodd AN, Webb AAR (2006). How plants tell the time. Biochem J..

[244] Nagy F, Schafer E (2002). Phytochromes control photomorphogenesis by differentially regulated, interacting signaling pathways in higher plants. Annu Rev Plant Biol..

[245] Quail PH (2002). Phytochrome photosensory signalling networks. Nat Rev Mol Cell Biol..

[246] Lin CT (2002). Blue light receptors and signal transduction. Plant Cell..

[247] Chaves I, Pokorny R, Byrdin M, Hoang N, Ritz T, Brettel K, Essen L-O, van der Horst GTJ, Batschauer A, Ahmad M (2011). The cryptochromes: blue light photoreceptors in plants and animals. Annu Rev Plant Biol..

[248] Kleine T, Lockhart P, Batschauer A (2003). An Arabidopsis protein closely related to Synechocystis cryptochrome is targeted to organelles. Plant J..

[249] Brudler R, Hitomi K, Daiyasu H, Toh H, Kucho K, Ishiura M, Kanehisa M, Roberts VA, Todo T, Tainer JA, Getzoff ED (2003). Identification of a new cryptochrome class. Structure, function, and evolution. Mol Cell..

[250] Somers DE, Schultz TF, Milnamow M, Kay SA (2000). ZEITLUPE encodes a novel clock-associated PAS protein from Arabidopsis. Cell..

[251] Martinez-Garcia JF, Huq E, Quail PH (2000). Direct targeting of light signals to a promoter element-bound transcription factor. Science..

[252] Jarillo JA, Capel J, Tang RH, Yang HQ, Alonso JM, Ecker JR, Cashmore AR (2001). An Arabidopsis circadian clock component interacts with both CRY1 and phyB. Nature..

[253] Ito S, Song YH, Imaizumi T (2012). LOV domain-containing F-box proteins: light-dependent protein degradation modules in Arabidopsis. Mol Plant..

[254] Sawa M, Nusinow DA, Kay SA, Imaizumi T (2007). FKF1 and GIGANTEA complex formation is required for day-length measurement in Arabidopsis. Science..

[255] Kim WY, Geng R, Somers DE (2003). Circadian phase-specific degradation of the F-box protein ZTL is mediated by the proteasome. Proc Natl Acad Sci U S A..

[256] Hall A, Bastow RM, Davis SJ, Hanano S, McWatters HG, Hibberd V, Doyle MR, Sung SB, Halliday KJ, Amasino RM, Millar AJ. The TIME FOR COFFEE gene maintains the amplitude and timing of Arabidopsis circadian clocks. Plant Cell. 2003;15:2719–29. 10.1105/tpc.013730 PMC280574 14555691

[257] Rockwell NC, Lagarias JC (2006). The structure of phytochrome: a picture is worth a thousand spectra. Plant Cell..

[258] Somers DE, Devlin PF, Kay SA (1998). Phytochromes and cryptochromes in the entrainment of the Arabidopsis circadian clock. Science..

[259] Chen C, Loros JJ (2009). Nuerospora sees the light: Light signaling components in a model system. Commun Integr Biol..

[260] Ikeuchi M, Ishizuka T (2008). Cyanobacteriochromes: a new superfamily of tetrapyrrole-binding photoreceptors in Cyanobacteria. Photochem Photobiol Sci..

[261] Hughes J (2010). Phytochrome three-dimensional structures and functions. Biochem Soc Trans.

[262] Wagner JR, Zhang J, Brunzelle JS, Vierstra RD, Forest KT (2007). High resolution structure of Deinococcus bacteriophytochrome yields new insights into phytochrome architecture and evolution. J Biol Chem..

[263] Yang X, Stojkovic EA, Kuk J, Moffat K (2007). Crystal structure of the chromophore binding domain of an unusual bacteriophytochrome, RpBphP3, reveals residues that modulate photoconversion. Proc Natl Acad Sci U S A..

[264] Essen L-O, Mailliet J, Hughes J (2008). The structure of a complete phytochrome sensory module in the Pr ground state. Proc Natl Acad Sci U S A..

[265] Yang X, Kuk J, Moffat K (2008). Crystal structure of Pseudomonas aeruginosa bacteriophytochrome: photoconversion and signal transduction. Proc Natl Acad Sci U S A..

[266] Nagatani A (2010). Phytochrome: structural basis for its functions. Curr Opin Plant Biol..

[267] Nakasako M, Matsuoka D, Zikihara K, Tokutomi S (2005). Quaternary structure of LOV-domain containing polypeptide of Arabidopsis FKF1 protein. FEBS Lett..

[268] Corchnoy SB, Swartz TE, Lewis JW, Szundi I, Briggs WR, Bogomolni RA (2003). Intramolecular proton transfers and structural changes during the photocycle of the LOV2 domain of phototropin 1. J Biol Chem..

[269] Salomon M, Christie JM, Knieb E, Lempert U, Briggs WR (2000). Photochemical and mutational analysis of the FMN binding domains of the plant blue light receptor, phototropin. Biochemistry..

[270] Imaizumi T, Tran HG, Swartz TE, Briggs WR, Kay SA (2003). FKF1 is essential for photoperiodic-specific light signalling in Arabidopsis. Nature..

[271] Kasahara M, Swartz TE, Olney MA, Onodera A, Mochizuki N, Fukuzawa H, Asamizu E, Tabata S, Kanegae H, Takano M, Christie JM, Nagatani A, Briggs WR (2002). Photochemical properties of the flavin mononucleotide-binding domains of the phototropins from Arabidopsis, rice, and Chlamydomonas reinhardtii. Plant Physiol..

[272] Zikihara K, Iwata T, Matsuoka D, Kandori H, Todo T, Tokutomi S (2006). Photoreaction cycle of the light, oxygen, and voltage domain in FKF1 determined by low-temperature absorption spectroscopy. Biochemistry..

[273] Nakasone Y, Zikihara K, Tokutomi S, Terazima M (2010). Kinetics of conformational changes of the FKF1–LOV domain upon photoexcitation. Biophys J..

[274] Salomon M, Lempert U, Rudiger W (2004). Dimerization of the plant photoreceptor phototropin is probably mediated by the LOV1 domain. FEBS Lett..

[275] Eitoku T, Nakasone Y, Zikihara K, Matsuoka D, Tokutomi S, Terazima M (2007). Photochemical intermediates of Arabidopsis phototropin 2 LOV domains associated with conformational changes. J Mol Biol..

[276] Sancar A (2003). Structure and function of DNA photolyase and cryptochrome blue-light photoreceptors. Chem Rev..

[277] Ahmad M, Cashmore AR (1993). HY4 gene of A. thaliana encodes a protein with characteristics of a blue-light photoreceptor. Nature..

[278] Sancar A. Photolyase and cryptochrome blue-light photoreceptors. Adv Protein Chem. 2004;69:73–100. 10.1016/S0065-3233(04)69003-6 15588840

[279] Lin C, Todo T (2005). The cryptochromes. Genome Biol..

[280] Weber S (2005). Light-driven enzymatic catalysis of DNA repair: a review of recent biophysical studies on photolyase. Biochim Biophys Acta..

[281] Lin C, Shalitin D (2003). Cryptochrome structure and signal transduction. Annu Rev Plant Biol..

[282] Brautigam CA, Smith BS, Ma Z, Palnitkar M, Tomchick DR, Machius M, Deisenhofer J (2004). Structure of the photolyase-like domain of cryptochrome 1 from Arabidopsis thaliana. Proc Natl Acad Sci U S A..

[283] Park H-W, Kim S-T, Sancar A, Deisenhofer J (1995). Crystal structure of DNA photolyase from Escherichia coli. Science..

[284] Komori H, Masui R, Kauramitsu S, Yokoyama S, Shibata T, Inoue Y, Miki K (2001). Crystal structure of thermostable DNA photolyase: pyrimidine-dimer recognition mechanism. Proc Natl Acad Sci U S A..

[285] Tamada T, Kitadokoro K, Higuchi Y, Inaka K, Yasui A, de Ruiter PE, Eker APM, Miki K (1997). Crystal structure of DNA photolyase from Anacystis nidulans. Nat Struct Biol..

[286] Yang HQ, Tang RH, Cashmore AR (2001). The signalling mechanism of Arabidopsis CRY1 involves direct interaction with COP1. Plant Cell..

[287] Wang H, Ma LG, Li JM, Zhao HY, Deng XW (2001). Direct interaction of Arabidopsis cryptochromes with COP1 in light control development. Science..

[288] He SB, Wang WX, Zhang JY, Xu F, Lian HL, Li L, Yang HQ (2015). The CNT1 domain of Arabidopsis CRY1 alone is sufficient to mediate blue light inhibition of hypocotyl elongation. Mol Plant..

[289] Wang S, Li L, Xu P, Lian H, Wang W, Xu F, Mao Z, Zhang T, Yang H. CRY1 interacts directly with HBI1 to regulate its transcriptional activity and photomorphogenesis in Arabidopsis. J Exp Bot. 2018. 10.1093/jxb/ery209 PMC6054188 29860272

[290] Friedrichsen DM, Nemhauser J, Muramitsu T, Maloof JN, Alonso J, Ecker JR, Furuya M, Chory J (2002). Three redundant brassinosteroid early response genes encode putative bHLH transcription factors required for normal growth. Genetics..

[291] Bai M-Y, Fan M, Oh E, Wang Z-Y (2012). A triple helix-loop-helix/basic helix-loop-helix cascade controls cell elongation downstream of multiple hormonal and environmental signaling pathways in Arabidopsis. Plant Cell..

[292] Ikeda M, Fujiwara S, Mitsuda N, Ohme-Takagi M (2012). A triantagonistic basic helix-loop-helix system regulates cell elongation in Arabidopsis. Plant Cell..

[293] Liu H, Yu X, Li K, Klejnot J, Yang H, Lisiero D, Lin C (2008). Photoexcited CRY2 interacts with CIB1 to regulate transcription and floral initiation in Arabidopsis. Science..

[294] Emery P, So WV, Kaneko M, Hall JC, Rosbash M (1998). CRY, a Drosophila clock and light-regulated cryptochrome, is a major contributor to circadian rhythm resetting and photosensitivity. Cell..

[295] Ceriani M, Darlington T, Staknis D, Mas P, Petti A, Weitz C, Kay S (1999). Light-dependent sequestration of TIMELESS by CRYPTOCHROME. Science..

[296] Naidoo N, Song W, Hunter-Ensor M, Seghal A (1999). A role for the proteasome in the light response of the timeless clock protein. Science..

[297] Helfrich-Forster C, Stengl M, Homberg U (1998). Organization of the circadian system in insects. Chronobiol Int..

[298] Helfrich-Forster C, Engelmann W, Kumar V (2002). Photoreceptors for the circadian clock of the fruitfly. Biological rhythms.

[299] Veleri S, Rieger D, Helfrich-Forster C, Stanewsky R (2007). Hofbauer-Buchner eyelets affect circadian photosensitivity and coordinates TIM and PER expression in Drosophila clock neurons. J Biol Rhythms..

[300] Rieger D, Stanewsky R, Helfrich-Forster C (2003). Cryptochrome, compound eyes, hofbauer-buchner eyelets, and ocelli play different roles in the entrainment and masking pathway of the locomotor activity rhythm in the fruit fly Drosophila melanogaster. J Biol Rhythms..

[301] Klarsfeld A, Malpel S, Michard-Vanhee C, Picot M, Chelot E, Rouyer F (2004). Novel features of cryptochrome-mediated photoreception in the brain circadian clock of Drosophila. J Neurosci..

[302] Helfrich-Forster C, Winter C, Hofbauer A, Hall J, Stanewsky R (2001). The circadian clock of fruit flies is blind after elimination of all known photoreceptors. Neuron..

[303] Busza A, Emery-Le M, Rosbash M, Emery P (2004). Roles of the two Drosophila CRYPTOCHROME structural domains in circadian photoreception. Science..

[304] Dissel S, Codd V, Fedic R, Garner KJ, Costa R, Kyriacou CP, Rosato E (2004). A constitutively active cryptochrome in Drosophila melanogaster. Nat Neurosci..

[305] Fedele G, Green EW, Rosato E, Kyriacou CP (2014). An electromagnetic field disrupts negative geotaxis in Drosophila via a CRY-dependent pathway. Nat Commun..

[306] Fedele G, Edwards MD, Bhutani S, Hares JM, Murbach M, Green EW, Dissel S, Hastings MH, Rosato E, Kyriacou CP (2014). Genetic analysis of circadian responses to low frequency electromagnetic fields in Drosophila melanogaster. PLoS Genet..

[307] Yoshii T, Ahmad M, Helfrich-Forster C (2009). Cryptochrome mediates light-dependent magnetosensitivity of Drosophila's circadian clock. PLoS Biol..

[308] Zoltowski BD, Vaidya AT, Top D, Widom J, Young MW, Crane BR (2011). Structure of full-length Drosophila cryptochrome. Nature..

[309] Levy C, Zoltowski BD, Jones AR, Vaidya AT, Top D, Widom J, Young MW, Scrutton NS, Crane BR, Leys D (2013). Updated structure of Drosophila cryptochrome. Nature..

[310] Czarna A, Berndt A, Singh HR, Grudziecki A, Ladurner AG, Timinszky G (2013). Structures of Drosophila cryptochrome and mouse cryptochrome1 provide insight into circadian function. Cell..

[311] Xing W, Busino L, Hinds TR, Marionni ST, Saifee NH (2013). SCF(FBXL3) ubiquitin ligase targets cryptochromes at their cofactor pocket. Nature..

[312] Hitomi K, DiTacchio L, Arvai AS, Yamamoto J, Kim ST, Todo T, Tainer JA, Iwai S, Panda S, Getzoff ED (2009). Functional motifs in the (6-4) photolyase crystal structure make a comparative framework for DNA repair photolyases and clock cryptochromes. Proc Natl Acad Sci U S A..

[313] McCarthy EV, Baggs JE, Geskes JM, Hogenesch JB, Green CB (2009). Generation of a novel allelic series of cryptochrome mutants via mutagenesis reveals residues involved in protein-protein interaction and CRY2-specific repression. Mol Cell Biol..

[314] Czarna A, Breitkreuz H, Mahrenholz CC, Arens J, Strauss HM, Wolf E (2011). Quantitative analyses of cryptochrome-mBMAL1 interactions: mechanistic insights into the transcriptional regulation of the mammalian circadian clock. J Biol Chem..

[315] Lamia KA, Sachdeva UM, DiTacchio L, Williams EC, Alvarez JG, Egan DF, Vasquez DS, Juguilon H, Panda S, Shaw RJ (2009). AMPK regulates the circadian clock by cryptochrome phosphorylation and degradation. Science..

[316] Lamia KA, Papp SJ, Yu RT, Barish GD, Uhlenhaut NH, Jonker JW, Downes M, Evans RM (2011). Cryptochromes mediate rhythmic repression of the glucocorticoid receptor. Nature..

[317] Hirota T, Lee JW, St John PC, Sawa M, Iwaisako K, Noguchi T, Pongsawakul PY, Sonntag T, Welsh DK, Brenner DA (2012). Identification of small molecule activators of cryptochrome. Science..

[318] Zhang EE, Liu Y, Dentin R, Pongsawakul PY, Liu AC, Hirota T, Nusinow DA, Sun X, Landais S, Kodama Y (2010). Cryptochrome mediates circadian regulation of cAMP signaling and hepatic gluconeogenesis. Nat Med..

[319] Foster RG, Provencio I, Hudson D, Fiske S, De Grip W, Menaker M (1991). Circadian photoreception in the retinally degeneratemouse (rd/rd). J Comp Physiol A Sens Neural Behav Physiol..

[320] Freedman MS, Lucas RJ, Soni B, von Schantz M, Munoz M, David-Gray Z, Foster R (1999). Regulation of mammalian circadian behavior by non-rod, non-cone, ocular photoreceptors. Science..

[321] Johnsson A, Engelmann W, Björn LO (2008). The biological clock and its resetting by light. Photobiology, the science of life and light.

[322] Golombek DA, Rosenstein RE. Physiology of circadian entrainment. Physiol Rev. 2010;90:1063–102. 10.1152/physrev.00009.2009 20664079

[323] Panda S, Sato TK, Castrucci AM, Rollag MD, DeGrip WJ, Hogenesch JB, Provencio I, Kay SA (2002). Melanopsin (Opn4) requirement for normal light-induced circadian phase shifting. Science..

[324] Thompson CL, Bowes Rickman C, Shaw SJ, Ebright JN, Kelly U, Sancar A, Rickman DW (2003). Expression of the blue-light receptor cryptochrome in the human retina. Invest Ophthalmol Vis Sci..

[325] Hoang N, Schleicher E, Kacprzak S, Bouly J-P, Picot M, Wu W, Berndt A, Wolf E, Bittl R, Ahmad M. Human and Drosophila cryptochromes are light activated by flavin photoreduction in living cells. PLoS Biol. 2008;6:e160. 10.1371/journal.pbio.0060160 PMC2443192 18597555

[326] Dkhissi-Benyahya O, Gronfier C, Vanssay WD, Flamant F, Cooper HM (2007). Modeling the role of mid-wavelength cones in circadian responses to light. Neuron..

[327] Kim EY, Ko HW, Yu W, Hardin PE, Edery I (2007). A DOUBLETIME kinase binding domain on the Drosophila PERIOD protein is essential for its hyperphosphorylation, transcriptional repression, and circadian clock function. Mol Cell Biol..

[328] Wang L, Fujiwara S, Somers DE (2010). PRR5 regulates phosphorylation, nuclear import and subnuclear localization of TOC1 in the Arabidopsis circadian clock. EMBO J..

[329] Takahashi JS, Hong HK, Ko CH, McDearmon EL (2008). The genetics of mammalian circadian order and disorder: Implications for physiology and disease. Nat Rev Genet..

[330] Stokkan KA, Yamazaki S, Tei H, Sakaki Y, Menaker M (2001). Entrainment of the circadian clock in the liver by feeding. Science..

[331] Lamia KA (2009). AMPK regulates the circadian clock by cryptochrome phosphorylation and degradation. Science..

[332] Nangle S, Xing W, Zheng N (2013). Crystal structure of mammalian cryptochrome in complex with a small molecule competitor of its ubiquitin ligase. Cell Res..

[333] Pettersen EF, Goddard TD, Huang CC, Couch GS, Greenblatt DM, Meng EC, Ferrin TE (2004). UCSF chimera--a visualization system for exploratory research and analysis. J Comput Chem..

[334] Berman HM, Westbrook J, Feng Z, Gilliland G, Bhat TN, Weissig H, Shindyalov IN, Bourne PE (2000). The Protein Data Bank. Nucleic Acids Res..

[335] Jo S, Vargyas M, Vasko-Szedlar J, Roux B, Im W (2008). PBEQ-Solver for online visualization of electrostatic potential of biomolecules. Nucleic Acids Res..

